# Leukotriene B_4_-Neutrophil Elastase Axis Drives Neutrophil Reverse Transendothelial Cell Migration In Vivo

**DOI:** 10.1016/j.immuni.2015.05.010

**Published:** 2015-06-16

**Authors:** Bartomeu Colom, Jennifer V. Bodkin, Martina Beyrau, Abigail Woodfin, Christiane Ody, Claire Rourke, Triantafyllos Chavakis, Karim Brohi, Beat A. Imhof, Sussan Nourshargh

**Affiliations:** 1 William Harvey Research Institute, Barts and The London School of Medicine and Dentistry, Queen Mary University of London, Charterhouse Square, London EC1M 6BQ, UK; 2 Centre Médical Universitaire, Rue Michel-Servet 1, Geneva CH-1211, Switzerland; 3 Centre for Trauma Sciences, Barts and The London School of Medicine and Dentistry, Queen Mary University of London, Mile End Road, London E1 4NS, UK; 4 Department of Clinical Pathobiochemistry, University of Dresden, Fetscherstrasse 74, Dresden 01307, Germany

## Abstract

Breaching endothelial cells (ECs) is a decisive step in the migration of leukocytes from the vascular lumen to the extravascular tissue, but fundamental aspects of this response remain largely unknown. We have previously shown that neutrophils can exhibit abluminal-to-luminal migration through EC junctions within mouse cremasteric venules and that this response is elicited following reduced expression and/or functionality of the EC junctional adhesion molecule-C (JAM-C). Here we demonstrate that the lipid chemoattractant leukotriene B_4_ (LTB_4_) was efficacious at causing loss of venular JAM-C and promoting neutrophil reverse transendothelial cell migration (rTEM) in vivo. Local proteolytic cleavage of EC JAM-C by neutrophil elastase (NE) drove this cascade of events as supported by presentation of NE to JAM-C via the neutrophil adhesion molecule Mac-1. The results identify local LTB_4_-NE axis as a promoter of neutrophil rTEM and provide evidence that this pathway can propagate a local sterile inflammatory response to become systemic.

## Highlights

• Endogenous LTB_4_ mediates reduced expression of endothelial cell (EC) JAM-C in I-R
• LTB_4_ can stimulate proteolytic cleavage of EC JAM-C by neutrophil elastase (NE)
• Activation of a local LTB_4_-NE axis induces neutrophil reverse TEM in vivo
• Activation of a local LTB_4_-NE axis can promote distant organ damage

## Graphical Abstract

**Figure:**
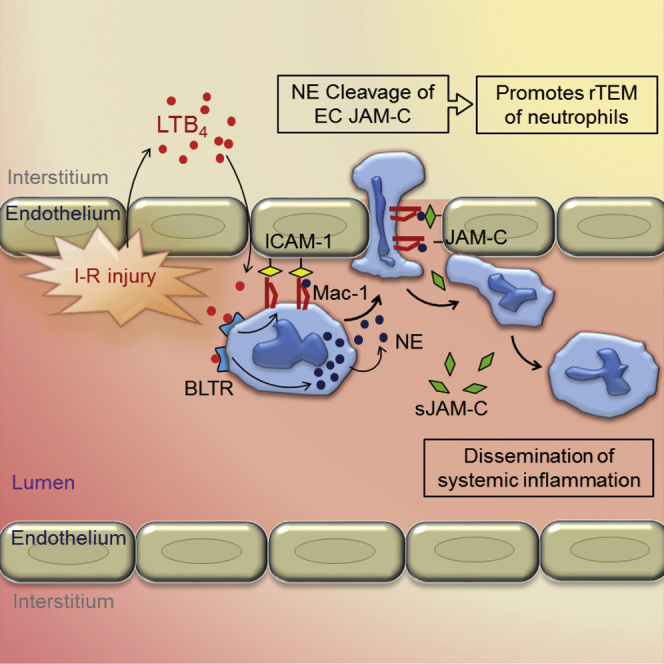

## Teaser

The mechanisms and implications of aberrant modes of neutrophil transendothelial cell migration remain largely unknown. Here, Nourshargh and colleagues demonstrate that local leukotriene B_4_-induced proteolytic cleavage of endothelial JAM-C by neutrophil elastase promotes neutrophil reverse transendothelial cell migration back into the circulation and is decisive in dissemination of systemic inflammation.

## Introduction

Neutrophil infiltration into interstitial tissues is a critical component of the innate immune response and a hallmark of acute inflammatory reactions. Due to the destructive potential of neutrophils, this response is also intimately associated with the pathogenesis of numerous inflammatory conditions such as ischemia-reperfusion (I-R) injury, rheumatoid arthritis, and atherosclerosis (Nathan, 2006; Phillipson and Kubes, 2011; Mócsai, 2013). Neutrophil migration out of the vasculature is classically described by the leukocyte adhesion cascade that depicts a well characterized sequence of cellular and molecular events within the vascular lumen as orchestrated by numerous stimulatory and adhesive pathways (Ley et al., 2007). Less is known about the stages beyond the vascular lumen, though there is a growing understanding of the adhesive interactions that mediate neutrophil interactions with components of venular walls (Ley et al., 2007; Nourshargh et al., 2010; Proebstl et al., 2012; Nourshargh and Alon, 2014) and the molecular and cellular regulation of neutrophil motility in the interstitial tissue (Lämmermann et al., 2013; Weninger et al., 2014).

The migration of neutrophils through the endothelial cell (EC) barrier can occur via both paracellular and transcellular modes (Nourshargh et al., 2010; Kolaczkowska and Kubes, 2013) though the former is considered to be the most prevalent in the peripheral circulation (Schulte et al., 2011; Woodfin et al., 2011). This response is known to be mediated by numerous EC junctional molecules including platelet endothelial cell adhesion molecule-1 (PECAM-1), members of the junctional adhesion molecule (JAM) family, ICAM-2, VE-cadherin, and CD99 (Nourshargh et al., 2010; Voisin and Nourshargh, 2013; Vestweber et al., 2014). In addition to moving from the vascular lumen to the extravascular tissue, there is now unequivocal evidence for the ability of neutrophils to exhibit reverse motility through the endothelium. Specifically, through the application of a high resolution confocal intravital microscopy (IVM) platform to analysis of leukocyte transmigration in the mouse cremaster muscle, we have noted that neutrophils can exhibit migration through EC junctions in an abluminal-to-luminal direction (Woodfin et al., 2011). This neutrophil rTEM response is most prevalent in tissues subjected to the sterile injury caused by I-R, an inflammatory insult that is associated with reduced expression of JAM-C at EC junctions (Scheiermann et al., 2009; Woodfin et al., 2011). Furthermore, pharmacological blockade or genetic deletion of EC JAM-C enhances the frequency of neutrophil rTEM through cremasteric venules (Woodfin et al., 2011) and blockade of EC JAM-C has been shown to promote monocyte rTEM through cultured human umbilical vein ECs (HUVECs) (Bradfield et al., 2007). Together, these results have identified EC junctional JAM-C as a regulator of polarized movement of leukocytes from the vascular lumen toward the sub-EC space. While the phenomenon of neutrophil rTEM has been illustrated using in vitro models of neutrophil TEM (Buckley et al., 2006) and subsequently in vivo within zebrafish embryos (Mathias et al., 2006), our findings within the mouse cremaster muscle provide direct evidence for neutrophil rTEM within a mammalian system, urging a need for better understanding of this unexpected response (Woodfin et al., 2011).

To acquire a greater insight into the frequency, regulation, and pathophysiological role of neutrophil rTEM here, we sought to identify the inflammatory trigger(s) that promote neutrophil rTEM in response to I-R. Specifically, because reduced expression and/or functionality of EC JAM-C was instrumental in promoting neutrophil rTEM in vivo, we investigated the mechanism through which EC JAM-C was lost at sites of sterile injury. The results identified endogenous leukotriene B_4_ (LTB_4_) as the mediator responsible for I-R-elicited loss of venular JAM-C and showed that exogenous LTB_4_ was highly efficacious at lowering the expression of EC JAM-C in vivo. This effect was neutrophil-dependent, with neutrophil elastase (NE) governing the cleavage of EC JAM-C at sites of intense neutrophil infiltration. Furthermore, local LTB_4_ and NE could both promote notable neutrophil rTEM. Although investigations into the pathophysiological relevance of neutrophil rTEM are at a developing stage, our previous findings suggested an association between neutrophil rTEM and distant organ inflammation (Woodfin et al., 2011). In line with this possibility, here we show that activation of LTB_4_–NE axis can drive a local inflammatory response to become a systemic multi-organ reaction, providing further evidence for an association between occurrence of neutrophil rTEM and development of secondary organ inflammation.

## Results

### Endogenously Generated LTB_4_ Accounts for Reduced Expression of Local EC JAM-C in Response to I-R

**Figure 1:**
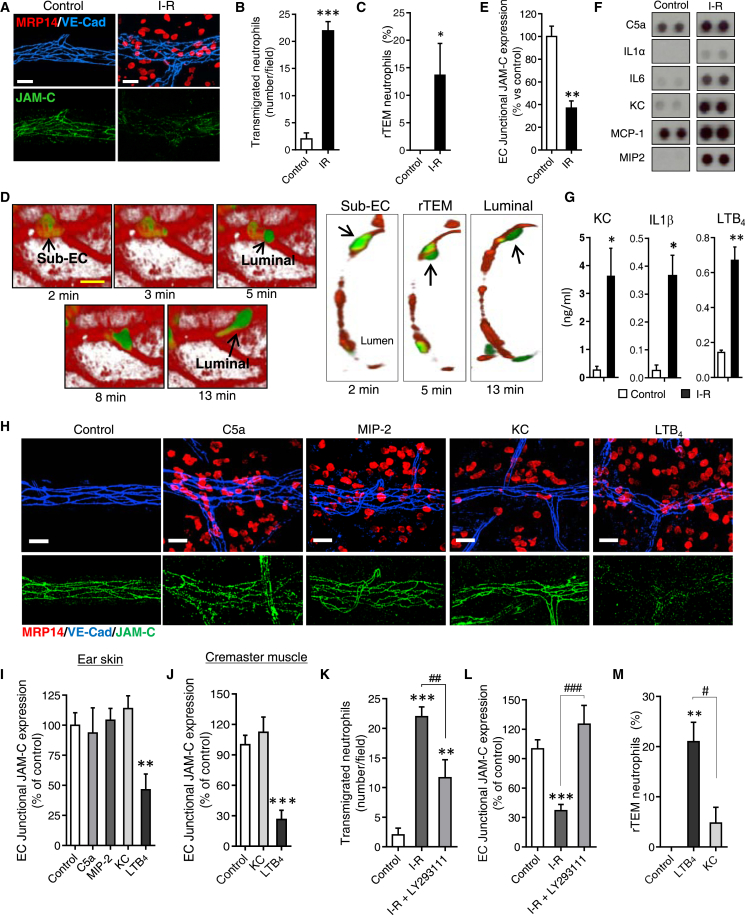
LTB_4_ Mediates I-R Induced Loss of EC JAM-C (A) Confocal images of mouse cremasteric venules in control or I-R stimulated tissues immunostained for MRP14 (neutrophils), VE-cadherin, and JAM-C. Images are representative of three independent experiments. (B) Quantification of transmigrated neutrophils (n = 3–6) and (C) frequency of neutrophil rTEM events (77–72 events, n = 3–7 mice) through mouse cremasteric venules of tissues subjected to I-R or sham operated, involving 14 independent experiments. (D) Time-lapse images of a movie (Movie S1) tracking the rTEM response of a GFP-labeled neutrophil (green) through a venule labeled with anti-PECAM-1 mAb (red) following cremaster I-R. On the left are video micrographs of a venular segment imaged from the luminal side that show a neutrophil (arrow head) in the sub-EC space (t = 2 min) that subsequently re-enters the vascular lumen (from 5 min onward) and eventually crawls back into the circulation (13 min). Right side panels show corresponding transverse sections of selected images. (E) Quantification of junctional EC JAM-C expression acquired from images as shown in (A) (n = 3–6). Images are representative of three independent experiments. (F and G) Inflammatory mediators in homogenated sham and I-R stimulated cremasters as measured by protein array (F) or ELISA (G) (n = 3). (H) Confocal images of mouse ear skin injected locally (4 hr, i.d.) with the indicated stimuli and immunostained for MRP14, VE-cadherin, and JAM-C. Images are representative of five independent experiments. (I and J) Quantification of junctional EC JAM-C expression in ears (I, from images as in H) or cremasters (J) of control tissues or tissues injected locally with the indicated stimuli, as analyzed by confocal microscopy (n = 4–6) from five independent experiments. (K and L) Quantification of transmigrated neutrophils (K) and junctional EC JAM-C expression (L), as analyzed by confocal microscopy, in sham operated controls or I-R injured cremasters in mice pretreated with vehicle or LY293111 (n = 3–5) involving three independent experiments. (M) Frequency of neutrophil rTEM events in cremaster muscles injected locally with saline, LTB_4_ or KC (n = 73-129 events, 3–9 mice) involving 15 independent experiments. Data indicate mean ± SEM or percentage of total events ± SEM (C and M). ^∗^p < 0.05, ^∗∗^p < 0.01 and ^∗∗∗^p < 0.001 as compared to controls and ^#^p < 0.05, ^##^p < 0.01, ^###^p < 0.001 as indicated by lines. Scale bars represent 20 μm (A and H) and 10 μm (D). See also Figure S1 and Movies S1 and S2.

The mechanism of EC JAM-C reduction was investigated in a murine model of cremaster muscle I-R (30 min ischemia, 2 hr reperfusion), an injury model that is amenable to rapid and high-resolution intravital microscopy (Woodfin et al., 2011). This sterile injury model is characterized by profound local neutrophil infiltration and is associated with increased frequency of neutrophil rTEM (Figures 1A–1D; Movie S1) (Woodfin et al., 2011). Elevated percentages of neutrophil rTEM were also observed in cremaster muscles injected with lipopolysaccharide (LPS) (26.4% of all TEM events, n = 6 mice, p = 0.040), identifying local endotoxemia as another inflammatory reaction in which neutrophil rTEM can occur. Since I-R-induced neutrophil rTEM was closely linked with loss of JAM-C from venular EC junctions (Figures 1A and 1E), to determine the mediator(s) that caused this effect, dissected control and I-R stimulated tissues were analyzed by protein array and ELISA. This approach identified numerous inflammatory mediators generated locally in response to I-R, including a number of cytokines (e.g., IL-1β, IL-1α, IL-6), chemokines (MIP-2, KC), C5a, and the lipid mediator LTB_4_ (Figure S1A; Figures 1F and 1G). To investigate the impact of these mediators on expression of EC JAM-C in vivo, we injected the stimuli locally into mouse ear skin or cremaster muscles and analyzed the tissues by immunofluorescent staining and confocal microscopy. Based on preliminary studies, a number of these stimuli were analyzed in detail. These included the neutrophil chemoattractants C5a, MIP-2, KC, and LTB_4_, mediators that were tested at doses that induced comparable neutrophil infiltration (Figure 1H). While intradermal C5a, MIP-2 and KC had no impact on EC JAM-C expression in skin, LTB_4_ reduced JAM-C expression on post-capillary venules (Figures 1H and 1I). LTB_4_, but not KC, also reduced the expression of EC JAM-C in cremasteric venules (Figure 1J). Locally administered LTB_4_ had no impact on protein expression or localization of other key EC junctional molecules such as PECAM-1, VE-cadherin, or JAM-A (Figure 1H; Figures S1B and S1C). The functional importance of endogenously generated LTB_4_ in the cremaster I-R model was confirmed using the LTB_4_ BLT1 receptor antagonist LY293111. Animals pre-treated systemically (intravenously, i.v.) with LY293111 showed significant reduction of local neutrophil infiltration (Figure 1K) but were totally protected from loss of EC JAM-C following I-R (Figure 1L). Collectively, these results show that in response to mouse cremasteric I-R, reduced expression of EC JAM-C is accounted for by endogenous LTB_4_ and that exogenous LTB_4_ is efficacious at causing loss of EC JAM-C. Furthermore, in agreement with our model that reduced functionality of EC JAM-C drives neutrophil rTEM, at doses that induced comparable neutrophil infiltration, local LTB_4_ but not KC promoted neutrophil rTEM (Figure 1M; Movie S2).

### LTB_4_-Induced Loss of EC JAM-C Is Neutrophil Dependent

**Figure 2:**
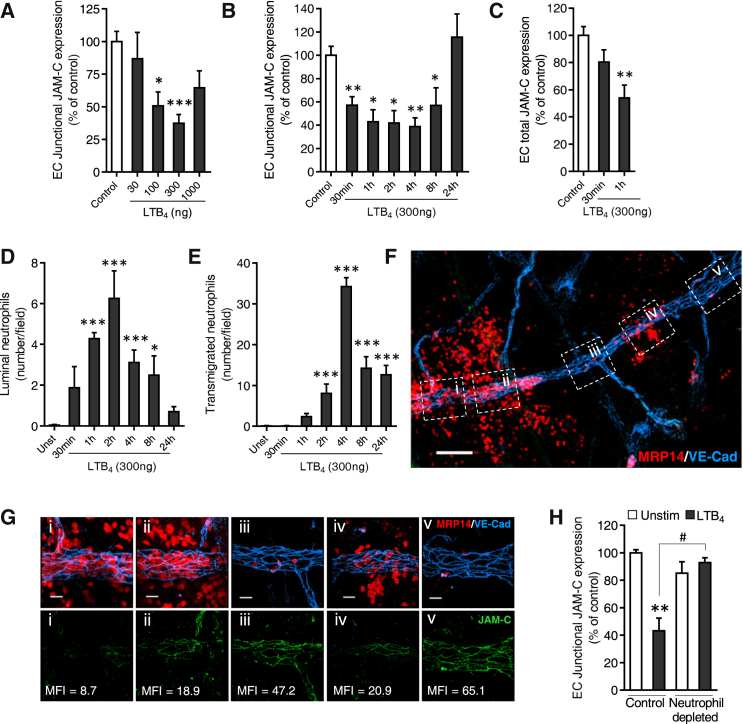
LTB_4_-Induced Loss of EC JAM-C Is Neutrophil Dependent (A) Dose response of LTB_4_-mediated reduced expression of EC JAM-C in ear skin of WT mice as analyzed by confocal microscopy (n = 4) from eight independent experiments. (B–E) Time course of LTB_4_-mediated reduced expression of junctional (B) or total (C) EC JAM-C, neutrophil adhesion (luminal) (D), and neutrophil transmigration (E) in ears, as analyzed by confocal microscopy (n = 4–8) involving 23 independent experiments. (F and G) Confocal images depicting “hot spots” of neutrophil (MRP14^+^) transmigration in microvessels (VE-cadherin) of LTB_4_-stimulated ears. (G) High magnification of the labeled (i–v) venular segments indicated in (F). The lower panels indicate the associated mean fluorescence intensities (MFI) of EC JAM-C junctional expression. (H) Quantification of junctional EC JAM-C expression in unstimulated or LTB_4_-stimulated ears in control and neutrophil-depleted mice (n = 3–7) involving four independent experiments. Data indicate mean ± SEM. ^∗^p < 0.05, ^∗∗^p < 0.01 and ^∗∗∗^p < 0.001, as compared to controls and ^#^p < 0.05 as indicated by lines. Scale bars represent 100 μm (F) and 20 μm (G). See also Figure S2.

Loss of EC JAM-C caused by locally administered LTB_4_ occurred in a dose- and time-dependent manner (Figures 2A and 2B). Significant reduction in expression of EC JAM-C at EC junctions was noted as early as 30 mins post local application of LTB_4_ (Figure 2B). This was sustained for up to 8 hr and returned to normal by 24 hr (Figure 2B). Of note, total expression of venular EC JAM-C (i.e., including junctional and cell body expression) was also significantly reduced post administration of LTB_4,_ but with a slight temporal delay as compared to junctional JAM-C (Figures 2B and 2C). These results suggest that EC JAM-C is initially re-localized from junctions to non-junctional regions (e.g., plasma membrane) following LTB_4_ stimulation, in agreement with our findings using the mouse cremaster I-R model (Scheiermann et al., 2009). This response might then be followed by total loss of the protein at later time points.

The time-course of LTB_4_-induced loss of venular JAM-C was directly aligned with time-course of LTB_4_-elicited neutrophil attachment to and migration through venular walls (Figures 2D and 2E). In addition, while JAM-C was expressed in all types of microvessels (capillaries > venules > arteries in ear skin and cremaster muscle; Figure S2), in LTB_4_-stimulated tissues, reduced expression of the molecule was selectively noted in post-capillary venules, the primary sites of neutrophil transmigration. Indeed, in tissues stimulated with locally administered LTB_4_, reduced expression of EC JAM-C was a feature of venular segments supporting intense neutrophil transmigration (“hot-spots”) (Figures 2F and 2G). Collectively, these results suggested a role for neutrophils in loss of EC JAM-C. Direct evidence for this was obtained through the use of mice depleted of their circulating neutrophils in which LTB_4_-injected tissues (skin) showed normal expression of venular JAM-C (Figure 2H), contrary to the reduced amounts noted in LTB_4_-stimulated control mice. These results indicate that loss of EC JAM-C following LTB_4_ stimulation is a neutrophil-dependent phenomenon and implied proteolytic cleavage of the protein as a possible mechanism.

### Neutrophil Elastase Cleaves EC JAM-C

**Figure 3:**
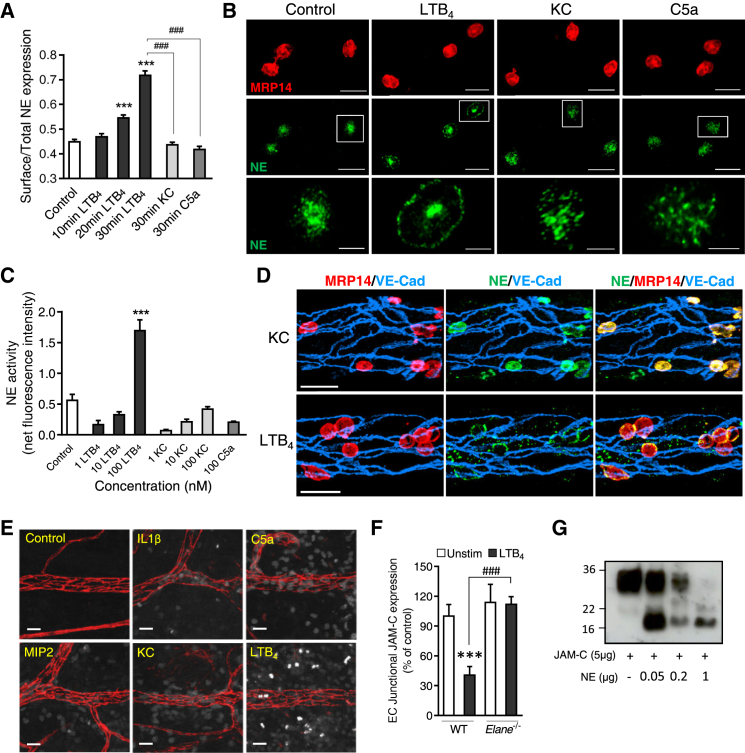
Neutrophil Elastase Mediates Cleavage of EC JAM-C (A–C) Effect of LTB_4_, as compared to other indicated stimuli, on cell surface NE protein expression (using an anti-NE mAb) (A and B, n = 32–102 cells) and NE activity (C, n = 23–166 cells) of BM neutrophils adherent to BSA-coated slides. Data are representative of three independent experiments. (D) Confocal images of stimulated cremaster muscles immunofluorescently stained for NE, neutrophils (MRP14), and VE-cadherin showing re-distribution of NE to the cell surface following 1 hr tissue stimulation with LTB_4_ but not KC (n = 2 mice per group). Images are representative of two independent experiments. (E) Confocal images showing NE activity (white) in ears injected intradermally with the indicated stimuli. Venules are shown in red (VE-cadherin) (n = 3 mice per group). Images are representative of two independent experiments. (F) Quantification of EC JAM-C expression in control and LTB_4_-stimulated ears of WT and *Elane*^−/−^ mice (n = 5–9) from four independent experiments. (G) Immunoblot against JAM-C shows the ability of purified NE to cleave recombinant JAM-C (representative of three independent experiments). Data indicate mean ± SEM. ^∗∗∗^p < 0.001, as compared to control and ^###^p < 0.001 as indicated by lines. Scale bars represent 5 μm (B, bottom panels) and 20 μm (B, upper panels; D and E). See also Figure S3.

Because LTB_4_ is a potent inducer of NE release and/or cell-surface expression (Rainger et al., 1998; Young et al., 2007), we considered that this serine protease might cleave EC JAM-C in response to LTB_4._ Initial in vitro studies using an anti-NE mAb and an NE-fluorescent activatable substrate (NE680FAST) illustrated the potent ability of LTB_4_ to rapidly mobilize intracellular stores of NE protein and to increase NE activity, respectively, to the neutrophil cell surface (Figures 3A–3C). Such effects were not noted with C5a or KC. Importantly, mobilization of neutrophil intracellular stores of NE protein and intense neutrophil-associated NE activity could also be observed in vivo in LTB_4_-stimulated tissues as compared to tissues stimulated by other inflammatory mediators (Figures 3D and 3E). The use of *Elane*^−/−^ (NE^−/−^) mice or the NE inhibitor GW311616A provided conclusive in vivo evidence for the involvement of NE in JAM-C cleavage (Figure 3F; Figure S3). Furthermore, purified NE cleaved recombinant soluble JAM-C in vitro, as shown by immunoblot (Figure 3G). Collectively, these results demonstrate that neutrophil-derived NE is responsible for loss of EC JAM-C as induced following rapid mobilization of the enzyme to the neutrophil cell surface by LTB_4_.

### Binding of NE to Mac-1 Supports Cleavage of JAM-C

**Figure 4:**
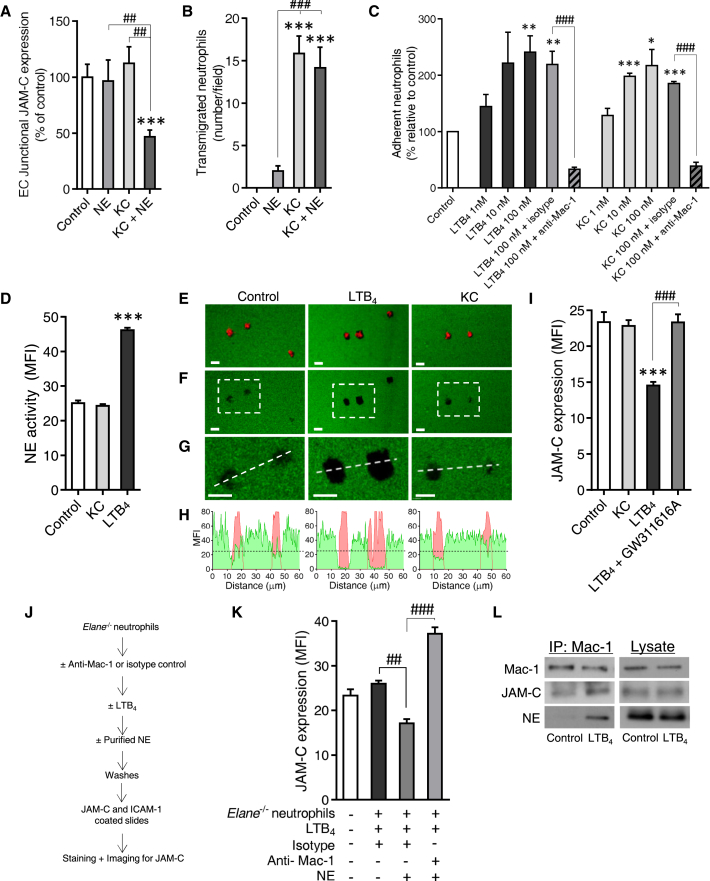
NE Bound to Mac-1 Mediates Cleavage of JAM-C (A and B) Quantification of EC JAM-C expression (A) and number of transmigrated neutrophils (B) in cremaster muscles locally injected (4 hr, i.s.) with the indicated stimuli (n = 3–6) involving four independent experiments. (C and D) Neutrophil adhesion to ICAM-1-coated slides (C) (n = 3 experiments) and NE activity (D) (n = 5 experiments) as induced by LTB_4_ and KC. (E–H) Unstimulated (control), LTB_4_- and KC-stimulated neutrophils (MRP-14; red) adherent to slides co-coated with ICAM-1 and JAM-C (green) (E) and associated regions of JAM-C loss (F). Bottom panels (G) show high-magnification images of the boxed regions in (F) with the associated linear intensity profiles (H) of the neutrophil (red) and JAM-C (green) channels along the white dotted lines (G). Black dotted lines in (H) indicate average expression of JAM-C at sites of neutrophil adhesion in control unstimulated samples. (I) Quantification of JAM-C at sites of neutrophil adhesion as illustrated in (E)–(H) (n = 4 experiments). (J and K) Protocol (J) used for quantification (K) of JAM-C expression post adhesion of LTB_4_-stimulated *Elane*^−/−^ neutrophils to JAM-C- and ICAM-1-coated slides (n = 3 experiments). (L) Co-immunoprecipitation and immunoblot analysis of untreated and LTB_4_-stimulated neutrophils (representative of two independent experiments). Data indicate mean ± SEM. ^∗^p < 0.05, ^∗∗^p < 0.01 and ^∗∗∗^p < 0.001 as compared to controls and ^##^p < 0.01, ^###^p < 0.001 as indicated by lines. Scale bars represent 10 μm.

We next sought to investigate the impact of exogenously administered NE on EC JAM-C in vivo. In contrast to the in vitro ability of NE to cleave JAM-C, local injection of NE into cremaster muscles failed to impact the expression of EC JAM-C (Figure 4A). This discrepancy was considered to be due to an essential need for neutrophils in vivo, because exogenous NE failed to significantly induce neutrophil transmigration (Figure 4B). Specifically, we hypothesized that neutrophils might be required to present NE to JAM-C on ECs in order for NE to cleave this adhesion molecule. To explore this possibility, we co-injected NE with KC, a chemokine that induced significant neutrophil infiltration but at the dose tested had no impact on EC JAM-C expression (Figures 4A and 4B). Cremaster muscles co-injected locally with NE and KC showed similar neutrophil infiltration to that noted with KC alone (Figure 4B). However, in contrast to tissues injected with KC or NE alone, tissues stimulated with NE+KC exhibited a significant reduction in EC JAM-C expression (Figure 4A). Because NE has previously been reported to bind to the neutrophil integrin Mac-1 (Cai and Wright, 1996) and Mac-1 is a ligand for JAM-C (Santoso et al., 2002), we considered that the underlying reason for the efficacy of LTB_4_ to induce cleavage of JAM-C might be due to the ability of the lipid to be both an effective activator of Mac-1 and inducer of NE release. In support of this, mouse neutrophils stimulated with LTB_4_ or KC adhered to ICAM-1-coated slides in a Mac-1-dependent manner (Figure 4C), demonstrating activation of the integrin by these neutrophil chemoattractants. Under these in vitro conditions, however, at concentrations that caused comparable adhesion to ICAM-1-coated slides, only LTB_4_ was capable of eliciting release of NE (Figure 4D). The functional implication of this in relation to JAM-C cleavage was investigated in an assay in which the interaction of neutrophils with JAM-C and ICAM-1-coated slides was analyzed by fluorescent microscopy and JAM-C cleavage was quantified through immunofluorescence detection of JAM-C (Figures 4E–4H). Stimulation of cells with LTB_4_ or KC again induced comparable neutrophil adhesion to the coated slides (as found with ICAM-1-coated slides), but only LTB_4_ stimulation significantly increased cleavage of JAM-C as compared to unstimulated control cells (Figures 4E–4I). The latter response was NE dependent, as assessed using an NE inhibitor (GW311616A) (Figure 4I). This assay was next extended to explore the possibility that direct interaction of Mac-1 with NE is necessary for cleavage of JAM-C. For this purpose, *Elane*^−/−^ neutrophils were pre-treated with or without anti-Mac-1 mAb followed by LTB_4_ stimulation in the presence of exogenous NE (Figure 4J). The resulting cell samples were then washed and placed onto JAM-C- and ICAM-1-coated slides, and after 30 min the slides were fixed and stained for JAM-C expression. In this model, LTB_4_-stimulated *Elane*^−/−^ cells supplemented with exogenous NE led to JAM-C cleavage, and this response was totally inhibited under conditions of Mac-1 blockade (Figure 4K). Finally, co-immunoprecipitation experiments showed that Mac-1 from LTB_4_-stimulated (but not control unstimulated) mouse neutrophils binds to both NE and JAM-C (Figure 4L). Collectively, these results further illustrated the importance of NE in JAM-C cleavage and demonstrated that NE bound to neutrophil Mac-1 supported this response.

### Local LTB_4_-NE Axis Promotes Neutrophil rTEM

**Figure 5:**
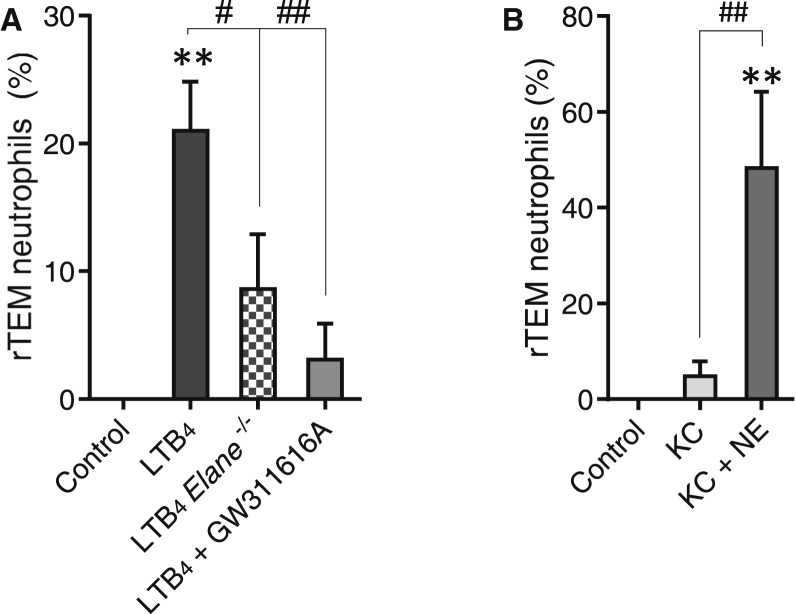
LTB_4_ and NE Promote Neutrophil rTEM Frequency of neutrophil rTEM events through cremasteric venules of tissues from WT mice injected locally with saline (control) or LTB_4_ with or without pre-treatment with GW311616A and in Elane^−/−^ mice injected with LTB_4_ (A) and in tissues injected locally with KC or KC+NE (B) (19–217 events, 3–9 mice) involving 22 independent experiments. Data indicate percentage of total events ± SEM. ^∗∗^p < 0.01 as compared to controls and ^#^p < 0.05 and ^##^p < 0.01 as indicated by lines.

Because LTB_4_ and NE were identified as key molecular players in inducing loss of EC JAM-C and we have previously shown that reduced expression of EC JAM-C drives neutrophil rTEM (Woodfin et al., 2011), we next explored the role of the LTB_4_-NE axis in induction of neutrophil rTEM. LTB_4_-induced neutrophil rTEM was significantly suppressed in *Elane*^−/−^ mice and in wild-type (WT) mice pre-treated with an NE inhibitor (Figure 5A). In addition, because KC, a mediator that did not impact the expression of EC JAM-C (Figure 1H–1J), also failed to elicit a notable frequency of neutrophil rTEM (Figure 1M), the effect of the chemokine when co-injected with NE into the cremaster muscle (a reaction that caused local loss of EC JAM-C; Figure 4A) was tested. This reaction led to profound frequency of neutrophil rTEM as compared to responses detected in mice injected with KC alone (Figure 5B). Collectively, the results demonstrate that locally activated LTB_4_-NE axis is highly effective at promoting neutrophil rTEM.

### Activation of LTB_4_-NE Axis Drives a Local Inflammatory Response to Become Systemic

**Figure 6:**
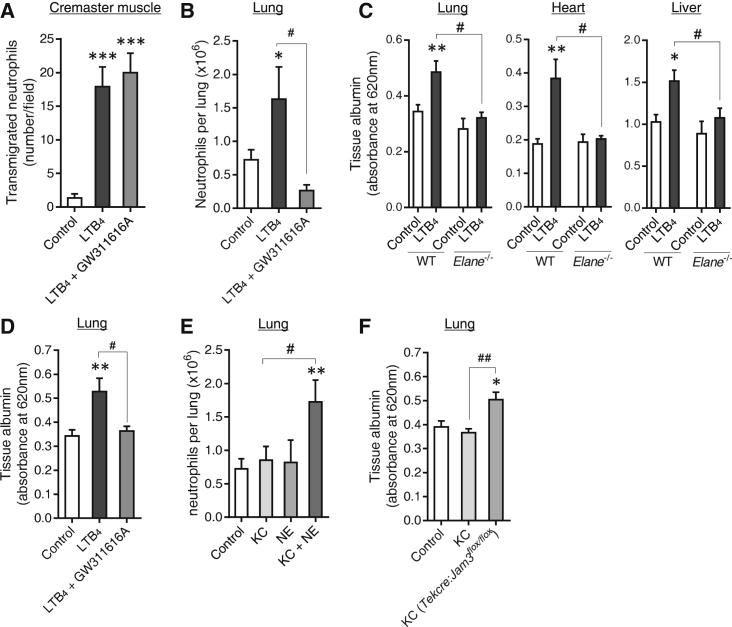
LTB_4_ and NE Promote Distant Organ Damage (A) Quantification of transmigrated neutrophils into mouse cremaster muscles in unstimulated tissues (control) or 4 hr post local injection of LTB_4_ with or without pretreatment (24 hr) of mice with GW311616A (n = 4–7) from six independent experiments. (B) Quantification of lung neutrophil numbers (n = 4–15) following cremaster stimulation (as in A) involving 15 independent experiments. (C) Quantification of lung, heart, and liver albumin content (plasma extravasation), as a marker of tissue damage, following local (cremaster) injection of LTB_4_ (4 hr) as compared to control unstimulated tissues in WT and *Elane*^−/−^ mice (n = 4–24) involving 12 independent experiments. (D) Lung albumin content in unstimulated tissues (control) or post local (cremaster) injection of LTB_4_ with or without pretreatment (24 hr) of mice with GW311616A (n = 4–24) involving 16 independent experiments. (E and F) Lung neutrophil numbers (E) (n = 6–15) and tissue albumin content (F) (n = 3–5) following local injection of KC, NE, or KC+NE in cremaster muscles of WT or *Tekcre;Jam-3*^flox/flox^ mice involving 21 independent experiments. Data indicate mean ± SEM. ^∗^p < 0.05, ^∗∗^p < 0.01 and ^∗∗∗^p < 0.001 as compared to controls and ^#^p < 0.05 and ^##^p < 0.01 as indicated by lines. See also Figure S4.

We have previously found an association between loss of EC JAM-C expression and/or functionality, neutrophil rTEM, and distant organ (lung) inflammation (Woodfin et al., 2011). Having identified LTB_4_ as an effective stimulus that triggers NE-mediated loss of EC JAM-C and promoted neutrophil rTEM, we investigated a possible causal relationship between this cascade of events and secondary organ inflammation. In addition to stimulating local neutrophil infiltration (Figure 6A), LTB_4_ injected into the mouse cremaster muscle elicited neutrophil accumulation into lungs (Figure 6B) and tissue damage (plasma protein leakage) in multiple organs, e.g., lung, heart, and liver (Figure 6C). Time-course studies illustrated that, after injection of LTB_4_ into the mouse cremaster muscle, lung injury peaked at 4 hr and returned to normal by 24 hr (Figure S4A). A similar transient profile of lung injury was found in other tissues such as the heart (Figure S4B). Similarly to the cremaster muscle, intradermal injection of LTB_4_ into the ear skin promoted lung neutrophil accumulation (Figure S4C) and remote organ damage (Figure S4D). These effects were attenuated in *Elane*^−/−^ mice or in WT mice pre-treated with the NE inhibitor GW311616A (Figures 6B–6D; Figure S4D), without affecting local LTB_4_-induced neutrophil recruitment (Figure 6A). Of note, *Elane*^−/−^ mice and WT mice pretreated with GW311616A exhibited normal neutrophil migration into lungs in response to intranasal instillation of LTB_4_ (Figure S4E), indicating that NE blockade does not directly suppress neutrophil infiltration into lungs. Together these results show that LTB_4_, when administered into a primary tissue site, can promote secondary organ damage in an NE-dependent manner. Importantly, we also noted increased remote organ (lung and heart) damage following cremaster I-R injury, a response that was inhibited in *Elane*^−/−^ mice (Figure S4F).

Because NE was found to be essential in regulating LTB_4_-induced local EC JAM-C expression (Figure 3F; Figure S3), a potential link between local loss of EC JAM-C and secondary organ inflammation was further investigated. For this purpose, we analyzed the effects of locally administered KC, a neutrophil chemoattractant that did not cause loss of EC JAM-C (Figures 1H–1J). When applied locally to the cremaster muscle at a dose that elicited significant local neutrophil infiltration (Figure 4B), KC did not cause neutrophil recruitment into lungs (Figure 6E). In contrast, while the combined injection of KC and NE into the cremaster muscle induced comparable neutrophil infiltration to that observed with KC alone (Figure 4B), KC+NE led to significant neutrophil accumulation into lungs, as compared to responses detected in mice treated with KC or NE alone (Figure 6E). Because we have previously shown that KC+NE can cause local loss of EC JAM-C and promote neutrophil rTEM (Figure 4A and 5B), these results provide strong evidence to suggest that local regulation of EC JAM-C is instrumental in promoting distant organ damage. In support of this, we noted that KC, when injected into the cremaster muscle of mice with specific deletion of EC JAM-C (*Tekcre;Jam-3*^flox/flox^), caused significant lung edema as compared to responses observed in littermate controls (Figure 6F). Collectively, through loss-of-function and gain-of-function studies, these results demonstrate that inflammatory reactions that can mediate NE-induced local loss of EC JAM-C can promote development of secondary multi-organ inflammation.

### Soluble JAM-C Is Detected in Plasma of Locally Inflamed Mice and in Trauma Patients

**Figure 7:**
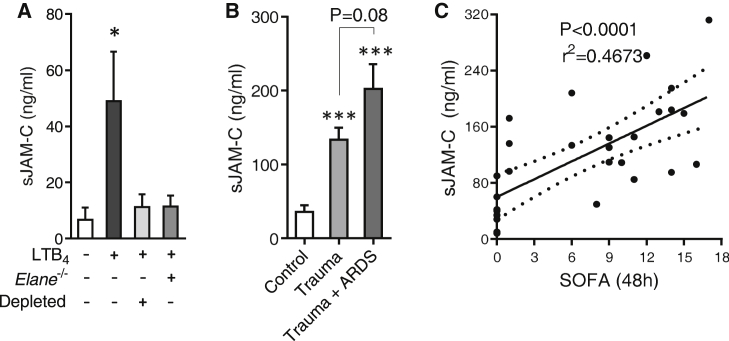
Locally Inflamed Mice and Trauma Patients Exhibit Increased Plasma Content of Soluble JAM-C (A) Plasma sJAM-C in WT, *Elane*^−/−^, and neutrophil depleted mice under basal conditions (control) and following intradermal injection (ear, i.d. 4 hr) of LTB_4_ (n = 4–16) involving 11 independent experiments. (B) Plasma content of sJAM-C in healthy control volunteers and trauma patients with or without ARDS (n = 8–10). (C) Correlation of plasma sJAM-C with SOFA scores in individuals from (B). Data indicate mean ± SEM. ^∗^p < 0.05 and ^∗∗∗^p < 0.001 as compared to controls. Additional statistical analyses are indicated.

As a consequence of local proteolytic cleavage by NE, we speculated that soluble JAM-C (sJAM-C) might be detectable in plasma of mice after tissue stimulation. The low circulating sJAM-C detected in control mice was significantly increased following intradermal injection of LTB_4_ (Figure 7A), a parameter that inversely correlated with the loss of venular EC JAM-C (p = 0.0041). This response was abrogated in *Elane*^−/−^ mice and in mice depleted of their circulating neutrophils (Figure 7A), illustrating that as found with local loss of venular EC JAM-C, presence of sJAM-C in plasma is both neutrophil- and NE-dependent. Importantly, elevated sJAM-C was also found in plasma samples from trauma patients as compared to control volunteers (Figure 7B). Patients who developed acute respiratory distress syndrome (ARDS) exhibited higher amounts (>50%) of plasma sJAM-C on admission than those who maintained normal lung function (Figure 7B). Furthermore, there was a significant correlation between on admission plasma content of sJAM-C and the subsequent severity of multi-organ failure (MOF) as assessed by their Sequential Organ Failure Assessment (SOFA) scores at 48 hr after injury (Figure 7C). Thus, in conjunction with our previous findings, there is an association between increased amounts of sJAM-C in plasma and distant organ inflammation following local LTB_4_ in mice and importantly post tissue injury in humans.

## Discussion

Here we investigated the mechanism through which neutrophil rTEM is triggered in vivo. The results identified the lipid neutrophil chemoattractant LTB_4_ as a key mediator capable of promoting proteolytic cleavage of EC JAM-C by NE, a response that drives neutrophil rTEM. The activation of this cascade of events was also associated with dissemination of systemic inflammation, suggesting that targeting LTB_4_-NE axis might be an efficacious means of suppressing secondary organ damage following local inflammation or injury.

While neutrophils play a key role in mounting an early innate immune response and have also been intimately associated with the development of acute inflammatory disorders, there is now ample evidence for a broader role for neutrophils in inflammation and immunity (Mantovani et al., 2011; Mócsai, 2013; Mayadas et al., 2014). Most notably, neutrophils are known to interact with components of the adaptive immune response and have been implicated in the pathogenesis of numerous chronic inflammatory conditions (Mantovani et al., 2011; Mócsai, 2013; Mayadas et al., 2014). Furthermore, the traditional view of neutrophils exhibiting a short half-life (i.e., < several hours) has been challenged (Tak et al., 2013), and there is evidence for the existence of neutrophil “subsets” exhibiting a wide range of phenotypes in different physiological and pathological conditions (Beyrau et al., 2012; Kolaczkowska and Kubes, 2013). In addition, while conventionally neutrophils have been considered as cells that move in a one-way direction, there is now evidence for the ability of neutrophils to breach ECs in an abluminal-to-luminal direction, i.e., exhibit reverse TEM (Buckley et al., 2006; Mathias et al., 2006; Woodfin et al., 2011), though our understanding of the prevalence, regulation, and implications of this response are at an early stage.

Through the application of confocal IVM to analysis of inflamed mouse cremaster muscles, we recently reported significant frequency of neutrophil rTEM following I-R (Woodfin et al., 2011). Mechanistically, rTEM was associated with and indeed promoted by reduced expression and/or function of EC JAM-C (Woodfin et al., 2011). To gain a better understanding of the inflammatory trigger(s) that promote neutrophil rTEM, we sought to identify the mechanism(s) through which EC JAM-C is lost. Analysis of mouse cremaster muscles subjected to I-R detected an array of endogenously generated mediators, in line with the network of mediators identified in other models of I-R (Iadecola and Anrather, 2011; Smith et al., 2012). Among these, LTB_4_ was identified as a mediator that effectively reduced expression of EC JAM-C in vivo, and through the use of a specific BLT1 receptor antagonist, endogenous LTB_4_ was found to be totally accountable for loss of EC JAM-C expression in response to I-R. In agreement with investigations of other disease models (Souza et al., 2000; Kim et al., 2006; Chou et al., 2010), LTB_4_ also contributed to local neutrophil infiltration as elicited by I-R. An important cellular source of this LTB_4_ is likely to be the neutrophils themselves (Chen et al., 2006; Afonso et al., 2012; Lämmermann et al., 2013). The potent ability of locally administered LTB_4_ to cause loss of EC JAM-C was associated with the ability of this mediator to induce neutrophil rTEM. These results are directly in line with our previous findings (Woodfin et al., 2011) and the overall hypothesis that loss of EC JAM-C is instrumental in mediating neutrophil rTEM.

The effect of LTB_4_ was selective to JAM-C in that it had no impact on expression of other key EC adhesion molecules. We have previously reported that in response to I-R, EC JAM-C is re-distributed from junctions and intracellular cytoplasmic vesicles toward non-junctional plasma membrane (Scheiermann et al., 2009). Similarly, our present findings suggest an initial redistribution of the molecule away from EC junctions following LTB_4_ stimulation, a response that could support adhesion of neutrophils to the endothelium (Scheiermann et al., 2009). Reduction of both junctional and total JAM-C expression at later time points suggested that the protein was also cleaved from ECs in this reaction, and multiple lines of evidence indicated that this was a neutrophil-dependent response. This included the following: (1) The response occurred largely in post-capillary venules, the primary site of neutrophil transmigration, (2) the time-course of the response was directly aligned with the time-course of neutrophil adhesion and transmigration, (3) EC JAM-C loss was most notable at sites of intense neutrophil transmigration (“hot-spots”), and finally, (4) LTB_4_-induced loss of EC JAM-C was abolished in neutrophil-depleted mice. In investigating the mechanism through which neutrophils caused reduced expression of EC JAM-C, we focused our attention on the possible role of NE. This serine protease is expressed within neutrophil azurophil granules and exhibits a broad substrate specificity, including numerous cell-surface receptors and adhesion molecules (Pham, 2006). Because LTB_4_ is highly efficacious at inducing NE degranulation from mouse neutrophils in vitro (Young et al., 2007), we hypothesized that NE might mediate neutrophil-dependent loss of EC JAM-C. In support of this, LTB_4_ showed high efficacy in promoting rapid NE release and/or cell-surface expression and activity (as mobilized from intracellular stores), both in vitro and in vivo, as compared to other neutrophil chemoattractants. Furthermore, we have recently demonstrated that local LTB_4_ can rapidly activate neutrophils within the vascular lumen when the neutrophils are in close apposition to the venular wall and junctions (Finsterbusch et al., 2014). Direct evidence for the role of NE in mediating LTB_4_-induced loss of venular JAM-C was provided with the use of NE-deficient mice or mice pre-treated with an NE inhibitor. Together, our findings identify the potent ability of LTB_4_ to induce mobilization of NE to the neutrophil cell surface as a key factor in the ability of this lipid mediator to cause loss of JAM-C at sites of inflammation.

Activation of the neutrophil BLT1 receptor is efficiently linked to signaling pathways that trigger degranulation of azurophil granules containing NE (Rainger et al., 1998). This could be of physiological benefit when LTB_4_ is generated and localized to the interstitial tissue. Here it can support clearance of pathogens or act as a signal-relay molecule for neutrophil chemotaxis or neutrophil swarming to sites of injury, responses that have been linked to NE and LTB_4_, respectively (Belaaouaj et al., 1998; Afonso et al., 2012; Lämmermann et al., 2013). In contrast, under pathological conditions, such as that encountered following I-R injury, excessive generation of LTB_4_ might activate neutrophils within the vascular lumen, leading to inappropriate and damaging release of NE at the vessel wall. Based on our findings and proposed model, the latter could lead to cleavage of EC JAM-C and development of rTEM. To achieve this effect, we hypothesized that following its release from azurophil granules, neutrophil surface-bound NE is presented to EC JAM-C during TEM. The concept that serine proteases remain cell bound after fusion of granules with the plasma membrane is well recognized and is considered as a means through which the enzymes are protected from endogenous interstitial protease inhibitors (Owen and Campbell, 1999). What is less clear, however, is the mechanism through which serine proteases bind to the neutrophil cell surface. In this context while charge-dependent mechanisms have been proposed (Campbell and Owen, 2007), NE has also been shown to interact with neutrophil Mac-1 (Cai and Wright, 1996). Building on these findings and the fact that Mac-1 has been identified as a ligand for JAM-C (Santoso et al., 2002), we extended our hypothesis to a potential role for an LTB_4_-NE-Mac-1 axis in cleavage of JAM-C. Using a number of in vitro models, our findings demonstrated that although both LTB_4_ and KC could effectively induce Mac-1 activation, the efficacy of LTB_4_ to induce JAM-C cleavage resides in the capacity of this mediator to induce NE expression to the neutrophil cell surface. Taking advantage of NE deficient neutrophils, evidence was obtained for the ability of NE to bind to activated Mac-1 upon LTB_4_ stimulation, an interaction that facilitated JAM-C cleavage. Importantly, rTEM caused by LTB_4_ was NE-dependent, and co-injection of exogenous NE with KC (at doses that individually did not cause neutrophil rTEM) induced profound rTEM, demonstrating that the activation of the LTB_4_-NE axis (through loss-of-function or gain-of-function studies) can lead to loss of EC JAM-C and promotion of neutrophil rTEM. Collectively, these findings provide support for the overall hypothesis that NE is presented to EC JAM-C via activated Mac-1, the latter acting as a molecular “bridge” between NE and JAM-C. This provides a mechanism through which JAM-C is cleaved in a selective manner, thus promoting neutrophil rTEM.

At present, the functional implication of neutrophils undergoing reverse TEM is unclear. It is potentially possible that this phenomenon might have a physiological role such as dampening down a local inflammatory response (Mathias et al., 2006; Yoo and Huttenlocher, 2011) and/or reflect a role for neutrophils as cellular sentinels or sirens of inflammation. Alternatively, neutrophil rTEM might play a pathological role such as contributing to turning a local acute inflammatory response into a systemic phenomenon (Woodfin et al., 2011). Although further studies are required, the results of the present study support the latter. Most notably, local I-R injury or local administration of LTB_4_ (cremaster muscle or ear skin) caused remote organ damage. Furthermore, all pharmacological or genetic interventions that suppressed or enhanced neutrophil rTEM (e.g., *Elane*^−/−^ mice and NE+KC), correspondingly regulated second organ inflammation. Because neutrophils that have undergone rTEM have been shown to exhibit a pro-inflammatory state (Buckley et al., 2006; Woodfin et al., 2011), the present findings support the paradigm that reverse TEM results in re-entry of a small subset of activated neutrophils into the blood circulation that can contribute to turning a local inflammatory response into a systemic phenomenon. The findings further indicated that NE inhibition had no impact on local neutrophil infiltration into the cremaster muscle, suggesting that although NE is not essential in mediating local neutrophil infiltration, excessive and/or inappropriately generated local NE can promote neutrophil rTEM and remote organ damage.

As a consequence of EC JAM-C cleavage by NE, increased amounts of circulating sJAM-C were found in mice locally stimulated with LTB_4_. Elevated concentration of sJAM-C was also found in plasma from trauma patients as compared to healthy controls, a parameter that further increased in patients that developed acute respiratory distress syndrome (ARDS) after admission. ARDS is characterized by severe respiratory failure, and patients with ARDS exhibit ∼40% mortality rate (Rubenfeld et al., 2005; Wheeler and Bernard, 2007). Pathophysiology of ARDS is characterized by increased lung permeability and neutrophil infiltration and is typically associated with sepsis and multiple organ failure (MOF) (Matthay and Zemans, 2011). Our findings, together with the reported elevated plasma content of LTB_4_, as well as NE activity, found in trauma patients that progress to ARDS (Donnelly et al., 1995; Auner et al., 2012), suggest that similar mechanisms (i.e., LTB_4_-dependent NE cleavage of EC JAM-C) could be taking place following trauma. By extension, the results suggest that blocking JAM-C cleavage by inhibition of NE could be a useful preventative strategy in trauma patients at risk of developing ARDS and MOF. Importantly, NE inhibitors are currently in use in Japan in patients with ARDS associated with systemic inflammatory response syndrome (SIRS), as well as for reducing surgery-induced pulmonary inflammation (Fujii et al., 2010; Aikawa et al., 2011). Together, the present study provides previously unknown mechanistic insights into how NE inhibitors might prevent secondary ARDS. Furthermore, because increased plasma content of sJAM-C was associated with trauma-induced organ failure and also elevated in serum or synovial fluid from rheumatoid arthritis, psoriatic arthritis, osteoarthritis, and systemic sclerosis patients (Rabquer et al., 2010; Manetti et al., 2013), we propose that sJAM-C might be a useful vascular-derived biomarker for assessing the extent of a systemic inflammatory response.

In summary, we have identified a role for local LTB_4_ and NE as regulators of neutrophil rTEM. Furthermore, although at present the precise functional implications of neutrophils undergoing rTEM remain unclear, the results of this study provide further evidence for this phenomenon being associated with dissemination of inflammation.

## Experimental Procedures

### Animals

*Lyz2-EGFP-ki* mice (exhibiting green fluorescent neutrophils), *Elane*^−/−^ mice, *Elane*^−/−^*-Lyz2-EGFP-ki* mice, EC JAM-C^−/−^ mice (*Tekcre;Jam-3*^flox/flox^) and their control littermates (*Jam-3*^flox/flox^), and wild-type C57BL/6 mice were used. All animal experiments were conducted in accordance with the UK Home Office legislations.

### Patients

Blood from trauma patients (sampled less than 2 hr after injury) or healthy donor controls was collected in buffered sodium citrate.

### Induction of Inflammatory Reactions and Pre-treatments

Inflammatory stimuli such as LTB_4_, LPS, CXCL1 (KC), CXCL2 (MIP-2), C5a, or vehicle control were injected in the ears or the cremaster muscles of anesthetized mice. Some mice were pre-treated (24 hr, orally) with the NE inhibitor GW311616A. Cremaster I-R injury (30 min ischemia followed by 2 hr reperfusion) was induced as previously detailed (Scheiermann et al., 2009). In some experiments, the LTB_4_ receptor antagonist LY293111 was administered i.v. 15 min prior to I-R.

### Whole-Mount Tissue Immunofluorescence Staining

Ears or cremaster muscles were immunostained before being mounted on slides and analyzed by confocal microscopy.

### Confocal Microscopy

Immunofluorescently stained cells and whole-mounted tissues were imaged by confocal microscopy and analyzed by IMARIS or ImageJ software.

### Quantification of Plasma Soluble JAM-C

sJAM-C plasma content was analyzed by ELISA.

### Cytokine and Chemokine Expression Profile

The profile of different inflammatory mediators in mouse cremasters was analyzed using the Mouse Cytokine Array Panel A Array Kit or ELISA kits.

### In Vitro Digestion of JAM-C

Purified recombinant murine JAM-C was incubated with purified NE for 1 hr at 37°. Samples were then resolved in a SDS-PAGE gel and visualized by Western blot.

### Co-immunoprecipitation

Bone marrow (BM)-derived neutrophils were purified and stimulated with LTB_4_ or left unstimulated. Samples were then lysed, incubated overnight with an anti-Mac-1 mAb, and immunoprecipitated. JAM-C and NE protein content was then analyzed by Western blot.

### Immunoblot

Samples were resolved on SDS-PAGE gels and electrotransferred onto PVDF membranes. These were then blocked, incubated with primary and HRP-conjugated secondary antibodies, and developed using a Chemoluminescent Substrate.

### In Vitro Neutrophil Adhesion Assay

16-well glass chamber slides were coated with recombinant human ICAM-1 and blocked with 10% BSA. BM Neutrophils (5 × 10^4^/well) were added to the chambers and were either left untreated or stimulated with LTB_4_. In some experiments, neutrophils were pre-incubated with an anti-Mac-1 mAb. The number of adherent cells was quantified by phase contrast microscopy.

### Imaging of JAM-C Cleavage In Vitro

BM neutrophils seeded in 16-well glass chamber slides coated with recombinant ICAM-1 and JAM-C were left untreated or stimulated with LTB_4_, LTB_4_+ GW311616A or KC for 30 min. Slides were then fixed, immunostained with antibodies against JAM-C and MRP-14, and analyzed by confocal microscopy.

### In Vivo NE Enzymatic Activity Assay

NE680FAST was injected i.v. and left to circulate for 15 min before induction of inflammation. At the end of the experiment tissues were collected, immunofluorescently stained, and analyzed by confocal microscopy.

### In Vitro NE Enzymatic Activity Assay

BM neutrophils were seeded on ICAM-1 and JAM-C coated slides and left untreated or stimulated for 30 min with KC or LTB_4_ in the presence of NE680FAST. Slides were then washed, fixed, immunostained, and analyzed by confocal microscopy.

### Confocal Intravital Microscopy of Mouse Cremaster Muscles

*Lyz2*-*EGFP-ki* mice were injected (i.s.) with Alexa-555-PECAM-1 to stain EC junctions of the cremaster microvasculature. After cremaster exteriorization, postcapillary venules were selected for in vivo analysis of leukocyte-vessel wall interactions.

### Analysis of Lung Neutrophil Infiltration

Following perfusion of pulmonary vasculature, lungs were excised and digested. The resulting cells were stained and the number of neutrophils analyzed by flow cytometry.

### Flow Cytometry

Samples were immunostained with fluorescently conjugated antibodies. Following red blood cell lysis, samples were run on a flow cytometer and analyzed using Flowjo software (TreeStar).

### Measurement of Tissue Plasma Extravasation

Evans blue solution was injected i.v. and allowed to circulate for 10 min before mice were killed. Following vascular wash-out, tissues were collected and the accumulated Evans blue (tissue albumin) was quantified as a measure of plasma leakage.

### Statistics

Data analysis was performed using the statistical software GraphPad Prism 4. Results are expressed as mean ± SEM. Statistical significances were assessed by Student’s t test, chi-square test, one-way ANOVA with Student-Newman-Keuls multiple comparison test, or two-way ANOVA with Bonferroni post hoc test as appropriate. Correlations were quantified by Pearson’s correlation coefficient. p < 0.05 were considered significant.

## Author Contributions

B.C. designed and performed most experiments, analyzed and interpreted data, and contributed to the writing of the manuscript; J.V.B. performed and analyzed IVM experiments; M.B. performed and analyzed in vitro neutrophil adhesion assays; A.W. provided technical assistance for the IVM experiments; C.O. performed the JAM-C ELISA assays; C.R. and K.B. provided clinical samples and patient SOFA scores; T.C. and B.A.I. provided reagents and contributed intellectually; and S.N. provided overall project supervision, contributed to the design of the experiments, and wrote the manuscript.

## Supplemental Information

Document S1. Figures S1–S4 and Supplemental Experimental Procedures

Movie S1. Neutrophil Reverse TEM as Induced by I-R Injury, Related to Figure 1

Movie S2. Neutrophil Reverse TEM as Induced by Topical LTB_4_, Related to Figure 1

Document S2. Article plus Supplemental Information
