# Supplementary material for: Leukotriene B4-Neutrophil Elastase Axis Drives Neutrophil Reverse Transendothelial Cell Migration In Vivo
Source: Immunity. 2015 Jun 16;42(6):1075–86. doi: 10.1016/j.immuni.2015.05.010 (PMC4504024; doi:10.1016/j.immuni.2015.05.010)
Supplement: Document S1. Figures S1–S4 and Supplemental Experimental Procedures [file mmc1.pdf]

Immunity

Supplemental Information

Leukotriene B_4-Neutrophil Elastase Axis Drives Neutrophil Reverse Transendothelial Cell Migration In Vivo

Bartomeu Colom, Jennifer V. Bodkin, Martina Beyrau, Abigail Woodfin, Christiane Ody, Claire Rourke, Triantafyllos Chavakis, Karim Brohi, Beat A. Imhof, and Sussan Nourshargh

SUPPLEMENTAL INFORMATION

SUPPLEMENTAL FIGURES

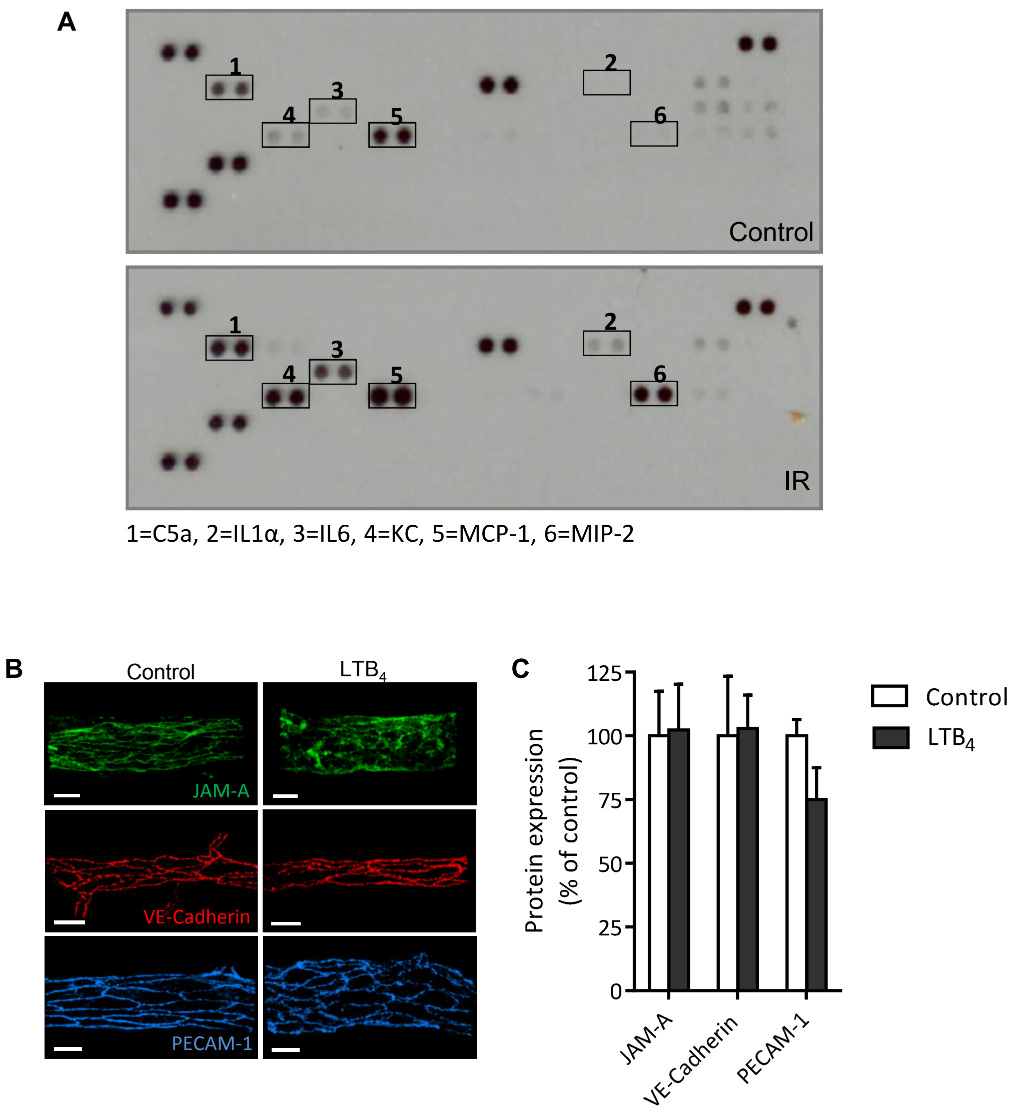

Figure S1

Figure S1. Related to Figure 1. Characterization of the inflammatory mediator generation and expression profile of EC adhesion molecules in response to cremaster muscle ischemia-reperfusion injury or intradermal injection of LTB_4, respectively. (A) The inflammatory mediator expression profile in sham (control) and I-R stimulated cremaster muscles was measured in tissue homogenates (pooled samples from 3 mice per group) using a Mouse Cytokine Array Panel A kit. Densitometry from the blots was analyzed with ImageJ software. Images are representative of 2 independent experiments. (B-C) Locally administered LTB_4 does not impact the expression profile of EC JAM-A, VE-cadherin or PECAM-1. Images (B) and quantification of protein expression levels (C) of the indicated adhesion molecules at EC junctions of mouse ear dermal post-capillary venules in control (B, left panels) and in stimulated tissues (4h i.d. LTB_4) (B, right panels), as analyzed by immunofluorescent staining and confocal microscopy (n=4 mice) from 3 independent experiments. Data are percentage change in mean fluorescent intensity (MFI) of signals acquired from stimulated samples relative to controls and presented as mean ± SEM. Scale bars, 20μm.

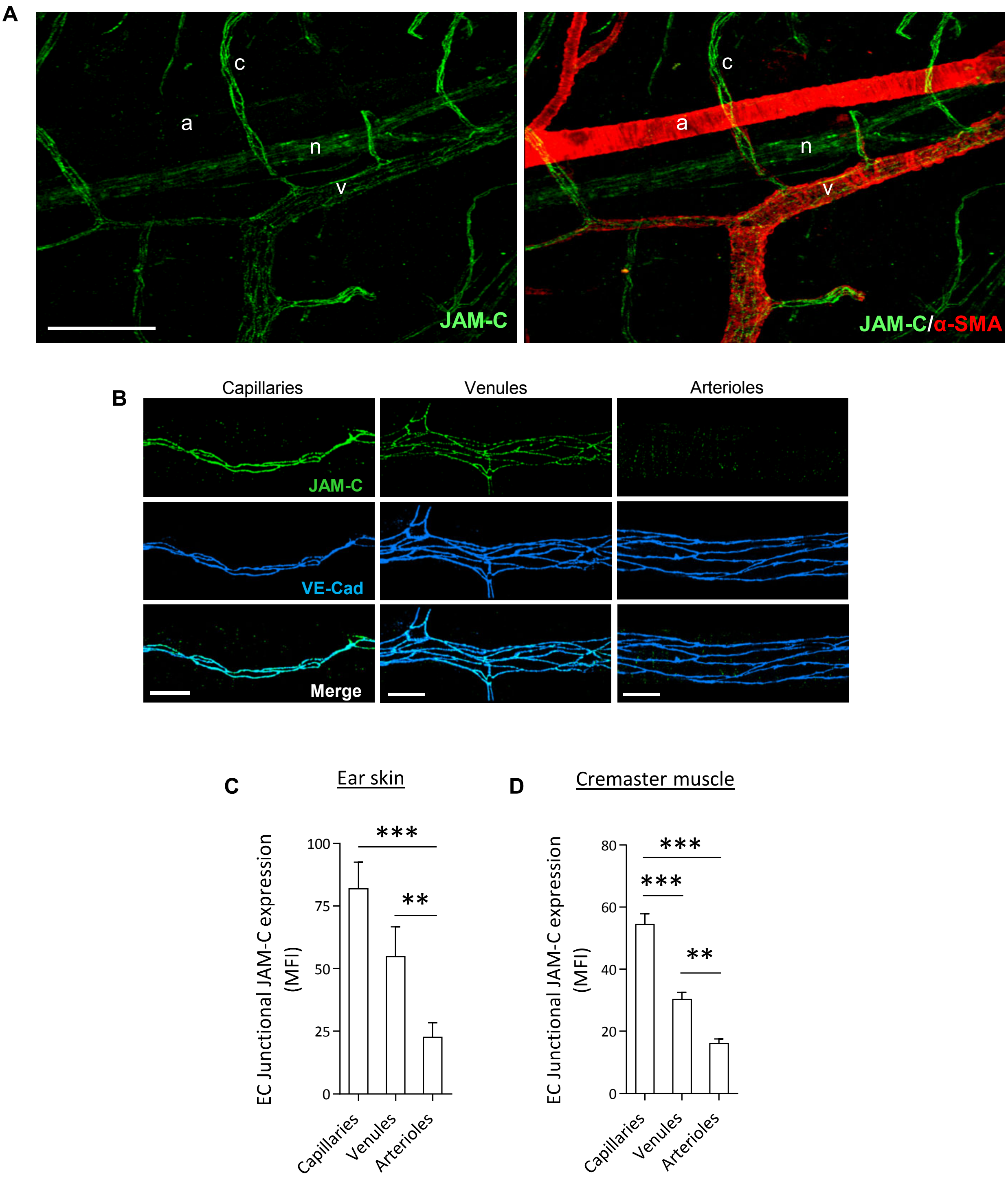
A
c
a
n
v
JAM-C
JAM-C/α-SMA
B
Capillaries
Venules
Arterioles
JAM-C
VE-Cad
Merge
C
Ear skin
D
Cremaster muscle
EC Junctional JAM-C expression (MFI)
Capillaries
Venules
Arterioles
***
**
0
25
50
75
100
20
40
60
80

Figure S2

Figure S2. Related to Figure 2. JAM-C is expressed at different levels in different types of microvessels. (A) Mouse ears were immunostained for JAM-C and α-SMA. The images show JAM-C expression in all microvessels but with differing levels: JAM-C expression was greatest in capillaries (c, α-SMA negative), followed by venules (v) and was low in arterioles (a). JAM-C was also noted in nerves (n). (B) High magnification images of mouse ear dermal blood vessels illustrating localization of JAM-C to EC junctions (as shown by co-localisation with VE-cadherin) and again indicating different expression levels of JAM-C in capillaries, venules and arterioles. (C-D) Quantification of JAM-C protein levels at junctions of ECs in different blood vessel types in ear skin (C) and cremasters (D), as analyzed by confocal microscopy (n=3-7) from 5 independent experiments. Data indicate mean fluorescent intensity (MFI) ± SEM. ** $P<0.01$ and *** $P<0.001$ as indicated by lines. Scale bars, 100μm (A), 20μm (B).

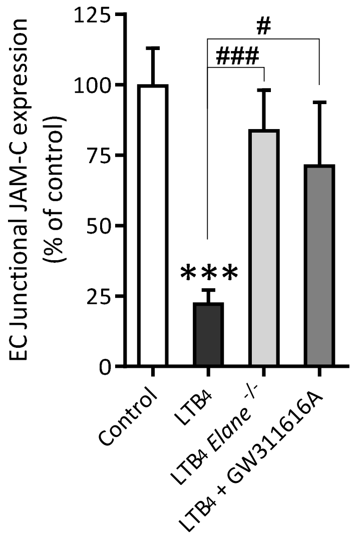

Figure S3. Related to Figure 3. Neutrophil elastase mediates LTB$_4$-induced cleavage of EC JAM-C. LTB$_4$-stimulated cremaster muscles of WT, *Elane*$^{-/-}$ or WT mice treated with the NE inhibitor GW311616A were analysed for junctional expression of EC JAM-C and compared to control unstimulated tissues (n=3-4) involving 4 independent experiments. Data indicate mean ± SEM. ***P<0.001 as compared to controls and $^{\#}$P<0.05 and $^{\#\#\#}$P<0.001 as indicated by lines.

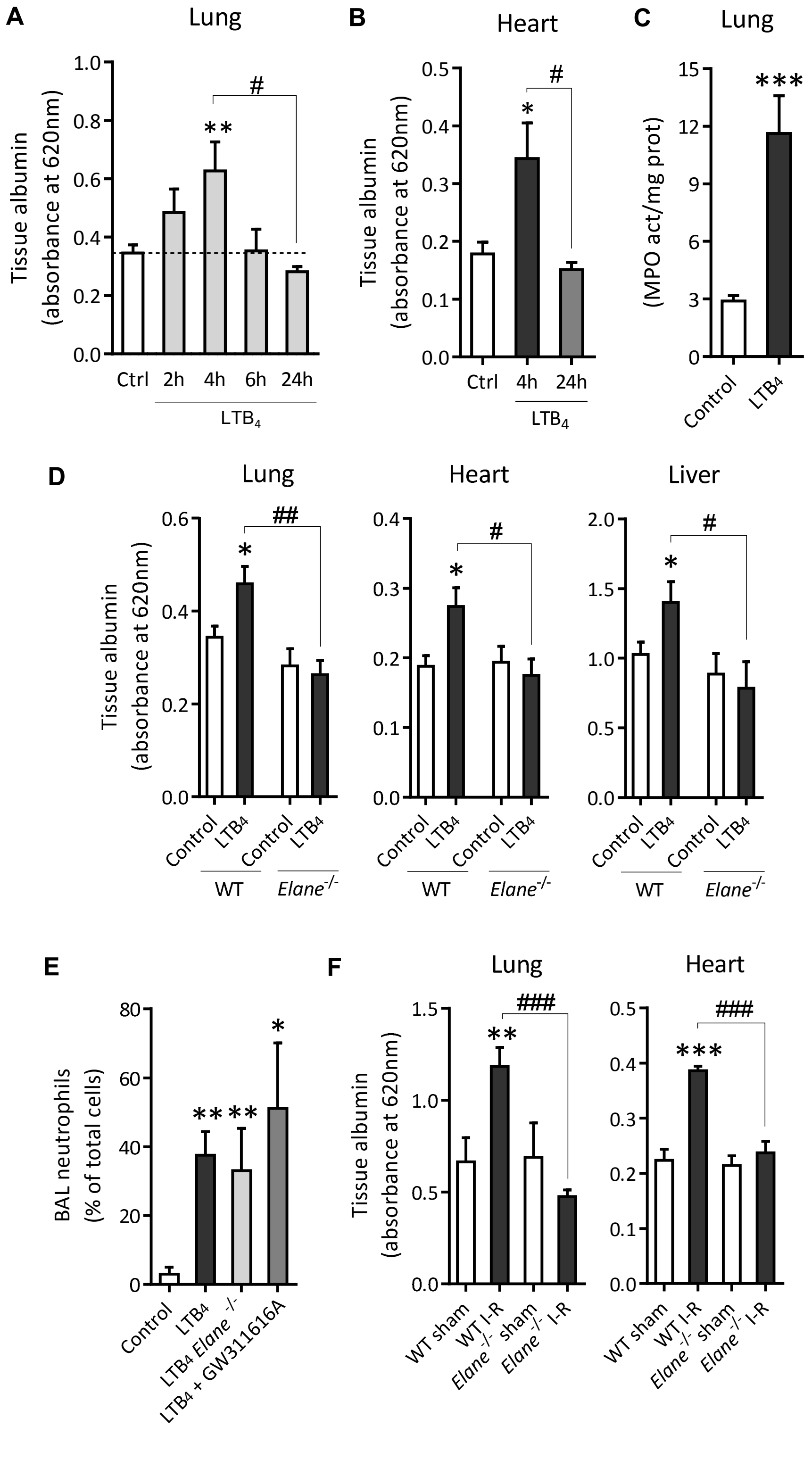
A
Lung
Tissue albumin (absorbance at 620nm)
Ctrl 2h 4h 6h 24h
LTB4
B
Heart
Tissue albumin (absorbance at 620nm)
Ctrl 4h 24h
LTB4
C
Lung
(MPO act/mg prot)
Control LTB4
D
Lung
Heart
Liver
Tissue albumin (absorbance at 620nm)
Control LTB4 Control LTB4
WT Elane-/-
E
BAL neutrophils (% of total cells)
Control
LTB4
LTB4 Elane -/-
LTB4 + GW311616A
F
Lung
Heart
Tissue albumin (absorbance at 620nm)
WT sham
WT I-R
Elane-/- sham
Elane-/- I-R

Figure S4

Figure S4. Related to Figure 6. The LTB_4-NE axis promotes distant organ damage. (A-B) Time course of remote organ damage following LTB_4 stimulation of cremaster muscle. Quantification of lung (A) and heart (B) albumin content (plasma extravasation), as an indicator of tissue damage, following local (cremaster, intrascrotal) injection of LTB_4 at the indicated time points as compared to control (Ctrl) unstimulated tissues (n=3-20 from 15 independent experiments). (C) Intradermal administration of LTB_4 into the mouse ear skin promotes lung inflammation. LTB_4 was injected into mouse ears intradermally and 4h later, the lungs were excised and analysed for neutrophil infiltration as quantified by measurement of tissue MPO enzymatic activity. Unstimulated ears acted as controls (n=4-6 from 2 independent experiments). (D) Intradermal injection of LTB_4 in the mouse ear promotes multi-organ distant damage in an NE-dependent manner. Quantification of remote tissue albumin content following local (ear, intradermal) injection of LTB_4 (4h) as compared to control unstimulated tissues in WT and *Elane*$^{-/-}$ mice (n=4-24 from 11 independent experiments). (E) LTB_4-induced neutrophil recruitment to lungs is NE-independent. WT, *Elane*$^{-/-}$ mice or WT mice pretreated (orally, 24h) with the NE inhibitor GW311616A were stimulated with intranasal LTB_4 and 24h later neutrophil infiltration into the airways was quantified in bronchoalveolar lavage (BAL). Control mice received LTB_4 vehicle (n=3-6 from 3 independent experiments). (F) Cremaster I-R injury induces multi-organ distant damage. Quantification of remote tissue albumin content in WT and *Elane*$^{-/-}$ mice following I-R injury of the cremaster muscle as compared to sham operated mice (n=3-7 from 5 independent experiments). Data indicate mean ± SEM. *P<0.05, **P<0.01 and ***P<0.001 as compared to controls and $^{\#}$P<0.05, $^{\#\#}$P<0.01 and $^{\#\#\#}$P<0.001 as indicated by lines.

SUPPLEMENTAL MOVIE LEGENDS

Movie S1. Related to Figure 1. Neutrophil reverse TEM as induced by I-R injury. The movie captures an inflammatory response in a cremasteric venule of a *Lyz2-EGFP-ki* mouse (exhibiting GFP myeloid cells), immunostained *in vivo* for EC junctions with Alexa Fluor-555-labeled anti-PECAM-1 mAb 390 (red) and stimulated with I-R. The clip shows high optical zoom of a neutrophil migrating through a multi-cellular junction viewed from the luminal side. The neutrophil (green) is initially on the abluminal (sub-EC) side of the endothelial junction and subsequently migrates through the junction in an abluminal to luminal or 'reverse' direction. Breaching the EC barrier results in the transient formation of an exit pore, as indicated in the movie. On the luminal side the leukocyte disengages from the junction and crawls across the luminal surface. Still images of this sequence are shown in Fig. 1D. Of note, the movie has been created using a software (IMARIS™, Bitplane) that recreates the structures being imaged from individual voxels in 3D via a blend projection algorithm whilst maintaining a transparency function. As a result, when observing neutrophil migration from the luminal side, neutrophils in the vascular lumen are seen as being fully green (closest to the viewing direction) whilst cells in the sub-EC space and/or within EC junctions are visualized as green cells with an overlay of red fluorescence (further away from the viewing direction). Similarly when viewing events from the abluminal side, the sub-EC neutrophil is seen as being fully green due to it being close to the viewing direction.

Movie S2. Related to Figure 1. Neutrophil reverse TEM as induced by topical LTB_4. The movie captures an inflammatory response in a cremasteric venule of a *Lyz2-EGFP-ki* mouse (exhibiting GFP myeloid cells, green), immunostained *in vivo* for EC junctions with Alexa Fluor-555-labeled anti-PECAM-1 mAb 390 (red), as induced by locally administered LTB_4. The clip shows high optical zoom of a neutrophil migrating through a multi-cellular junction viewed from the luminal side. The neutrophil (green) is initially on the luminal side of the endothelial cell, transmigrates into the sub-endothelial cell space and subsequently migrates through the junction back into the lumen in a 'reverse' direction. On the luminal side the leukocyte disengages from the junction and crawls across the luminal surface. The movie has been created using a software (IMARIS™, Bitplane) that recreates the structures being imaged from individual voxels in 3D via a blend projection algorithm whilst maintaining a transparency function. As a result, since the neutrophil rTEM event is being observed from the luminal side, neutrophils in the vascular lumen are seen as being fully green (closest to the

viewing direction) whilst cells in the sub-EC space and/or within EC junctions are visualized as green cells with an overlay of red fluorescence (further away from the viewing direction).

SUPPLEMENTAL EXPERIMENTAL PROCEDURES

Reagents/antibodies. Recombinant murine C5a, CXCL2 (MIP-2) and IL1β were purchased from R&D Systems (Abingdon, Oxford, UK). CXCL1 (KC) was from AbD Serotec (Oxford, UK). LTB_4 and PVDF membranes were purchased from Calbiochem (Merck Millipore, Nottingham UK). Tyrode's salt, FCS, PFA, EDTA, Triton X-100, HEPES solution, BSA, GW311616A, Ripa buffer, collagenase, Human MPO, purified Human IgG, glutaraldehyde, DNAse, anti-mouse α-SMA antibody (clone 1A4), lipopolysaccharide (LPS) and Evans blue were from Sigma-Aldrich (Poole, Dorset, UK). LY293111 was obtained from Cambridge Bioscience (Cambridge, UK). Recombinant murine JAM-C was generated as described before (Aurrand-Lions et al., 2001). JAM-B-Fc, Fc control protein, Recombinant human ICAM-1, Mouse Cytokine Array Panel A Array Kit and KC, IL1β and LTB_4 ELISA kits were from R&D systems (Abingdon, Oxford, UK). NE680FAST was obtained from Perkin Elmer (Buckinghamshire, UK). NGS was from PAA Laboratories (Somerset, UK). Purified human NE was purchased from Enzo Life Sciences (Exeter, UK). Halt Protease Phosphatase Inhibitor Cocktail, Supersignal West Pico Chemoluminescent Substrate and 16-well glass chamber slides (NUNC) were from Thermo Scientific (Cramlington, UK). Formamide and Sure Blue kit reagent were from VWR (Leicestershire, UK). Enhanced K-Blue TMB Substrate was from Neogen Corporation (Lexington, KY, USA). Anti-Ly-6G MicroBead Kit was from Miltenyi Biotec (Surrey, UK). RPMI 1640 medium was from Gibco (UK). Alexa-Fluor monoclonal antibody labelling kits, 2-mercaptoethanol, Dynabeads sheep anti-rat IgG and Alexa fluorescently-labelled secondary antibodies were from Invitrogen (Paisley UK). Anti-mouse antibodies against PECAM-1 (clone 390), VE-cadherin (clone BV14), CD11b (Mac-1, clone M1/70) and CD115 (clone AFS98), and the isotype controls IgG2b and IgG2a were purchased from eBiosciences (Hatfield, UK). Anti-JAM-A (clone H2O2-106-7-4) was a gift from Dr Michel Aurrand-Lions (INSERM, Centre de Recherche en Cancerologie de Marseille, France) and was generated as previously detailed (Malergue et al., 1998). Ultra-LEAF™ Purified anti-mouse Ly-6G (clone 1A8) was from BD Biosciences (Cowley, Oxford, UK). Anti-MRP14 (Hobbs et al., 2003) (clone 2B10) was a gift from Dr N. Hogg (Cancer Research UK, London,

UK). Rabbit polyclonal antibodies against CD11b (Mac-1) and NE were from Abcam (Cambridge, UK). Antibodies against Ly6G (clone 1A8) and CD45 (clone 30-F11) were obtained from Biolegend (London, UK). Rabbit Polyclonal anti-JAM-C was generated as previously described (Lamagna et al., 2005).

Animals. *Lyz2-EGFP-ki* mice (Faust et al., 2000) were used with the permission of Dr Thomas Graf (Center for Genomic Regulation and ICREA, Barcelona, Spain.) and were kindly provided by Dr Markus Sperandio (Ludwig-Maximilians University, Munich, Germany). In these animals the gene for *EGFP* has been knocked into the lysozyme M (*lyz2*) locus, yielding mice that exhibit fluorescent myelomonocytic cells, with mature neutrophils comprising the highest percentage of $EGFP^{hi}$ cells. Mice deficient in neutrophil elastase ($Elane^{-/-}$) (Belaaouaj et al., 1998) were a gift from Professor S Shapiro (Harvard Medical School, Boston, MA, USA). $Elane^{-/-}$ mice were crossed with *Lyz2-EGFP-ki* mice to generate a new colony ($Elane^{-/-}$*;Lyz2-EGFP-ki*) exhibiting NE deletion and GFP-tagged neutrophils. Endothelial cell specific JAM-C deficient mice (*Tekcre;JAM-3*$^{flox/flox}$) were generated in house as described before (Woodfin et al., 2011) by cre-mediated recombination of *JAM-3* flanked by *loxP* sites (Langer et al., 2011) under the control of the *Tek*-promoter. Wild type C57BL/6 mice were obtained from Harlan-Olac (Bicester, UK). All animal experiments were conducted in accordance with the United Kingdom Home Office legislations.

Patients. The study was approved by the East London and City Research Ethics Committee. All adult trauma patients (>15 years) who met the local criteria for trauma team activation were eligible for enrolment into the Activation of Coagulation and Inflammation in Trauma (ACIT) 2 study. ACIT2 is a study prospectively evaluating aspects of coagulation and inflammation in trauma patients. Exclusion criteria were; arrival at hospital more than 2-hours after injury, transfer from another hospital, known severe liver disease, known bleeding diathesis, administration of >2000ml of fluid prior to enrolment or a burn injury covering more than 5% of the total body surface area. Acute respiratory distress syndrome (ARDS) was defined using the Berlin consensus definitions (Force et al., 2012). Organ failure at 48h was described using the SOFA Score (Vincent et al., 1998).

Induction of inflammatory reactions and pre-treatments. Mice were anesthetized by intramuscular (i.m.) injection of 1ml/kg of anesthetic mix (40mg ketamine and 2mg xylazine in saline) before the inflammatory stimuli, namely LTB_4 (300ng), LPS (300ng), CXCL1 (KC)

(500ng), CXCL2 (MIP-2) (500ng), C5a (1μg) or vehicle control, were injected in the ears (30μl, intradermally, 4h) or the cremaster muscles (400μl, intrascrotally, 4h). In some experiments, purified human NE (1mg/kg) with or without KC (500ng) was injected locally for 4h into cremaster muscles and the NE inhibitor GW311616A (2mg/kg) (Macdonald et al., 2001) was orally administrated 24h before induction of inflammation. Cremaster I-R injury was induced in anesthetized mice as previously detailed (Scheiermann et al., 2009). Briefly, the blood flow to the muscle was stopped by placing a clamp at the base of the exteriorized tissue for 30 min to induce ischemia, after which the clamp was removed to allow reperfusion over a 2h period. Control sham operated mice underwent surgical procedures but not tissue I-R. In some animals the LTB_4 receptor antagonist LY293111 (10mg/Kg) was administered i.v. 15 minutes prior to induction of I-R.

Whole-mount tissue immunofluorescence staining. Ears or cremaster muscles were dissected and fixed for 10min in ice-cold PFA 4%. Samples were blocked/permeabilized for 2h at room temperature in PBS containing 12.5% FCS, 12.5% NGS and 0.5% Triton X-100, followed by overnight incubation at 4°C with primary antibodies in PBS containing 5% NGS and 5% FCS. For double or triple staining, some antibodies were directly conjugated with Alexa dyes using commercial Alexa-Fluor monoclonal antibody labelling kits. Otherwise, tissues were incubated with appropriate Alexa fluorescently-labelled secondary antibodies for 3h at 4°C in PBS containing 5% NGS and 5% FCS. After washings, tissues were mounted on slides and analyzed by confocal microscopy.

Confocal microscopy. Immunofluorescently stained whole-mount tissues were imaged using a Zeiss LSM 5 PASCAL confocal laser-scanning microscope (Carl Zeiss) equipped with Argon (excitation wavelength: 488nm) and HeNe (excitation wavelengths: 543 and 633nm) lasers, or a Leica SP5 (Leica) equipped with argon and helium-neon lasers. Multiple Z-stack images at a resolution of 1024 x 1024 were acquired with an oil immersion Plan-Apochromat 63x (1.4 NA) objective or a 20× water-dipping objective (1.0 NA). The protein expression of junctional EC adhesion molecules was quantified in 3D reconstructed images using IMARIS software (Bitplane) as described before (Colom et al., 2012; Woodfin et al., 2011). Briefly, an isosurface was created using the VE-Cadherin or PECAM-1 labelled channels and the intensity of immunoreactive proteins of interest within these channels was quantified. Total expression of JAM-C was analyzed using Image J software. The samples were also analyzed for

quantification of number of transmigrated neutrophils per field of view using the 3D images and IMARIS software.

Quantification of plasma soluble JAM-C content. Mouse blood was obtained by cardiac puncture and collected in heparin. Samples were centrifuged at 6000xg for 3 min and plasma was collected, frozen in liquid N_2 and kept at -80C°. Human plasma was prepared post double centrifugation of blood, collected in buffered sodium citrate, at 1760xg for 10 min and supernatants were then frozen and stored at -80C°. sJAM-C content in plasma was measured by ELISA as follows. ELISA plates were coated with mouse or human JAM-B-Fc or Fc control protein in bicarbonate buffer (100mM pH 9.6). Wells were blocked with a solution of PBS containing 0.05% Tween-20 (PBS-T), 3% BSA, 0.2% gelatin (for human only) and 10µg/ml of purified IgG. After washing in PBS-T, plasma samples diluted 1:1 in PBS-T were added to the wells and incubated overnight at 4°C. Wells were then washed twice in PBS-T, once in PB and then incubated with 10µg/ml monoclonal anti-mouse JAM-C (H36) or 5µg/ml affinity purified rabbit polyclonal anti-human JAM-C (714) (Lamagna et al., 2005; Ody et al., 2007). Finally, samples were incubated with a goat anti-rat or anti-rabbit antibody conjugated to HRP and the peroxidase activity was measured with Sure Blue kit reagent using a LEDETECT96 plate reader. Calculations were performed on the linear part of the calibration curve. Soluble mouse JAM-C of non-stimulated animals was at the limit of the detection level.

Cytokine/chemokine expression profile. Mouse cremasters were homogenized in 500µl PBS containing 1% Triton and 1% Halt Protease and Phosphatase Inhibitor Cocktail using the Precellys24 beat-beading system (Bertin Technologies, France). Samples were quickly frozen in liquid N_2, thawed, centrifuged 5 min at 10000xg and the supernatant collected for subsequent analysis. The cytokine/chemokine expression profile of the samples (pooled samples from 3 mice/group) was analyzed using a Mouse Cytokine Array Panel A Array Kit as per manufacturer instructions. Densitometry from the blots was analyzed with ImageJ software. The expression of selected inflammatory mediators (KC, IL1β and LTB_4) was analyzed by ELISA using commercial kits.

***In vitro* digestion of JAM-C.** Purified recombinant murine JAM-C (Aurrand-Lions et al., 2001) (5µg) was incubated for 1h at 37°C in digestion buffer consisting of 0.2M Tris, 0.15M NaCl and 0.02M $CaCl_2$ at pH 7.4, in the absence or presence of 0.05, 0.2 or 1µg of purified

NE, within a total final reaction volume of 15µl. Samples were then resolved in a SDS-PAGE gel and visualized by Western blot.

Co-immunoprecipitation. Bone marrow (BM) derived neutrophils were purified using an anti-Ly-6G MicroBead Kit as per the manufacturer's instructions. Neutrophils ($3x10^5$) were left unstimulated or stimulated with LTB_4 (100nM) for 30min at room temperature in RPMI medium containing HEPES (25mM), FCS (10%), GW311616A (5µM), Halt Protease-Phosphatase Inhibitor Cocktail (1%), purified NE (5µg) and JAM-C (5µg) (Aurrand-Lions et al., 2001). Samples were then lysed in RIPA buffer and pre-cleared with Dynabeads (20µl) sheep anti-rat IgG at 4°C for 1h. Dynabeads were removed by centrifugation (1min, 2000xg) and the supernatant was incubated overnight at 4°C with an anti-Mac-1 mAb (5µg). Samples were then incubated with Dynabeads for 3h at 4°C. Following centrifugation, Dynabeads were resuspended in PBS and boiled for 10min in loading buffer containing 2% 2-mercaptoethanol. JAM-C and NE protein content was analyzed both in lysates and anti-Mac-1 immunoprecipitated samples by Western blot.

Immuno blot. Samples were boiled for 10min in loading buffer containing 2% 2-mercaptoethanol and resolved on SDS-PAGE gels. Proteins were electrotransferred onto PVDF membranes followed by 1h blocking in TBS-Tween containing 5% non-fat milk and incubation overnight at 4°C with primary antibodies in 5% BSA TBS-Tween buffer. After incubation with HRP-conjugated secondary antibodies, membranes were developed using the Supersignal West Pico Chemoluminescent Substrate.

***In vitro* neutrophil adhesion assay.** 16-well glass chamber slides were coated with recombinant human ICAM-1 (2.5µg/ml) in coating buffer (150 mM NaCl, 20 mM Tris-HCl, 2 mM MgCl2, pH 9.0) overnight at 4°C. Slides were then washed and blocked with 10% BSA in PBS for 1h at room temperature. BM neutrophils were isolated from WT mice using an anti-Ly-6G MicroBead Kit. Neutrophils ($5x10^4$/well) in RPMI 1640 media, 10% FCS and 25 mM HEPES were either left untreated or stimulated with LTB_4 (1-100nM) or KC (1-100nM) for 30 min at room temperature. In some experiments neutrophils were pre-incubated with an anti-Mac-1 mAb or an isotype control IgG (both at 40 µg/ml) for 20 min at room temperature. Slides were then washed twice in PBS and fixed in 3% PFA + 0.5% glutaraldehyde in PBS for 2 hours on ice. The number of adherent cells per field of view was quantified from images acquired by phase contrast microscopy.

Imaging of JAM-C cleavage *in vitro*. 16-well glass chamber slides were coated with recombinant ICAM-1 and JAM-C (2.5µg/ml) as above. BM neutrophils (5x10^4/well) were left untreated or treated with LTB_4 (100nM), LTB_4+ GW311616A (5µM) or KC (100nM) for 30min. Slides were then fixed as above and immunostained with antibodies against JAM-C and MRP-14. Slides were analyzed by confocal microscopy and the expression of JAM-C at sites of neutrophil adhesion was quantified with IMARIS software by creating an isosurface on the MRP-14 channel and measuring the intensity of JAM-C within this surface. In some experiments, *Elane*$^{-/-}$ BM neutrophils were pre-incubated with an anti-Mac-1 (40µg/ml) or isotype control mAb prior to being stimulated with LTB_4 (30 min) in the presence or absence of purified NE (0.1mg/ml).

Mouse neutrophil depletion protocol. Specific depletion of circulating neutrophils was achieved by a single i.p. injection of 150 µg of Ultra-LEAF™ Purified anti-mouse Ly-6G mAb for 24h. The protocol resulted in >99% depletion of circulating neutrophils as determined by flow cytometry, while the number of monocytes was not affected. Control non-depleted groups were injected with an isotype-matched mAb.

***In vivo* NE enzymatic activity assay.** The NE-fluorescent activatable substrate NE680FAST (Kossodo et al., 2011) was injected i.v. (4.8nmols) into anesthetized mice and left to circulate for 15 min before induction of ear inflammation as detailed above. Tissues were then collected, fixed in 4% PFA as described above and immunofluorescently stained for VE-Cadherin before being analyzed by confocal microscopy. NE activity was quantified by the fluorescence of the peptide NE680FAST, as measured using ImajeJ software.

***In vitro* NE enzymatic activity assay.** The NE-fluorescent activatable substrate NE680FAST was used to assay NE activity released from BM neutrophils *in vitro*. Briefly cells (5x10^4/well) on ICAM-1 and JAM-C coated slides were left untreated or stimulated for 30min with KC (100nM) or LTB_4 (100nM) in the presence of NE680FAST (1µM/well). Slides were then washed, fixed, immunostained for neutrophils using an anti-MRP-14 mAb, and the intensity of NE680FAST on the isosurface of MRP-14 channel was analyzed by confocal microscopy and measured with IMARIS software as detailed above.

Confocal intravital microscopy (IVM) of mouse cremaster muscles. Confocal intravital microscopy analysis of the mouse cremaster muscle was conducted as previously detailed (Woodfin et al., 2011). Briefly, *Lyz2-EGFP-ki* mice (exhibiting predominantly $EGFP^{hi}$ neutrophils) were injected (i.s.) with an Alexa 555-conjugated mAb against PECAM-1 (4μg) for 2h to stain EC junctions of the cremaster microvasculature. After cremaster exteriorization, postcapillary venules (20-40μm diameter) of stimulated tissues were selected for *in vivo* analysis of leukocyte-vessel wall interactions using a Leica SP5 confocal microscope incorporating a 20× water-dipping objective (NA 1.0). Acquisition of 3D confocal images over time yielded high-resolution four-dimensional videos of dynamic events that were analysed with IMARIS 4D modelling software (Bitplane; for more details see below). Neutrophils exhibiting reverse TEM (rTEM) were defined as cells that moved in an abluminal-to-luminal direction within endothelial cell junctions (stained with an anti-PECAM-1 mAb). This included cells that fully or partially breached the endothelium from the vascular lumen before exhibiting reverse motility through EC junctions and re-entering the blood flow. Within this overall definition, neutrophils were observed to fully breach EC junctions and completely enter the sub-endothelial cell space (where on occasion sub-EC motility was observed), before reverse migrating back through the endothelium into the vascular lumen. In some instances, neutrophils exhibited partial migration into EC junctions (~70-80% of the cell body), before reverse migrating towards the vascular lumen and re-entering the blood circulation. In all cases of neutrophil rTEM, the cells ultimately showed reverse motility within EC junctions (abluminal-to-luminal), ending up in the vascular lumen after disengagement from EC junctions. In contrast, normal neutrophil TEM was classified as a response in which the cells migrated through EC junctions only in a luminal-to-abluminal direction and with no pause (Woodfin et al., 2011).

All the movies and images are representative 4D and 3D images, respectively, acquired using a complex algorithmic software (IMARIS™, Bitplane). This software recreates the structures being imaged from individual voxels in 3D by using a blend projection algorithm that enables the mixing of voxel values along the viewing direction whilst maintaining a transparency function. In the movies, rTEM responses are largely being viewed from the luminal side of the vessel, which results in a visual overlap of voxels from the Alexa Fluor 555 (PECAM-1; closer to the viewing point) with that of GFP-neutrophils (further away from the viewing point). Hence when the neutrophil is in the sub-EC space or at EC junctions, the leukocyte is seen to be green with an overlay of red fluorescence. In contrast, when the neutrophil is in the vascular

lumen, the voxels nearest to the viewing direction stem from GFP, resulting in the neutrophils being totally green.

Analysis of lung neutrophil infiltration. Following stimulation of cremaster muscles, the left carotid artery of anesthetized mice was cannulated, a bolus injection of heparin (50U) administered and the mice fully exsanguinated. After ligation of the carotid artery, the cannula was removed and mice were killed by cervical dislocation. The chest cavity was immediately opened and both the thoracic vena cava and aorta just above the diaphragm were clamped. The pulmonary vasculature was then perfused with 10 ml of warm PBS (containing heparin and EDTA) by direct injection into the right ventricle, and collection via a cannula inserted into the left ventricle. Lungs were then excised and digested for 30min at 37°C in 5 ml PBS containing collagenase and DNAse (500 U each). The digested lungs were passed through 40 μm cell strainers and the collected cell suspension centrifuged at 400xg for 10min at 4°C. Finally, cells were stained and the number of neutrophils analyzed by flow cytometry as detailed below.

Flow cytometry. The efficiency and specificity of the leukocyte depletion protocol as well as lung neutrophil counts were assessed by flow cytometry. Samples were collected and incubated with anti-mouse CD16-CD32 to block Fc receptor-mediated antibody binding (5μg/ml), before staining with fluorescently conjugated antibodies against the pan leukocyte marker CD45, the monocyte marker CD115 and the neutrophil marker Ly6G. Red blood cells were then lysed with ACK lysis buffer (150mM NH_3Cl, 1mM $KHCO_3$ and 1mM EDTA) and immunoreactive molecules of interest were measured on a LSR Fortessa flow cytometer (BD) and analyzed using Flowjo software (TreeStar).

MPO enzymatic activity assay. Mouse ears were stimulated i.d. for 4h with LTB_4 or vehicle control. Animals were killed and lungs were excised and homogenized in 1ml of homogenizing buffer (600mM NaCl, 0.5% HTAB, 600mM KH_2PO_4 and 66mM Na_2HPO_4) using the Precellys24 beat-beading system (Bertin Technologies, France). Samples were then subjected to two cycles of liquid N_2 freezing-thawing and homogenized again followed by centrifugation at 13000xg (10min at 4°C). MPO activity of lung supernatants was measured through the use of Enhanced K-Blue TMB Substrate (Oxford Byosystems) with the increase in absorbance at 650nm being measured every 30s for 15min at 37°C using a spectrophotometer (Spectra MR, Dynex Technologies). The enzyme activity was calculated using a standard curve generated with human MPO and expressed as Units/mg of protein.

Analysis of neutrophils in the bronchoalveolar lavage (BAL). Mice were intranasally challenged with LTB_4 (2µg) or vehicle control. After 24h, animals were anesthetized, the chest cavity was opened and the trachea exposed. A catheter was then inserted into the trachea and used to flush the lungs with 2ml PBS containing 0.5mM EDTA. BAL fluid was collected and the % of neutrophils was analyzed by flow cytometry as described above.

Measurement of tissue plasma extravasation. Following local stimulation of cremaster muscles or ears (intradermal) with CXCL1 (KC) (500ng), LTB_4 (300ng) or I-R injury (cremaster) in mice with or without pre-treatment with GW311616A (2mg/kg, 24h, orally) (Macdonald et al., 2001), Evans blue solution (5µl of a 5% solution per gram of mouse weight,) was injected i.v. into the mice and allowed to circulate for 10 min before the animals were killed. Following vascular wash-out (with PBS containing 5mM EDTA), tissues were collected and the accumulated Evans blue (tissue albumin) was eluted in 1ml of formamide for 24 h at 55°C. Optical density (OD) readings at 620 nm were normalized to formamide alone and used as a measure of plasma extravasation.

SUPPLEMENTAL REFERENCES

Aurrand-Lions, M., Duncan, L., Ballestrem, C., and Imhof, B.A. (2001). JAM-2, a novel immunoglobulin superfamily molecule, expressed by endothelial and lymphatic cells. J Biol Chem *276*, 2733-2741.

Colom, B., Poitelon, Y., Huang, W., Woodfin, A., Averill, S., Del Carro, U., Zambroni, D., Brain, S.D., Perretti, M., Ahluwalia, A.*, et al.* (2012). Schwann cell-specific JAM-C-deficient mice reveal novel expression and functions for JAM-C in peripheral nerves. FASEB J *26*, 1064-1076.

Faust, N., Varas, F., Kelly, L.M., Heck, S., and Graf, T. (2000). Insertion of enhanced green fluorescent protein into the lysozyme gene creates mice with green fluorescent granulocytes and macrophages. Blood *96*, 719-726.

Force, A.D.T., Ranieri, V.M., Rubenfeld, G.D., Thompson, B.T., Ferguson, N.D., Caldwell, E., Fan, E., Camporota, L., and Slutsky, A.S. (2012). Acute respiratory distress syndrome: the Berlin Definition. JAMA *307*, 2526-2533.

Hobbs, J.A., May, R., Tanousis, K., McNeill, E., Mathies, M., Gebhardt, C., Henderson, R., Robinson, M.J., and Hogg, N. (2003). Myeloid cell function in MRP-14 (S100A9) null mice. Mol Cell Biol *23*, 2564-2576.
Kossodo, S., Zhang, J., Groves, K., Cuneo, G.J., Handy, E., Morin, J., Delaney, J., Yared, W., Rajopadhye, M., and Peterson, J.D. (2011). Noninvasive in vivo quantification of neutrophil elastase activity in acute experimental mouse lung injury. Int J Mol Imaging *2011*, 581406.
Lamagna, C., Hodivala-Dilke, K.M., Imhof, B.A., and Aurrand-Lions, M. (2005). Antibody against junctional adhesion molecule-C inhibits angiogenesis and tumor growth. Cancer Res *65*, 5703-5710.
Langer, H.F., Orlova, V.V., Xie, C., Kaul, S., Schneider, D., Lonsdorf, A.S., Fahrleitner, M., Choi, E.Y., Dutoit, V., Pellegrini, M.*, et al.* (2011). A Novel Function of Junctional Adhesion Molecule-C in Mediating Melanoma Cell Metastasis. Cancer Res *71*, 4096-4105.
Macdonald, S.J., Dowle, M.D., Harrison, L.A., Shah, P., Johnson, M.R., Inglis, G.G., Clarke, G.D., Smith, R.A., Humphreys, D., Molloy, C.R.*, et al.* (2001). The discovery of a potent, intracellular, orally bioavailable, long duration inhibitor of human neutrophil elastase--GW311616A a development candidate. Bioorg Med Chem Lett *11*, 895-898.
Malergue, F., Galland, F., Martin, F., Mansuelle, P., Aurrand-Lions, M., and Naquet, P. (1998). A novel immunoglobulin superfamily junctional molecule expressed by antigen presenting cells, endothelial cells and platelets. Mol Immunol *35*, 1111-1119.
Ody, C., Jungblut-Ruault, S., Cossali, D., Barnet, M., Aurrand-Lions, M., Imhof, B.A., and Matthes, T. (2007). Junctional adhesion molecule C (JAM-C) distinguishes CD27+ germinal center B lymphocytes from non-germinal center cells and constitutes a new diagnostic tool for B-cell malignancies. Leukemia *21*, 1285-1293.
Vincent, J.L., de Mendonca, A., Cantraine, F., Moreno, R., Takala, J., Suter, P.M., Sprung, C.L., Colardyn, F., and Blecher, S. (1998). Use of the SOFA score to assess the incidence of organ dysfunction/failure in intensive care units: results of a multicenter, prospective study. Working group on "sepsis-related problems" of the European Society of Intensive Care Medicine. Crit Care Med *26*, 1793-1800.
