# Supplementary figures and images for: Leukotriene B4-Neutrophil Elastase Axis Drives Neutrophil Reverse Transendothelial Cell Migration In Vivo
Source: Immunity. 2015 Jun 16;42(6):1075–86. doi: 10.1016/j.immuni.2015.05.010 (PMC4504024; doi:10.1016/j.immuni.2015.05.010)

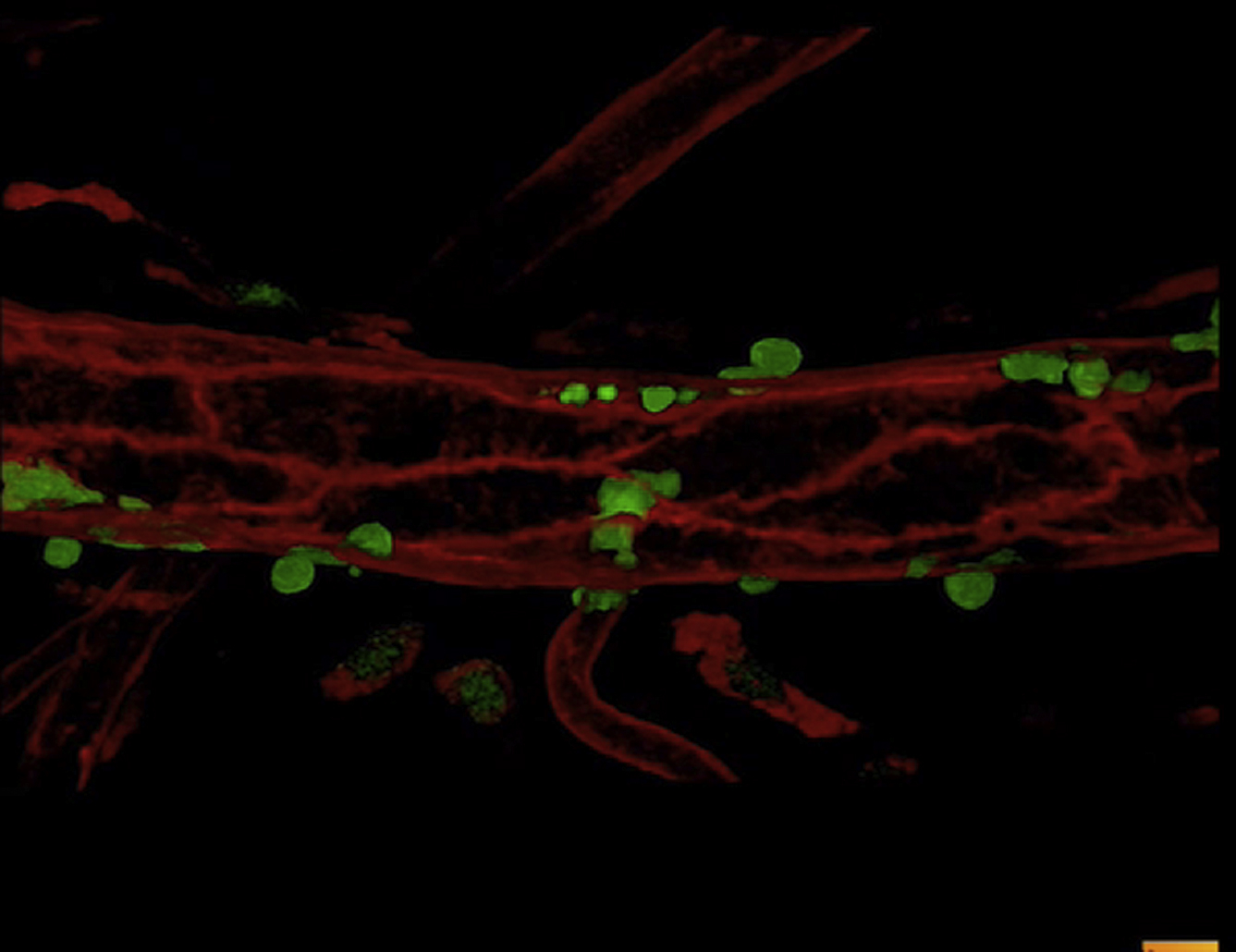

Supplement: Movie S1. Neutrophil Reverse TEM as Induced by I-R Injury, Related to Figure 1 [file mmc2.jpg]

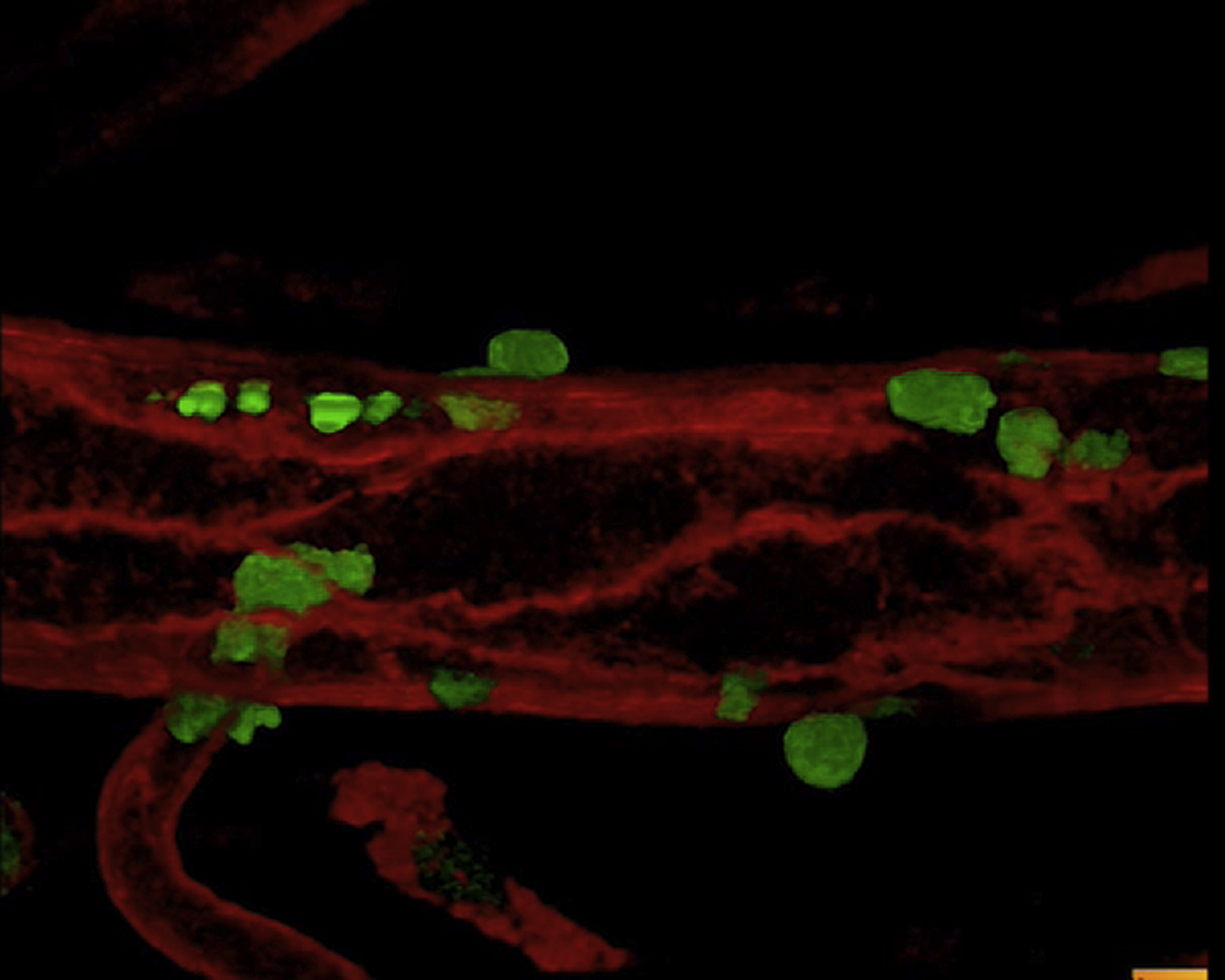

Supplement: Movie S2. Neutrophil Reverse TEM as Induced by Topical LTB4, Related to Figure 1 [file mmc3.jpg]
