# Supplementary material for: Leukotriene B4-Neutrophil Elastase Axis Drives Neutrophil Reverse Transendothelial Cell Migration In Vivo
Source: Immunity. 2015 Jun 16;42(6):1075–86. doi: 10.1016/j.immuni.2015.05.010 (PMC4504024; doi:10.1016/j.immuni.2015.05.010)
Supplement: Document S2. Article plus Supplemental Information [file mmc4.pdf]

Immunity

Article

Leukotriene B_4-Neutrophil Elastase Axis Drives Neutrophil Reverse Transendothelial Cell Migration In Vivo

Graphical Abstract

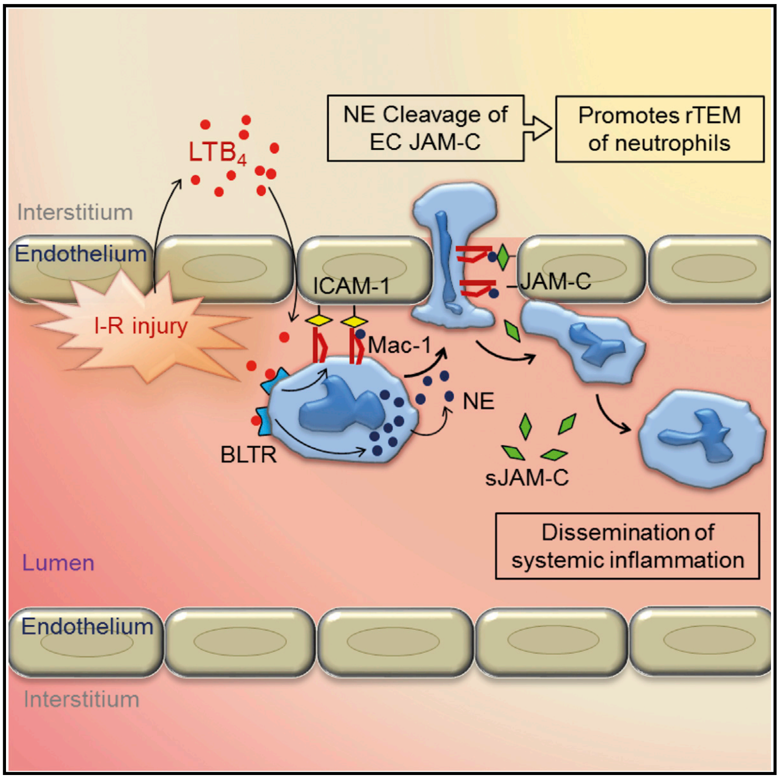

Highlights

- Endogenous LTB_4 mediates reduced expression of endothelial cell (EC) JAM-C in I-R
- LTB_4 can stimulate proteolytic cleavage of EC JAM-C by neutrophil elastase (NE)
- Activation of a local LTB_4-NE axis induces neutrophil reverse TEM in vivo
- Activation of a local LTB_4-NE axis can promote distant organ damage

Authors

Bartomeu Colom, Jennifer V. Bodkin, Martina Beyrau, ..., Karim Brohi, Beat A. Imhof, Sussan Nourshargh

Correspondence

s.nourshargh@qmul.ac.uk

In Brief

The mechanisms and implications of aberrant modes of neutrophil transendothelial cell migration remain largely unknown. Here, Nourshargh and colleagues demonstrate that local leukotriene B_4-induced proteolytic cleavage of endothelial JAM-C by neutrophil elastase promotes neutrophil reverse transendothelial cell migration back into the circulation and is decisive in dissemination of systemic inflammation.

Colom et al., 2015, Immunity *42*, 1075–1086
June 16, 2015 ©2015 The Authors
http://dx.doi.org/10.1016/j.immuni.2015.05.010

Immunity
Article

Leukotriene B_4-Neutrophil Elastase Axis Drives Neutrophil Reverse Transendothelial Cell Migration In Vivo

Bartomeu Colom,[1] Jennifer V. Bodkin,[1] Martina Beyrau,[1] Abigail Woodfin,[1] Christiane Ody,[2] Claire Rourke,[3] Triantafyllos Chavakis,[4] Karim Brohi,[3] Beat A. Imhof,[2] and Sussan Nourshargh[1,*]
[1]William Harvey Research Institute, Barts and The London School of Medicine and Dentistry, Queen Mary University of London, Charterhouse Square, London EC1M 6BQ, UK
[2]Centre Médical Universitaire, Rue Michel-Servet 1, Geneva CH-1211, Switzerland
[3]Centre for Trauma Sciences, Barts and The London School of Medicine and Dentistry, Queen Mary University of London, Mile End Road, London E1 4NS, UK
[4]Department of Clinical Pathobiochemistry, University of Dresden, Fetscherstrasse 74, Dresden 01307, Germany
*Correspondence: s.nourshargh@qmul.ac.uk
http://dx.doi.org/10.1016/j.immuni.2015.05.010
This is an open access article under the CC BY license (http://creativecommons.org/licenses/by/4.0/).

SUMMARY

Breaching endothelial cells (ECs) is a decisive step in the migration of leukocytes from the vascular lumen to the extravascular tissue, but fundamental aspects of this response remain largely unknown. We have previously shown that neutrophils can exhibit abluminal-to-luminal migration through EC junctions within mouse cremasteric venules and that this response is elicited following reduced expression and/or functionality of the EC junctional adhesion molecule-C (JAM-C). Here we demonstrate that the lipid chemoattractant leukotriene B_4 (LTB_4) was efficacious at causing loss of venular JAM-C and promoting neutrophil reverse transendothelial cell migration (rTEM) in vivo. Local proteolytic cleavage of EC JAM-C by neutrophil elastase (NE) drove this cascade of events as supported by presentation of NE to JAM-C via the neutrophil adhesion molecule Mac-1. The results identify local LTB_4-NE axis as a promoter of neutrophil rTEM and provide evidence that this pathway can propagate a local sterile inflammatory response to become systemic.

INTRODUCTION

Neutrophil infiltration into interstitial tissues is a critical component of the innate immune response and a hallmark of acute inflammatory reactions. Due to the destructive potential of neutrophils, this response is also intimately associated with the pathogenesis of numerous inflammatory conditions such as ischemia-reperfusion (I-R) injury, rheumatoid arthritis, and atherosclerosis (Nathan, 2006; Phillipson and Kubes, 2011; Mócsai, 2013). Neutrophil migration out of the vasculature is classically described by the leukocyte adhesion cascade that depicts a well characterized sequence of cellular and molecular events within the vascular lumen as orchestrated by numerous stimulatory and adhesive pathways (Ley et al., 2007). Less is known about the stages beyond the vascular lumen, though there is a growing understanding of the adhesive interactions that mediate neutrophil interactions with components of venular walls (Ley et al., 2007; Nourshargh et al., 2010; Proebstl et al., 2012; Nourshargh and Alon, 2014) and the molecular and cellular regulation of neutrophil motility in the interstitial tissue (Lämmermann et al., 2013; Weninger et al., 2014).

The migration of neutrophils through the endothelial cell (EC) barrier can occur via both paracellular and transcellular modes (Nourshargh et al., 2010; Kolaczkowska and Kubes, 2013) though the former is considered to be the most prevalent in the peripheral circulation (Schulte et al., 2011; Woodfin et al., 2011). This response is known to be mediated by numerous EC junctional molecules including platelet endothelial cell adhesion molecule-1 (PECAM-1), members of the junctional adhesion molecule (JAM) family, ICAM-2, VE-cadherin, and CD99 (Nourshargh et al., 2010; Voisin and Nourshargh, 2013; Vestweber et al., 2014). In addition to moving from the vascular lumen to the extravascular tissue, there is now unequivocal evidence for the ability of neutrophils to exhibit reverse motility through the endothelium. Specifically, through the application of a high resolution confocal intravital microscopy (IVM) platform to analysis of leukocyte transmigration in the mouse cremaster muscle, we have noted that neutrophils can exhibit migration through EC junctions in an abluminal-to-luminal direction (Woodfin et al., 2011). This neutrophil rTEM response is most prevalent in tissues subjected to the sterile injury caused by I-R, an inflammatory insult that is associated with reduced expression of JAM-C at EC junctions (Scheiermann et al., 2009; Woodfin et al., 2011). Furthermore, pharmacological blockade or genetic deletion of EC JAM-C enhances the frequency of neutrophil rTEM through cremasteric venules (Woodfin et al., 2011) and blockade of EC JAM-C has been shown to promote monocyte rTEM through cultured human umbilical vein ECs (HUVECs) (Bradfield et al., 2007). Together, these results have identified EC junctional JAM-C as a regulator of polarized movement of leukocytes from the vascular lumen toward the sub-EC space. While the phenomenon of neutrophil rTEM has been illustrated using in vitro models of neutrophil TEM (Buckley et al., 2006)

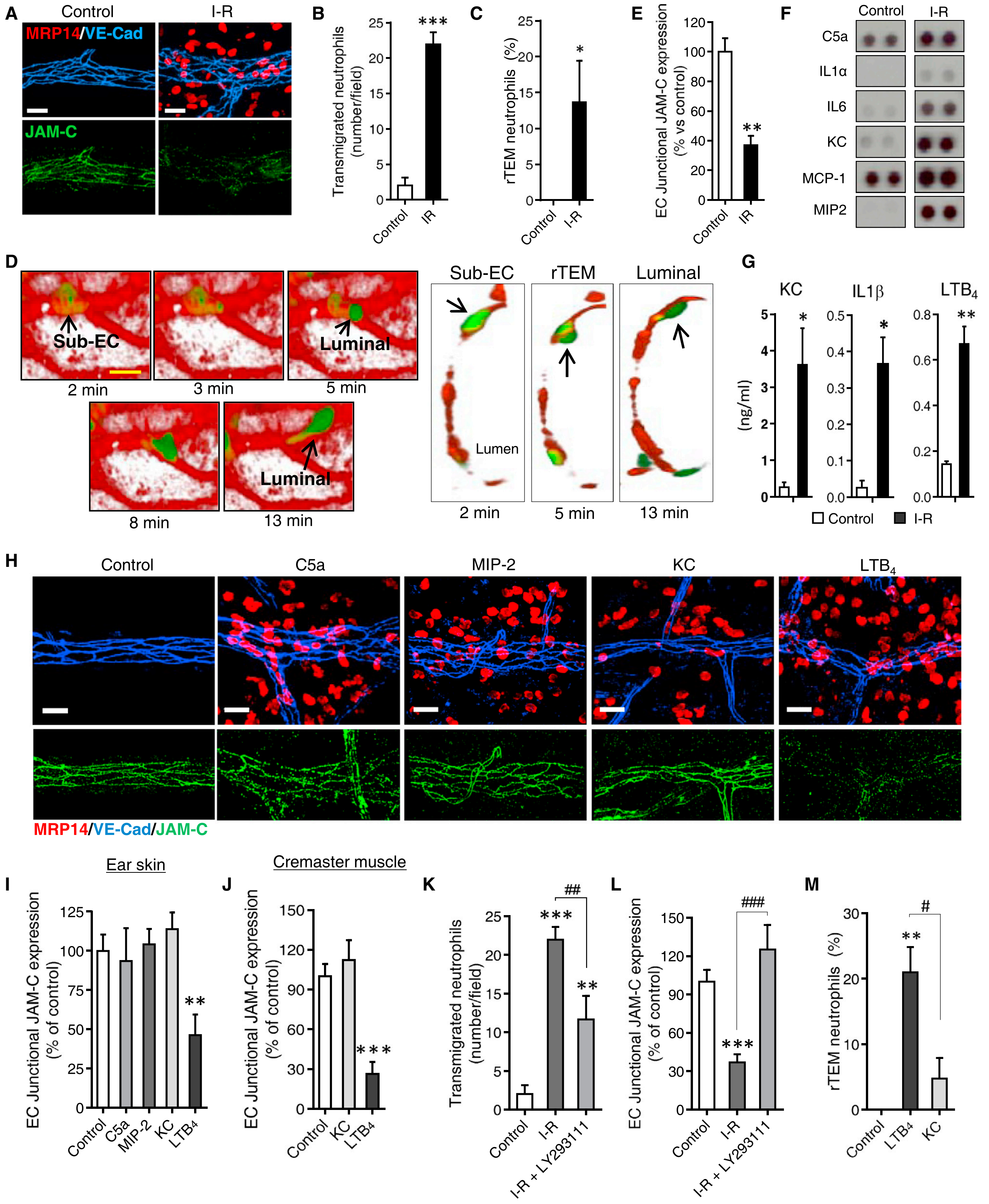

Figure 1. LTB_4 Mediates I-R Induced Loss of EC JAM-C

(A) Confocal images of mouse cremasteric venules in control or I-R stimulated tissues immunostained for MRP14 (neutrophils), VE-cadherin, and JAM-C. Images are representative of three independent experiments.

(legend continued on next page)

and subsequently in vivo within zebrafish embryos (Mathias et al., 2006), our findings within the mouse cremaster muscle provide direct evidence for neutrophil rTEM within a mammalian system, urging a need for better understanding of this unexpected response (Woodfin et al., 2011).

To acquire a greater insight into the frequency, regulation, and pathophysiological role of neutrophil rTEM here, we sought to identify the inflammatory trigger(s) that promote neutrophil rTEM in response to I-R. Specifically, because reduced expression and/or functionality of EC JAM-C was instrumental in promoting neutrophil rTEM in vivo, we investigated the mechanism through which EC JAM-C was lost at sites of sterile injury. The results identified endogenous leukotriene B_4 (LTB_4) as the mediator responsible for I-R-elicited loss of venular JAM-C and showed that exogenous LTB_4 was highly efficacious at lowering the expression of EC JAM-C in vivo. This effect was neutrophil-dependent, with neutrophil elastase (NE) governing the cleavage of EC JAM-C at sites of intense neutrophil infiltration. Furthermore, local LTB_4 and NE could both promote notable neutrophil rTEM. Although investigations into the pathophysiological relevance of neutrophil rTEM are at a developing stage, our previous findings suggested an association between neutrophil rTEM and distant organ inflammation (Woodfin et al., 2011). In line with this possibility, here we show that activation of LTB_4–NE axis can drive a local inflammatory response to become a systemic multi-organ reaction, providing further evidence for an association between occurrence of neutrophil rTEM and development of secondary organ inflammation.

RESULTS

Endogenously Generated LTB_4 Accounts for Reduced Expression of Local EC JAM-C in Response to I-R

The mechanism of EC JAM-C reduction was investigated in a murine model of cremaster muscle I-R (30 min ischemia, 2 hr reperfusion), an injury model that is amenable to rapid and high-resolution intravital microscopy (Woodfin et al., 2011). This sterile injury model is characterized by profound local neutrophil infiltration and is associated with increased frequency of neutrophil rTEM (Figures 1A–1D; Movie S1) (Woodfin et al., 2011). Elevated percentages of neutrophil rTEM were also observed in cremaster muscles injected with lipopolysaccharide (LPS) (26.4% of all TEM events, n = 6 mice, p = 0.040), identifying local endotoxemia as another inflammatory reaction in which neutrophil rTEM can occur. Since I-R-induced neutrophil rTEM was closely linked with loss of JAM-C from venular EC junctions (Figures 1A and 1E), to determine the mediator(s) that caused this effect, dissected control and I-R stimulated tissues were analyzed by protein array and ELISA. This approach identified numerous inflammatory mediators generated locally in response to I-R, including a number of cytokines (e.g., IL-1β, IL-1α, IL-6), chemokines (MIP-2, KC), C5a, and the lipid mediator LTB_4 (Figure S1A; Figures 1F and 1G). To investigate the impact of these mediators on expression of EC JAM-C in vivo, we injected the stimuli locally into mouse ear skin or cremaster muscles and analyzed the tissues by immunofluorescent staining and confocal microscopy. Based on preliminary studies, a number of these stimuli were analyzed in detail. These included the neutrophil chemoattractants C5a, MIP-2, KC, and LTB_4, mediators that were tested at doses that induced comparable neutrophil infiltration (Figure 1H). While intradermal C5a, MIP-2 and KC had no impact on EC JAM-C expression in skin, LTB_4 reduced JAM-C expression on post-capillary venules (Figures 1H and 1I). LTB_4, but not KC, also reduced the expression of EC JAM-C in cremasteric venules (Figure 1J). Locally administered LTB_4 had no impact on protein expression or localization of other key EC junctional molecules such as PECAM-1, VE-cadherin, or JAM-A (Figure 1H; Figures S1B and S1C). The functional importance of endogenously generated LTB_4 in the cremaster I-R model was confirmed using the LTB_4 BLT1 receptor antagonist LY293111. Animals pre-treated systemically (intravenously, i.v.) with LY293111 showed significant reduction of local neutrophil infiltration (Figure 1K) but were totally protected from loss of EC JAM-C following I-R (Figure 1L). Collectively, these results show that in response to mouse cremasteric I-R, reduced expression of EC JAM-C is accounted for by endogenous LTB_4 and that exogenous LTB_4 is efficacious at causing loss of EC JAM-C. Furthermore, in agreement with our model that reduced functionality of EC JAM-C drives neutrophil rTEM, at doses that induced comparable neutrophil infiltration, local LTB_4 but not KC promoted neutrophil rTEM (Figure 1M; Movie S2).

(B) Quantification of transmigrated neutrophils (n = 3–6) and (C) frequency of neutrophil rTEM events (77–72 events, n = 3–7 mice) through mouse cremasteric venules of tissues subjected to I-R or sham operated, involving 14 independent experiments.

(D) Time-lapse images of a movie (Movie S1) tracking the rTEM response of a GFP-labeled neutrophil (green) through a venule labeled with anti-PECAM-1 mAb (red) following cremaster I-R. On the left are video micrographs of a venular segment imaged from the luminal side that show a neutrophil (arrow head) in the sub-EC space (t = 2 min) that subsequently re-enters the vascular lumen (from 5 min onward) and eventually crawls back into the circulation (13 min). Right side panels show corresponding transverse sections of selected images.

(E) Quantification of junctional EC JAM-C expression acquired from images as shown in (A) (n = 3–6). Images are representative of three independent experiments.

(F and G) Inflammatory mediators in homogenated sham and I-R stimulated cremasters as measured by protein array (F) or ELISA (G) (n = 3).

(H) Confocal images of mouse ear skin injected locally (4 hr, i.d.) with the indicated stimuli and immunostained for MRP14, VE-cadherin, and JAM-C. Images are representative of five independent experiments.

(I and J) Quantification of junctional EC JAM-C expression in ears (I, from images as in H) or cremasters (J) of control tissues or tissues injected locally with the indicated stimuli, as analyzed by confocal microscopy (n = 4–6) from five independent experiments.

(K and L) Quantification of transmigrated neutrophils (K) and junctional EC JAM-C expression (L), as analyzed by confocal microscopy, in sham operated controls or I-R injured cremasters in mice pretreated with vehicle or LY293111 (n = 3–5) involving three independent experiments.

(M) Frequency of neutrophil rTEM events in cremaster muscles injected locally with saline, LTB_4 or KC (n = 73-129 events, 3–9 mice) involving 15 independent experiments. Data indicate mean ± SEM or percentage of total events ± SEM (C and M). *$p < 0.05$, **$p < 0.01$ and ***$p < 0.001$ as compared to controls and $^{\#}p < 0.05$, $^{\#\#}p < 0.01$, $^{\#\#\#}p < 0.001$ as indicated by lines. Scale bars represent 20 μm (A and H) and 10 μm (D). See also Figure S1 and Movies S1 and S2.

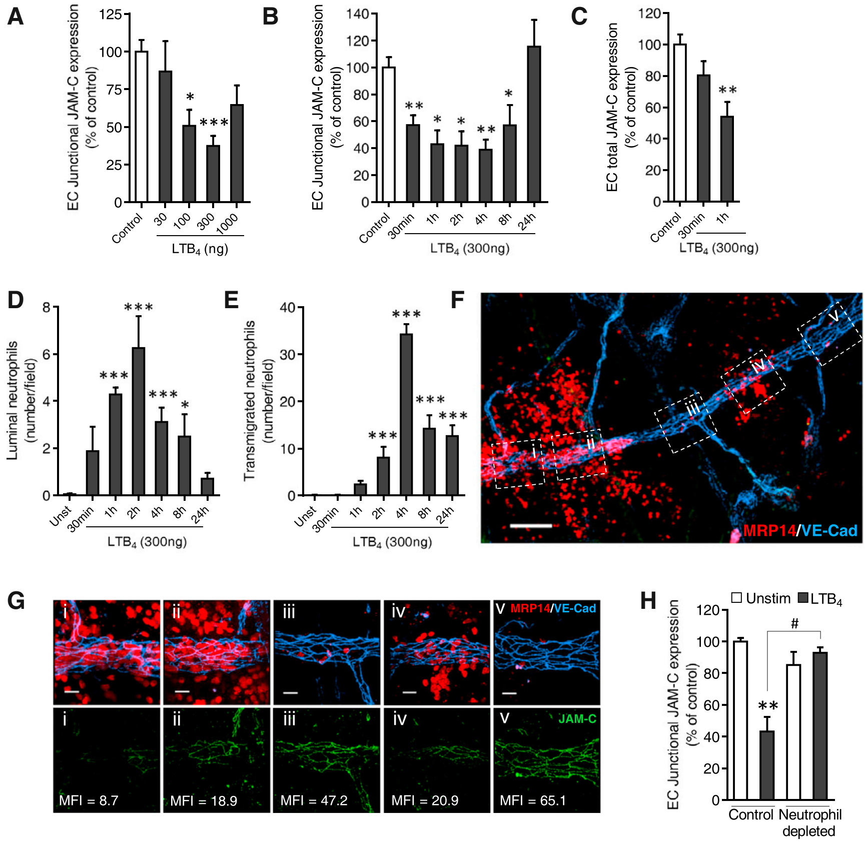

Figure 2. LTB_4-Induced Loss of EC JAM-C Is Neutrophil Dependent

(A) Dose response of LTB_4-mediated reduced expression of EC JAM-C in ear skin of WT mice as analyzed by confocal microscopy (n = 4) from eight independent experiments.

(B–E) Time course of LTB_4-mediated reduced expression of junctional (B) or total (C) EC JAM-C, neutrophil adhesion (luminal) (D), and neutrophil transmigration (E) in ears, as analyzed by confocal microscopy (n = 4–8) involving 23 independent experiments.

(F and G) Confocal images depicting "hot spots" of neutrophil ($MRP14^+$) transmigration in microvessels (VE-cadherin) of LTB_4-stimulated ears. (G) High magnification of the labeled (i–v) venular segments indicated in (F). The lower panels indicate the associated mean fluorescence intensities (MFI) of EC JAM-C junctional expression.

(H) Quantification of junctional EC JAM-C expression in unstimulated or LTB_4-stimulated ears in control and neutrophil-depleted mice (n = 3–7) involving four independent experiments. Data indicate mean ± SEM. *$p < 0.05$, **$p < 0.01$ and ***$p < 0.001$, as compared to controls and $^{\#}p < 0.05$ as indicated by lines. Scale bars represent 100 μm (F) and 20 μm (G). See also Figure S2.

LTB_4-Induced Loss of EC JAM-C Is Neutrophil Dependent

Loss of EC JAM-C caused by locally administered LTB_4 occurred in a dose- and time-dependent manner (Figures 2A and 2B). Significant reduction in expression of EC JAM-C at EC junctions was noted as early as 30 mins post local application of LTB_4 (Figure 2B). This was sustained for up to 8 hr and returned to normal by 24 hr (Figure 2B). Of note, total expression of venular EC JAM-C (i.e., including junctional and cell body expression) was also significantly reduced post administration of LTB_4, but with a slight temporal delay as compared to junctional JAM-C (Figures 2B and 2C). These results suggest that EC JAM-C is initially re-localized from junctions to non-junctional regions (e.g., plasma membrane) following LTB_4 stimulation, in agreement with our findings using the mouse cremaster I-R model (Scheiermann et al., 2009). This response might then be followed by total loss of the protein at later time points.

The time-course of LTB_4-induced loss of venular JAM-C was directly aligned with time-course of LTB_4-elicited neutrophil attachment to and migration through venular walls (Figures 2D and 2E). In addition, while JAM-C was expressed in all types of microvessels (capillaries > venules > arteries in ear skin and cremaster muscle; Figure S2), in LTB_4-stimulated tissues, reduced expression of the molecule was selectively noted in post-capillary venules, the primary sites of neutrophil transmigration. Indeed, in tissues stimulated with locally administered LTB_4, reduced expression of EC JAM-C was a feature of venular segments supporting intense neutrophil transmigration ("hot-spots") (Figures 2F and 2G). Collectively, these results suggested a role for neutrophils in loss of EC JAM-C. Direct evidence for this was obtained through the use of mice depleted of their circulating neutrophils in which LTB_4-injected tissues (skin) showed normal expression of venular JAM-C (Figure 2H), contrary to the reduced amounts noted in LTB_4-stimulated control mice. These results indicate that loss of EC JAM-C following LTB_4 stimulation is a neutrophil-dependent phenomenon and implied proteolytic cleavage of the protein as a possible mechanism.

Neutrophil Elastase Cleaves EC JAM-C

Because LTB_4 is a potent inducer of NE release and/or cell-surface expression (Rainger et al., 1998; Young et al., 2007), we considered that this serine protease might cleave EC JAM-C in response to LTB_4. Initial in vitro studies using an anti-NE mAb and an NE-fluorescent activatable substrate (NE680FAST) illustrated the potent ability of LTB_4 to rapidly mobilize intracellular stores of NE protein and to increase NE activity, respectively, to the neutrophil cell surface (Figures 3A–3C). Such effects were not noted with C5a or KC. Importantly, mobilization of neutrophil intracellular stores of NE protein and intense neutrophil-associated NE activity could also be observed in vivo in LTB_4-stimulated tissues as compared to tissues stimulated by other inflammatory mediators (Figures 3D and 3E). The use of *Elane*$^{-/-}$ ($NE^{-/-}$) mice or the NE inhibitor GW311616A provided conclusive in vivo evidence for the involvement of NE in JAM-C cleavage (Figure 3F; Figure S3). Furthermore, purified NE cleaved recombinant soluble JAM-C in vitro, as shown by immunoblot (Figure 3G). Collectively, these results demonstrate that neutrophil-derived NE is responsible for loss of EC JAM-C as induced following rapid mobilization of the enzyme to the neutrophil cell surface by LTB_4.

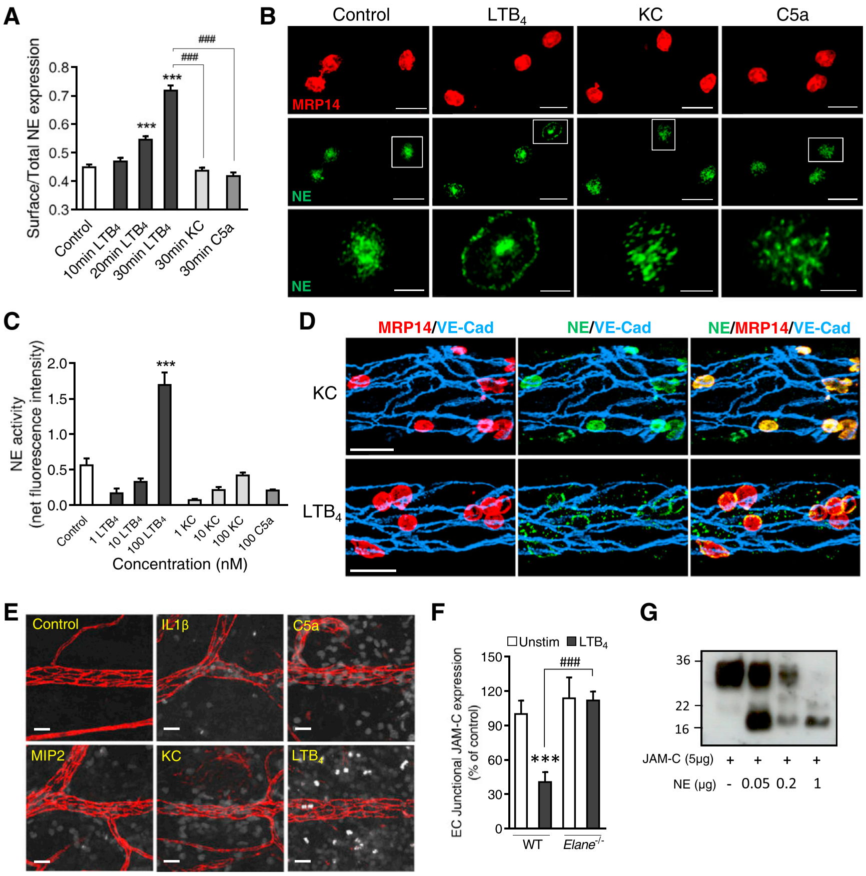

Figure 3. Neutrophil Elastase Mediates Cleavage of EC JAM-C

(A–C) Effect of LTB_4, as compared to other indicated stimuli, on cell surface NE protein expression (using an anti-NE mAb) (A and B, n = 32–102 cells) and NE activity (C, n = 23–166 cells) of BM neutrophils adherent to BSA-coated slides. Data are representative of three independent experiments.

(D) Confocal images of stimulated cremaster muscles immunofluorescently stained for NE, neutrophils (MRP14), and VE-cadherin showing re-distribution of NE to the cell surface following 1 hr tissue stimulation with LTB_4 but not KC (n = 2 mice per group). Images are representative of two independent experiments.

(E) Confocal images showing NE activity (white) in ears injected intradermally with the indicated stimuli. Venules are shown in red (VE-cadherin) (n = 3 mice per group). Images are representative of two independent experiments.

(F) Quantification of EC JAM-C expression in control and LTB_4-stimulated ears of WT and *Elane*$^{-/-}$ mice (n = 5–9) from four independent experiments.

(G) Immunoblot against JAM-C shows the ability of purified NE to cleave recombinant JAM-C (representative of three independent experiments). Data indicate mean ± SEM. ***$p < 0.001$, as compared to control and $^{###}p < 0.001$ as indicated by lines. Scale bars represent 5 μm (B, bottom panels) and 20 μm (B, upper panels; D and E). See also Figure S3.

Binding of NE to Mac-1 Supports Cleavage of JAM-C

We next sought to investigate the impact of exogenously administered NE on EC JAM-C in vivo. In contrast to the in vitro ability of NE to cleave JAM-C, local injection of NE into cremaster muscles failed to impact the expression of EC JAM-C (Figure 4A). This discrepancy was considered to be due to an essential need for neutrophils in vivo, because exogenous NE failed to significantly induce neutrophil transmigration (Figure 4B). Specifically, we hypothesized that neutrophils might be required to present NE to JAM-C on ECs in order for NE to cleave this adhesion molecule. To explore this possibility, we co-injected NE with KC, a chemokine that induced significant neutrophil infiltration but at the dose tested had no impact on EC JAM-C expression (Figures 4A and 4B). Cremaster muscles co-injected locally with NE and KC showed similar neutrophil infiltration to that noted with KC alone (Figure 4B). However, in contrast to tissues injected with KC or NE alone, tissues stimulated with NE+KC exhibited a significant reduction in EC JAM-C expression (Figure 4A). Because NE has previously been reported to bind to the neutrophil integrin Mac-1 (Cai and Wright, 1996) and Mac-1 is a ligand for JAM-C (Santoso et al., 2002), we considered that the underlying reason for the efficacy of LTB_4 to induce cleavage of JAM-C might be due to the ability of the lipid to be both an effective activator of Mac-1 and inducer of NE release. In support of this, mouse neutrophils stimulated with LTB_4 or KC adhered to ICAM-1-coated slides in a Mac-1-dependent manner (Figure 4C), demonstrating activation of the integrin by these neutrophil chemoattractants. Under these in vitro conditions, however, at concentrations that caused comparable adhesion to ICAM-1-coated slides, only LTB_4 was capable of eliciting release of NE (Figure 4D). The functional implication of this in relation to JAM-C cleavage was investigated in an assay in which the interaction of neutrophils with JAM-C and ICAM-1-coated slides was analyzed by fluorescent microscopy and JAM-C cleavage was quantified through immunofluorescence detection of JAM-C (Figures 4E–4H). Stimulation of cells with LTB_4 or KC again induced comparable neutrophil adhesion to the coated slides (as found with ICAM-1-coated slides), but only LTB_4 stimulation significantly increased cleavage of JAM-C as compared to unstimulated control cells (Figures 4E–4I). The latter response was NE dependent, as assessed using an NE inhibitor (GW311616A) (Figure 4I). This assay was next extended to explore the possibility that direct interaction of Mac-1 with NE is necessary for cleavage of JAM-C. For this purpose, *Elane*$^{-/-}$ neutrophils were pre-treated with or without anti-Mac-1 mAb followed by LTB_4 stimulation in the presence of exogenous NE (Figure 4J). The resulting cell samples were then washed and placed onto JAM-C- and ICAM-1-coated slides, and after 30 min the slides were fixed and stained for JAM-C expression. In this model, LTB_4-stimulated *Elane*$^{-/-}$ cells supplemented with exogenous NE led to JAM-C cleavage, and this response was totally inhibited under conditions of Mac-1 blockade (Figure 4K). Finally, co-immunoprecipitation experiments showed that Mac-1 from LTB_4-stimulated (but not control unstimulated)

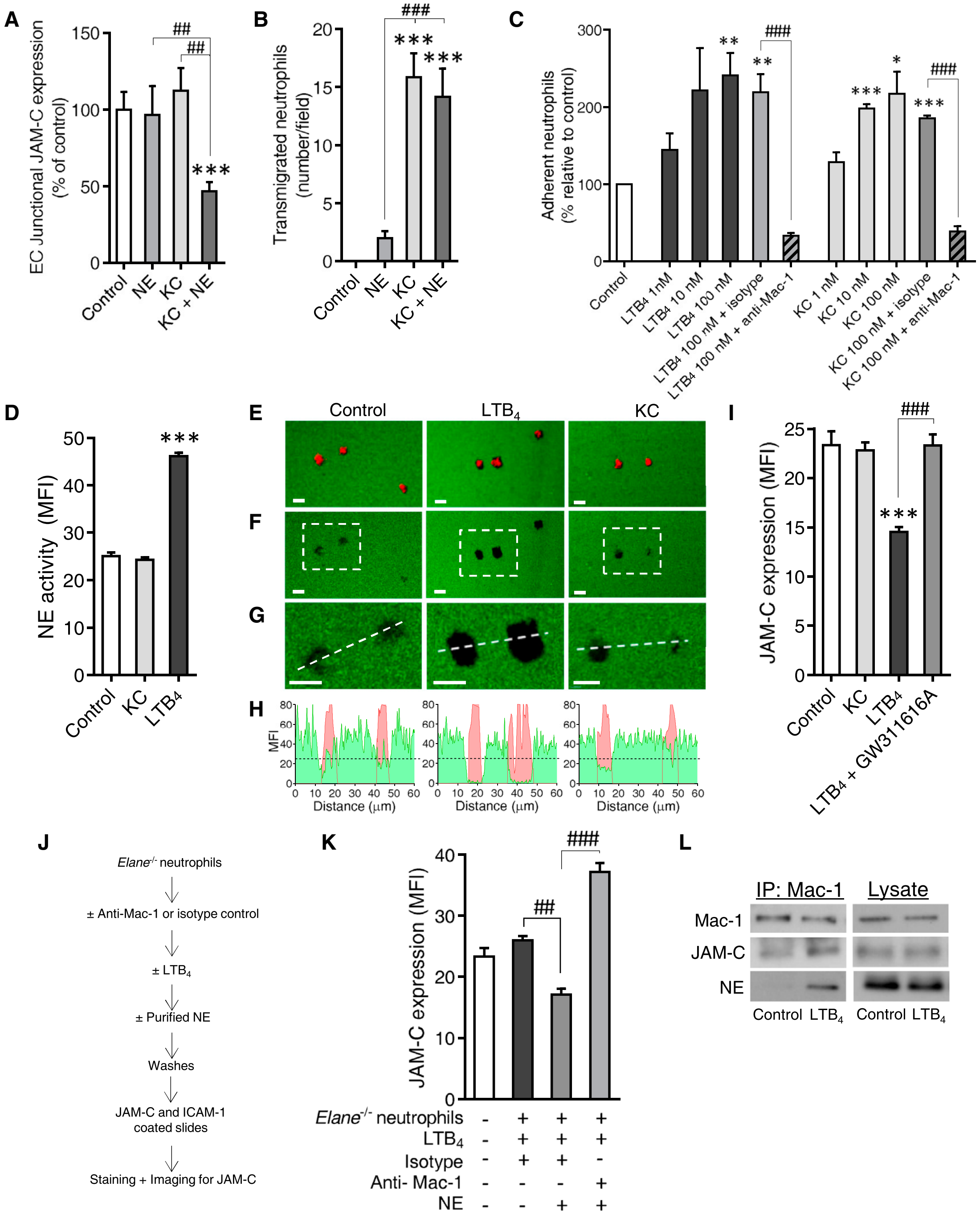

Figure 4. NE Bound to Mac-1 Mediates Cleavage of JAM-C

(A and B) Quantification of EC JAM-C expression (A) and number of transmigrated neutrophils (B) in cremaster muscles locally injected (4 hr, i.s.) with the indicated stimuli (n = 3–6) involving four independent experiments.

(C and D) Neutrophil adhesion to ICAM-1-coated slides (C) (n = 3 experiments) and NE activity (D) (n = 5 experiments) as induced by LTB_4 and KC.

(E–H) Unstimulated (control), LTB_4- and KC-stimulated neutrophils (MRP-14; red) adherent to slides co-coated with ICAM-1 and JAM-C (green) (E) and associated regions of JAM-C loss (F). Bottom panels (G) show high-magnification images of the boxed regions in (F) with the associated linear intensity profiles (H) of the neutrophil (red) and JAM-C (green) channels along the white dotted lines (G). Black dotted lines in (H) indicate average expression of JAM-C at sites of neutrophil adhesion in control unstimulated samples.

(I) Quantification of JAM-C at sites of neutrophil adhesion as illustrated in (E)–(H) (n = 4 experiments).

(J and K) Protocol (J) used for quantification (K) of JAM-C expression post adhesion of LTB_4-stimulated *Elane*$^{-/-}$ neutrophils to JAM-C- and ICAM-1-coated slides (n = 3 experiments).

(L) Co-immunoprecipitation and immunoblot analysis of untreated and LTB_4-stimulated neutrophils (representative of two independent experiments). Data indicate mean ± SEM. $^{*}p < 0.05$, $^{**}p < 0.01$ and $^{***}p < 0.001$ as compared to controls and $^{\#\#}p < 0.01$, $^{\#\#\#}p < 0.001$ as indicated by lines. Scale bars represent 10 μm.

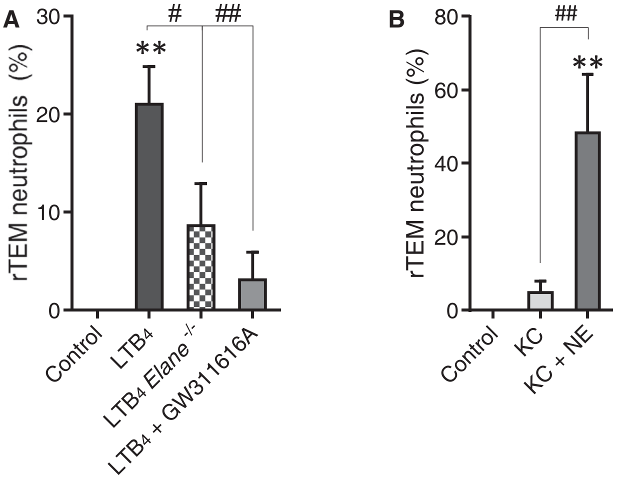

Figure 5. LTB_4 and NE Promote Neutrophil rTEM

Frequency of neutrophil rTEM events through cremasteric venules of tissues from WT mice injected locally with saline (control) or LTB_4 with or without pre-treatment with GW311616A and in Elane$^{-/-}$ mice injected with LTB_4 (A) and in tissues injected locally with KC or KC+NE (B) (19–217 events, 3–9 mice) involving 22 independent experiments. Data indicate percentage of total events ± SEM. $^{**}p < 0.01$ as compared to controls and $^{\#}p < 0.05$ and $^{\#\#}p < 0.01$ as indicated by lines.

mouse neutrophils binds to both NE and JAM-C (Figure 4L). Collectively, these results further illustrated the importance of NE in JAM-C cleavage and demonstrated that NE bound to neutrophil Mac-1 supported this response.

Local LTB_4-NE Axis Promotes Neutrophil rTEM

Because LTB_4 and NE were identified as key molecular players in inducing loss of EC JAM-C and we have previously shown that reduced expression of EC JAM-C drives neutrophil rTEM (Woodfin et al., 2011), we next explored the role of the LTB_4-NE axis in induction of neutrophil rTEM. LTB_4-induced neutrophil rTEM was significantly suppressed in *Elane*$^{-/-}$ mice and in wild-type (WT) mice pre-treated with an NE inhibitor (Figure 5A). In addition, because KC, a mediator that did not impact the expression of EC JAM-C (Figure 1H–1J), also failed to elicit a notable frequency of neutrophil rTEM (Figure 1M), the effect of the chemokine when co-injected with NE into the cremaster muscle (a reaction that caused local loss of EC JAM-C; Figure 4A) was tested. This reaction led to profound frequency of neutrophil rTEM as compared to responses detected in mice injected with KC alone (Figure 5B). Collectively, the results demonstrate that locally activated LTB_4-NE axis is highly effective at promoting neutrophil rTEM.

Activation of LTB_4-NE Axis Drives a Local Inflammatory Response to Become Systemic

We have previously found an association between loss of EC JAM-C expression and/or functionality, neutrophil rTEM, and distant organ (lung) inflammation (Woodfin et al., 2011). Having identified LTB_4 as an effective stimulus that triggers NE-mediated loss of EC JAM-C and promoted neutrophil rTEM, we investigated a possible causal relationship between this cascade of events and secondary organ inflammation. In addition to stimulating local neutrophil infiltration (Figure 6A), LTB_4 injected into the mouse cremaster muscle elicited neutrophil accumulation into lungs (Figure 6B) and tissue damage (plasma protein leakage) in multiple organs, e.g., lung, heart, and liver (Figure 6C). Time-course studies illustrated that, after injection of LTB_4 into the mouse cremaster muscle, lung injury peaked at 4 hr and returned to normal by 24 hr (Figure S4A). A similar transient profile of lung injury was found in other tissues such as the heart (Figure S4B). Similarly to the cremaster muscle, intradermal injection of LTB_4 into the ear skin promoted lung neutrophil accumulation (Figure S4C) and remote organ damage (Figure S4D). These effects were attenuated in *Elane*$^{-/-}$ mice or in WT mice pretreated with the NE inhibitor GW311616A (Figures 6B–6D; Figure S4D), without affecting local LTB_4-induced neutrophil recruitment (Figure 6A). Of note, *Elane*$^{-/-}$ mice and WT mice pretreated with GW311616A exhibited normal neutrophil migration into lungs in response to intranasal instillation of LTB_4 (Figure S4E), indicating that NE blockade does not directly suppress neutrophil infiltration into lungs. Together these results show that LTB_4, when administered into a primary tissue site, can promote secondary organ damage in an NE-dependent manner. Importantly, we also noted increased remote organ (lung and heart) damage following cremaster I-R injury, a response that was inhibited in *Elane*$^{-/-}$ mice (Figure S4F).

Because NE was found to be essential in regulating LTB_4-induced local EC JAM-C expression (Figure 3F; Figure S3), a potential link between local loss of EC JAM-C and secondary organ inflammation was further investigated. For this purpose, we analyzed the effects of locally administered KC, a neutrophil chemoattractant that did not cause loss of EC JAM-C (Figures 1H–1J). When applied locally to the cremaster muscle at a dose that elicited significant local neutrophil infiltration (Figure 4B), KC did not cause neutrophil recruitment into lungs (Figure 6E). In contrast, while the combined injection of KC and NE into the cremaster muscle induced comparable neutrophil infiltration to that observed with KC alone (Figure 4B), KC+NE led to significant neutrophil accumulation into lungs, as compared to responses detected in mice treated with KC or NE alone (Figure 6E). Because we have previously shown that KC+NE can cause local loss of EC JAM-C and promote neutrophil rTEM (Figure 4A and 5B), these results provide strong evidence to suggest that local regulation of EC JAM-C is instrumental in promoting distant organ damage. In support of this, we noted that KC, when injected into the cremaster muscle of mice with specific deletion of EC JAM-C (*Tekcre;Jam-3*$^{flox/flox}$), caused significant lung edema as compared to responses observed in littermate controls (Figure 6F). Collectively, through loss-of-function and gain-of-function studies, these results demonstrate that inflammatory reactions that can mediate NE-induced local loss of EC JAM-C can promote development of secondary multi-organ inflammation.

Soluble JAM-C Is Detected in Plasma of Locally Inflamed Mice and in Trauma Patients

As a consequence of local proteolytic cleavage by NE, we speculated that soluble JAM-C (sJAM-C) might be detectable in plasma of mice after tissue stimulation. The low circulating sJAM-C detected in control mice was significantly increased following intradermal injection of LTB_4 (Figure 7A), a parameter that inversely correlated with the loss of venular EC JAM-C ($p = 0.0041$). This response was abrogated in *Elane*$^{-/-}$ mice and in

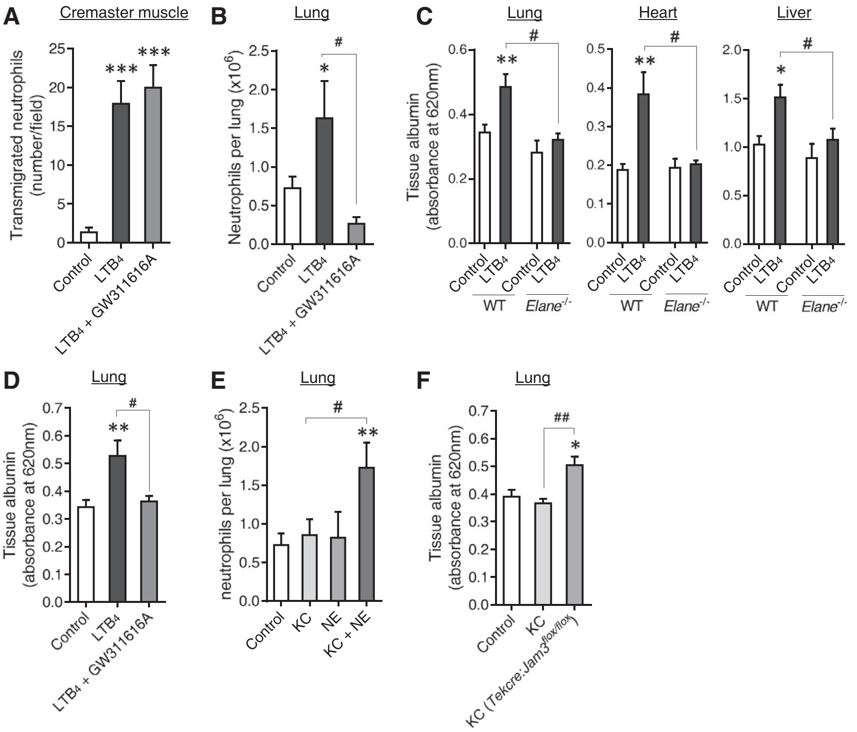

Figure 6. LTB_4 and NE Promote Distant Organ Damage

(A) Quantification of transmigrated neutrophils into mouse cremaster muscles in unstimulated tissues (control) or 4 hr post local injection of LTB_4 with or without pretreatment (24 hr) of mice with GW311616A (n = 4–7) from six independent experiments.

(B) Quantification of lung neutrophil numbers (n = 4–15) following cremaster stimulation (as in A) involving 15 independent experiments.

(C) Quantification of lung, heart, and liver albumin content (plasma extravasation), as a marker of tissue damage, following local (cremaster) injection of LTB_4 (4 hr) as compared to control unstimulated tissues in WT and *Elane*$^{-/-}$ mice (n = 4–24) involving 12 independent experiments.

(D) Lung albumin content in unstimulated tissues (control) or post local (cremaster) injection of LTB_4 with or without pretreatment (24 hr) of mice with GW311616A (n = 4–24) involving 16 independent experiments.

(E and F) Lung neutrophil numbers (E) (n = 6–15) and tissue albumin content (F) (n = 3–5) following local injection of KC, NE, or KC+NE in cremaster muscles of WT or *Tekcre;Jam-3*$^{flox/flox}$ mice involving 21 independent experiments. Data indicate mean ± SEM. $^*p < 0.05$, $^{**}p < 0.01$ and $^{***}p < 0.001$ as compared to controls and $^{\#}p < 0.05$ and $^{\#\#}p < 0.01$ as indicated by lines. See also Figure S4.

mice depleted of their circulating neutrophils (Figure 7A), illustrating that as found with local loss of venular EC JAM-C, presence of sJAM-C in plasma is both neutrophil- and NE-dependent. Importantly, elevated sJAM-C was also found in plasma samples from trauma patients as compared to control volunteers (Figure 7B). Patients who developed acute respiratory distress syndrome (ARDS) exhibited higher amounts (>50%) of plasma sJAM-C on admission than those who maintained normal lung function (Figure 7B). Furthermore, there was a significant correlation between on admission plasma content of sJAM-C and the subsequent severity of multi-organ failure (MOF) as assessed by their Sequential Organ Failure Assessment (SOFA) scores at 48 hr after injury (Figure 7C). Thus, in conjunction with our previous findings, there is an association between increased amounts of sJAM-C in plasma and distant organ inflammation following local LTB_4 in mice and importantly post tissue injury in humans.

DISCUSSION

Here we investigated the mechanism through which neutrophil rTEM is triggered in vivo. The results identified the lipid neutrophil chemoattractant LTB_4 as a key mediator capable of promoting proteolytic cleavage of EC JAM-C by NE, a response that drives neutrophil rTEM. The activation of this cascade of events was also associated with dissemination of systemic inflammation, suggesting that targeting LTB_4-NE axis might be an efficacious means of suppressing secondary organ damage following local inflammation or injury.

While neutrophils play a key role in mounting an early innate immune response and have also been intimately associated with the development of acute inflammatory disorders, there is now ample evidence for a broader role for neutrophils in inflammation and immunity (Mantovani et al., 2011; Mócsai, 2013; Mayadas et al., 2014). Most notably, neutrophils are known to interact with components of the adaptive immune response and have been implicated in the pathogenesis of numerous chronic inflammatory conditions (Mantovani et al., 2011; Mócsai, 2013; Mayadas et al., 2014). Furthermore, the traditional view of neutrophils exhibiting a short half-life (i.e., < several hours) has been challenged (Tak et al., 2013), and there is evidence for the existence of neutrophil "subsets" exhibiting a wide range of phenotypes in different physiological and pathological conditions (Beyrau et al., 2012; Kolaczkowska and Kubes, 2013). In addition, while conventionally neutrophils have been considered as cells that move in a one-way direction, there is now evidence for the ability of neutrophils to breach ECs in an abluminal-to-luminal direction, i.e., exhibit reverse TEM (Buckley et al., 2006; Mathias et al., 2006; Woodfin et al., 2011), though our understanding of the prevalence, regulation, and implications of this response are at an early stage.

Through the application of confocal IVM to analysis of inflamed mouse cremaster muscles, we recently reported significant frequency of neutrophil rTEM following I-R (Woodfin et al., 2011). Mechanistically, rTEM was associated with and indeed promoted by reduced expression and/or function of EC JAM-C (Woodfin et al., 2011). To gain a better understanding of the inflammatory trigger(s) that promote neutrophil rTEM, we sought to identify the mechanism(s) through which EC JAM-C is lost. Analysis of mouse cremaster muscles subjected to I-R detected an array of endogenously generated mediators, in line with the network of mediators identified in other models of I-R (Iadecola and Anrather, 2011; Smith et al., 2012). Among these, LTB_4 was identified as a mediator that effectively reduced expression of EC JAM-C in vivo, and through the use of a specific BLT1

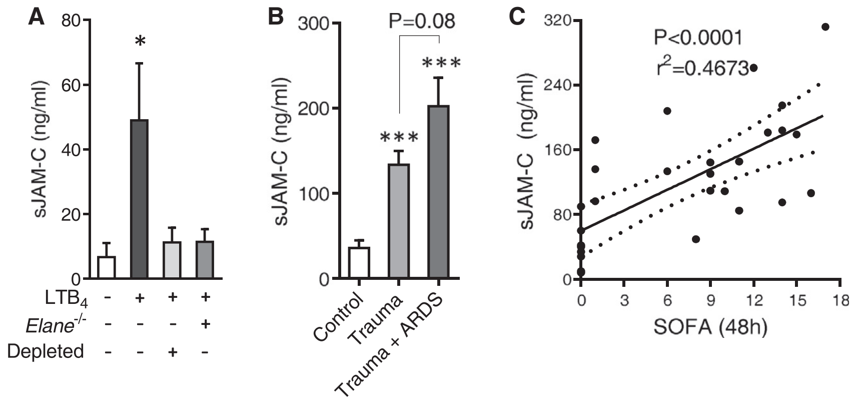

Figure 7. Locally Inflamed Mice and Trauma Patients Exhibit Increased Plasma Content of Soluble JAM-C

(A) Plasma sJAM-C in WT, *Elane*$^{-/-}$, and neutrophil depleted mice under basal conditions (control) and following intradermal injection (ear, i.d. 4 hr) of LTB_4 (n = 4–16) involving 11 independent experiments.

(B) Plasma content of sJAM-C in healthy control volunteers and trauma patients with or without ARDS (n = 8–10).

(C) Correlation of plasma sJAM-C with SOFA scores in individuals from (B). Data indicate mean ± SEM. *p < 0.05 and ***p < 0.001 as compared to controls. Additional statistical analyses are indicated.

receptor antagonist, endogenous LTB_4 was found to be totally accountable for loss of EC JAM-C expression in response to I-R. In agreement with investigations of other disease models (Souza et al., 2000; Kim et al., 2006; Chou et al., 2010), LTB_4 also contributed to local neutrophil infiltration as elicited by I-R. An important cellular source of this LTB_4 is likely to be the neutrophils themselves (Chen et al., 2006; Afonso et al., 2012; Lämmermann et al., 2013). The potent ability of locally administered LTB_4 to cause loss of EC JAM-C was associated with the ability of this mediator to induce neutrophil rTEM. These results are directly in line with our previous findings (Woodfin et al., 2011) and the overall hypothesis that loss of EC JAM-C is instrumental in mediating neutrophil rTEM.

The effect of LTB_4 was selective to JAM-C in that it had no impact on expression of other key EC adhesion molecules. We have previously reported that in response to I-R, EC JAM-C is re-distributed from junctions and intracellular cytoplasmic vesicles toward non-junctional plasma membrane (Scheiermann et al., 2009). Similarly, our present findings suggest an initial redistribution of the molecule away from EC junctions following LTB_4 stimulation, a response that could support adhesion of neutrophils to the endothelium (Scheiermann et al., 2009). Reduction of both junctional and total JAM-C expression at later time points suggested that the protein was also cleaved from ECs in this reaction, and multiple lines of evidence indicated that this was a neutrophil-dependent response. This included the following: (1) The response occurred largely in post-capillary venules, the primary site of neutrophil transmigration, (2) the time-course of the response was directly aligned with the time-course of neutrophil adhesion and transmigration, (3) EC JAM-C loss was most notable at sites of intense neutrophil transmigration (“hot-spots”), and finally, (4) LTB_4-induced loss of EC JAM-C was abolished in neutrophil-depleted mice. In investigating the mechanism through which neutrophils caused reduced expression of EC JAM-C, we focused our attention on the possible role of NE. This serine protease is expressed within neutrophil azurophil granules and exhibits a broad substrate specificity, including numerous cell-surface receptors and adhesion molecules (Pham, 2006). Because LTB_4 is highly efficacious at inducing NE degranulation from mouse neutrophils in vitro (Young et al., 2007), we hypothesized that NE might mediate neutrophil-dependent loss of EC JAM-C. In support of this, LTB_4 showed high efficacy in promoting rapid NE release and/or cell-surface expression and activity (as mobilized from intracellular stores), both in vitro and in vivo, as compared to other neutrophil chemoattractants. Furthermore, we have recently demonstrated that local LTB_4 can rapidly activate neutrophils within the vascular lumen when the neutrophils are in close apposition to the venular wall and junctions (Finsterbusch et al., 2014). Direct evidence for the role of NE in mediating LTB_4-induced loss of venular JAM-C was provided with the use of NE-deficient mice or mice pre-treated with an NE inhibitor. Together, our findings identify the potent ability of LTB_4 to induce mobilization of NE to the neutrophil cell surface as a key factor in the ability of this lipid mediator to cause loss of JAM-C at sites of inflammation.

Activation of the neutrophil BLT1 receptor is efficiently linked to signaling pathways that trigger degranulation of azurophil granules containing NE (Rainger et al., 1998). This could be of physiological benefit when LTB_4 is generated and localized to the interstitial tissue. Here it can support clearance of pathogens or act as a signal-relay molecule for neutrophil chemotaxis or neutrophil swarming to sites of injury, responses that have been linked to NE and LTB_4, respectively (Belaaouaj et al., 1998; Afonso et al., 2012; Lämmermann et al., 2013). In contrast, under pathological conditions, such as that encountered following I-R injury, excessive generation of LTB_4 might activate neutrophils within the vascular lumen, leading to inappropriate and damaging release of NE at the vessel wall. Based on our findings and proposed model, the latter could lead to cleavage of EC JAM-C and development of rTEM. To achieve this effect, we hypothesized that following its release from azurophil granules, neutrophil surface-bound NE is presented to EC JAM-C during TEM. The concept that serine proteases remain cell bound after fusion of granules with the plasma membrane is well recognized and is considered as a means through which the enzymes are protected from endogenous interstitial protease inhibitors (Owen and Campbell, 1999). What is less clear, however, is the mechanism through which serine proteases bind to the neutrophil cell surface. In this context while charge-dependent mechanisms have been proposed (Campbell and Owen, 2007), NE has also been shown to interact with neutrophil Mac-1 (Cai and Wright, 1996). Building on these findings and the fact that Mac-1 has been identified as a ligand for JAM-C (Santoso et al., 2002), we extended our hypothesis to a potential role for an LTB_4-NE-Mac-1 axis in cleavage of JAM-C. Using a number of in vitro models, our findings demonstrated that although both LTB_4 and KC could effectively induce Mac-1

activation, the efficacy of LTB_4 to induce JAM-C cleavage resides in the capacity of this mediator to induce NE expression to the neutrophil cell surface. Taking advantage of NE deficient neutrophils, evidence was obtained for the ability of NE to bind to activated Mac-1 upon LTB_4 stimulation, an interaction that facilitated JAM-C cleavage. Importantly, rTEM caused by LTB_4 was NE-dependent, and co-injection of exogenous NE with KC (at doses that individually did not cause neutrophil rTEM) induced profound rTEM, demonstrating that the activation of the LTB_4-NE axis (through loss-of-function or gain-of-function studies) can lead to loss of EC JAM-C and promotion of neutrophil rTEM. Collectively, these findings provide support for the overall hypothesis that NE is presented to EC JAM-C via activated Mac-1, the latter acting as a molecular "bridge" between NE and JAM-C. This provides a mechanism through which JAM-C is cleaved in a selective manner, thus promoting neutrophil rTEM.

At present, the functional implication of neutrophils undergoing reverse TEM is unclear. It is potentially possible that this phenomenon might have a physiological role such as dampening down a local inflammatory response (Mathias et al., 2006; Yoo and Huttenlocher, 2011) and/or reflect a role for neutrophils as cellular sentinels or sirens of inflammation. Alternatively, neutrophil rTEM might play a pathological role such as contributing to turning a local acute inflammatory response into a systemic phenomenon (Woodfin et al., 2011). Although further studies are required, the results of the present study support the latter. Most notably, local I-R injury or local administration of LTB_4 (cremaster muscle or ear skin) caused remote organ damage. Furthermore, all pharmacological or genetic interventions that suppressed or enhanced neutrophil rTEM (e.g., *Elane*$^{-/-}$ mice and NE+KC), correspondingly regulated second organ inflammation. Because neutrophils that have undergone rTEM have been shown to exhibit a pro-inflammatory state (Buckley et al., 2006; Woodfin et al., 2011), the present findings support the paradigm that reverse TEM results in re-entry of a small subset of activated neutrophils into the blood circulation that can contribute to turning a local inflammatory response into a systemic phenomenon. The findings further indicated that NE inhibition had no impact on local neutrophil infiltration into the cremaster muscle, suggesting that although NE is not essential in mediating local neutrophil infiltration, excessive and/or inappropriately generated local NE can promote neutrophil rTEM and remote organ damage.

As a consequence of EC JAM-C cleavage by NE, increased amounts of circulating sJAM-C were found in mice locally stimulated with LTB_4. Elevated concentration of sJAM-C was also found in plasma from trauma patients as compared to healthy controls, a parameter that further increased in patients that developed acute respiratory distress syndrome (ARDS) after admission. ARDS is characterized by severe respiratory failure, and patients with ARDS exhibit ~40% mortality rate (Rubenfeld et al., 2005; Wheeler and Bernard, 2007). Pathophysiology of ARDS is characterized by increased lung permeability and neutrophil infiltration and is typically associated with sepsis and multiple organ failure (MOF) (Matthay and Zemans, 2011). Our findings, together with the reported elevated plasma content of LTB_4, as well as NE activity, found in trauma patients that progress to ARDS (Donnelly et al., 1995; Auner et al., 2012), suggest that similar mechanisms (i.e., LTB_4-dependent NE cleavage of EC JAM-C) could be taking place following trauma. By extension, the results suggest that blocking JAM-C cleavage by inhibition of NE could be a useful preventative strategy in trauma patients at risk of developing ARDS and MOF. Importantly, NE inhibitors are currently in use in Japan in patients with ARDS associated with systemic inflammatory response syndrome (SIRS), as well as for reducing surgery-induced pulmonary inflammation (Fujii et al., 2010; Aikawa et al., 2011). Together, the present study provides previously unknown mechanistic insights into how NE inhibitors might prevent secondary ARDS. Furthermore, because increased plasma content of sJAM-C was associated with trauma-induced organ failure and also elevated in serum or synovial fluid from rheumatoid arthritis, psoriatic arthritis, osteoarthritis, and systemic sclerosis patients (Rabquer et al., 2010; Manetti et al., 2013), we propose that sJAM-C might be a useful vascular-derived biomarker for assessing the extent of a systemic inflammatory response.

In summary, we have identified a role for local LTB_4 and NE as regulators of neutrophil rTEM. Furthermore, although at present the precise functional implications of neutrophils undergoing rTEM remain unclear, the results of this study provide further evidence for this phenomenon being associated with dissemination of inflammation.

EXPERIMENTAL PROCEDURES

Animals

Lyz2-EGFP-ki mice (exhibiting green fluorescent neutrophils), *Elane*$^{-/-}$ mice, *Elane*$^{-/-}$-*Lyz2-EGFP-ki* mice, EC JAM-C$^{-/-}$ mice (*Tekcre;Jam-3*$^{flox/flox}$) and their control littermates (*Jam-3*$^{flox/flox}$), and wild-type C57BL/6 mice were used. All animal experiments were conducted in accordance with the UK Home Office legislations.

Patients

Blood from trauma patients (sampled less than 2 hr after injury) or healthy donor controls was collected in buffered sodium citrate.

Induction of Inflammatory Reactions and Pre-treatments

Inflammatory stimuli such as LTB_4, LPS, CXCL1 (KC), CXCL2 (MIP-2), C5a, or vehicle control were injected in the ears or the cremaster muscles of anesthetized mice. Some mice were pre-treated (24 hr, orally) with the NE inhibitor GW311616A. Cremaster I-R injury (30 min ischemia followed by 2 hr reperfusion) was induced as previously detailed (Scheiermann et al., 2009). In some experiments, the LTB_4 receptor antagonist LY293111 was administered i.v. 15 min prior to I-R.

Whole-Mount Tissue Immunofluorescence Staining

Ears or cremaster muscles were immunostained before being mounted on slides and analyzed by confocal microscopy.

Confocal Microscopy

Immunofluorescently stained cells and whole-mounted tissues were imaged by confocal microscopy and analyzed by IMARIS or ImageJ software.

Quantification of Plasma Soluble JAM-C

sJAM-C plasma content was analyzed by ELISA.

Cytokine and Chemokine Expression Profile

The profile of different inflammatory mediators in mouse cremasters was analyzed using the Mouse Cytokine Array Panel A Array Kit or ELISA kits.

In Vitro Digestion of JAM-C

Purified recombinant murine JAM-C was incubated with purified NE for 1 hr at 37°. Samples were then resolved in a SDS-PAGE gel and visualized by Western blot.

Co-immunoprecipitation

Bone marrow (BM)-derived neutrophils were purified and stimulated with LTB_4 or left unstimulated. Samples were then lysed, incubated overnight with an anti-Mac-1 mAb, and immunoprecipitated. JAM-C and NE protein content was then analyzed by Western blot.

Immunoblot

Samples were resolved on SDS-PAGE gels and electrotransferred onto PVDF membranes. These were then blocked, incubated with primary and HRP-conjugated secondary antibodies, and developed using a Chemoluminescent Substrate.

In Vitro Neutrophil Adhesion Assay

16-well glass chamber slides were coated with recombinant human ICAM-1 and blocked with 10% BSA. BM Neutrophils (5×10^4/well) were added to the chambers and were either left untreated or stimulated with LTB_4. In some experiments, neutrophils were pre-incubated with an anti-Mac-1 mAb. The number of adherent cells was quantified by phase contrast microscopy.

Imaging of JAM-C Cleavage In Vitro

BM neutrophils seeded in 16-well glass chamber slides coated with recombinant ICAM-1 and JAM-C were left untreated or stimulated with LTB_4, LTB_4+ GW311616A or KC for 30 min. Slides were then fixed, immunostained with antibodies against JAM-C and MRP-14, and analyzed by confocal microscopy.

In Vivo NE Enzymatic Activity Assay

NE680FAST was injected i.v. and left to circulate for 15 min before induction of inflammation. At the end of the experiment tissues were collected, immunofluorescently stained, and analyzed by confocal microscopy.

In Vitro NE Enzymatic Activity Assay

BM neutrophils were seeded on ICAM-1 and JAM-C coated slides and left untreated or stimulated for 30 min with KC or LTB_4 in the presence of NE680-FAST. Slides were then washed, fixed, immunostained, and analyzed by confocal microscopy.

Confocal Intravital Microscopy of Mouse Cremaster Muscles

Lyz2-EGFP-ki mice were injected (i.s.) with Alexa-555-PECAM-1 to stain EC junctions of the cremaster microvasculature. After cremaster exteriorization, postcapillary venules were selected for in vivo analysis of leukocyte-vessel wall interactions.

Analysis of Lung Neutrophil Infiltration

Following perfusion of pulmonary vasculature, lungs were excised and digested. The resulting cells were stained and the number of neutrophils analyzed by flow cytometry.

Flow Cytometry

Samples were immunostained with fluorescently conjugated antibodies. Following red blood cell lysis, samples were run on a flow cytometer and analyzed using Flowjo software (TreeStar).

Measurement of Tissue Plasma Extravasation

Evans blue solution was injected i.v. and allowed to circulate for 10 min before mice were killed. Following vascular wash-out, tissues were collected and the accumulated Evans blue (tissue albumin) was quantified as a measure of plasma leakage.

Statistics

Data analysis was performed using the statistical software GraphPad Prism 4. Results are expressed as mean ± SEM. Statistical significances were assessed by Student's t test, chi-square test, one-way ANOVA with Student-Newman-Keuls multiple comparison test, or two-way ANOVA with Bonferroni post hoc test as appropriate. Correlations were quantified by Pearson's correlation coefficient. $p < 0.05$ were considered significant.

SUPPLEMENTAL INFORMATION

Supplemental Information includes four figures, Supplemental Experimental Procedures, and two movies and can be found with this article online at http://dx.doi.org/10.1016/j.immuni.2015.05.010.

AUTHOR CONTRIBUTIONS

B.C. designed and performed most experiments, analyzed and interpreted data, and contributed to the writing of the manuscript; J.V.B. performed and analyzed IVM experiments; M.B. performed and analyzed in vitro neutrophil adhesion assays; A.W. provided technical assistance for the IVM experiments; C.O. performed the JAM-C ELISA assays; C.R. and K.B. provided clinical samples and patient SOFA scores; T.C. and B.A.I. provided reagents and contributed intellectually; and S.N. provided overall project supervision, contributed to the design of the experiments, and wrote the manuscript.

ACKNOWLEDGMENTS

This work was supported by generous funds from the Wellcome Trust (098291/Z/12/Z to S.N.). T.C. was supported by the ERC (ENDHORET). The work was also supported in part by funds from the William Harvey Research Foundation.

Received: May 28, 2014
Revised: April 1, 2015
Accepted: May 11, 2015
Published: June 2, 2015

REFERENCES

Afonso, P.V., Janka-Junttila, M., Lee, Y.J., McCann, C.P., Oliver, C.M., Aamer, K.A., Losert, W., Cicerone, M.T., and Parent, C.A. (2012). LTB4 is a signal-relay molecule during neutrophil chemotaxis. Dev. Cell *22*, 1079–1091.

Aikawa, N., Ishizaka, A., Hirasawa, H., Shimazaki, S., Yamamoto, Y., Sugimoto, H., Shinozaki, M., Taenaka, N., Endo, S., Ikeda, T., and Kawasaki, Y. (2011). Reevaluation of the efficacy and safety of the neutrophil elastase inhibitor, Sivelestat, for the treatment of acute lung injury associated with systemic inflammatory response syndrome; a phase IV study. Pulm. Pharmacol. Ther. *24*, 549–554.

Auner, B., Geiger, E.V., Henrich, D., Lehnert, M., Marzi, I., and Relja, B. (2012). Circulating leukotriene B4 identifies respiratory complications after trauma. Mediators Inflamm. *2012*, 536156.

Belaaouaj, A., McCarthy, R., Baumann, M., Gao, Z., Ley, T.J., Abraham, S.N., and Shapiro, S.D. (1998). Mice lacking neutrophil elastase reveal impaired host defense against gram negative bacterial sepsis. Nat. Med. *4*, 615–618.

Beyrau, M., Bodkin, J.V., and Nourshargh, S. (2012). Neutrophil heterogeneity in health and disease: a revitalized avenue in inflammation and immunity. Open Biol. *2*, 120134.

Bradfield, P.F., Scheiermann, C., Nourshargh, S., Ody, C., Luscinskas, F.W., Rainger, G.E., Nash, G.B., Miljkovic-Licina, M., Aurrand-Lions, M., and Imhof, B.A. (2007). JAM-C regulates unidirectional monocyte transendothelial migration in inflammation. Blood *110*, 2545–2555.

Buckley, C.D., Ross, E.A., McGettrick, H.M., Osborne, C.E., Haworth, O., Schmutz, C., Stone, P.C., Salmon, M., Matharu, N.M., Vohra, R.K., et al. (2006). Identification of a phenotypically and functionally distinct population of long-lived neutrophils in a model of reverse endothelial migration. J. Leukoc. Biol. *79*, 303–311.

Cai, T.Q., and Wright, S.D. (1996). Human leukocyte elastase is an endogenous ligand for the integrin CR3 (CD11b/CD18, Mac-1, alpha M beta 2) and modulates polymorphonuclear leukocyte adhesion. J. Exp. Med. *184*, 1213–1223.

Campbell, E.J., and Owen, C.A. (2007). The sulfate groups of chondroitin sulfate- and heparan sulfate-containing proteoglycans in neutrophil plasma

membranes are novel binding sites for human leukocyte elastase and cathepsin G. J. Biol. Chem. *282*, 14645–14654.

Chen, M., Lam, B.K., Kanaoka, Y., Nigrovic, P.A., Audoly, L.P., Austen, K.F., and Lee, D.M. (2006). Neutrophil-derived leukotriene B4 is required for inflammatory arthritis. J. Exp. Med. *203*, 837–842.

Chou, R.C., Kim, N.D., Sadik, C.D., Seung, E., Lan, Y., Byrne, M.H., Haribabu, B., Iwakura, Y., and Luster, A.D. (2010). Lipid-cytokine-chemokine cascade drives neutrophil recruitment in a murine model of inflammatory arthritis. Immunity *33*, 266–278.

Donnelly, S.C., MacGregor, I., Zamani, A., Gordon, M.W., Robertson, C.E., Steedman, D.J., Little, K., and Haslett, C. (1995). Plasma elastase levels and the development of the adult respiratory distress syndrome. Am. J. Respir. Crit. Care Med. *151*, 1428–1433.

Finsterbusch, M., Voisin, M.B., Beyrau, M., Williams, T.J., and Nourshargh, S. (2014). Neutrophils recruited by chemoattractants in vivo induce microvascular plasma protein leakage through secretion of TNF. J. Exp. Med. *211*, 1307–1314.

Fujii, M., Miyagi, Y., Bessho, R., Nitta, T., Ochi, M., and Shimizu, K. (2010). Effect of a neutrophil elastase inhibitor on acute lung injury after cardiopulmonary bypass. Interact. Cardiovasc. Thorac. Surg. *10*, 859–862.

Iadecola, C., and Anrather, J. (2011). The immunology of stroke: from mechanisms to translation. Nat. Med. *17*, 796–808.

Kim, N.D., Chou, R.C., Seung, E., Tager, A.M., and Luster, A.D. (2006). A unique requirement for the leukotriene B4 receptor BLT1 for neutrophil recruitment in inflammatory arthritis. J. Exp. Med. *203*, 829–835.

Kolaczkowska, E., and Kubes, P. (2013). Neutrophil recruitment and function in health and inflammation. Nat. Rev. Immunol. *13*, 159–175.

Lämmermann, T., Afonso, P.V., Angermann, B.R., Wang, J.M., Kastenmüller, W., Parent, C.A., and Germain, R.N. (2013). Neutrophil swarms require LTB4 and integrins at sites of cell death in vivo. Nature *498*, 371–375.

Ley, K., Laudanna, C., Cybulsky, M.I., and Nourshargh, S. (2007). Getting to the site of inflammation: the leukocyte adhesion cascade updated. Nat. Rev. Immunol. *7*, 678–689.

Manetti, M., Guiducci, S., Romano, E., Rosa, I., Ceccarelli, C., Mello, T., Milia, A.F., Conforti, M.L., Ibba-Manneschi, L., and Matucci-Cerinic, M. (2013). Differential expression of junctional adhesion molecules in different stages of systemic sclerosis. Arthritis Rheum. *65*, 247–257.

Mantovani, A., Cassatella, M.A., Costantini, C., and Jaillon, S. (2011). Neutrophils in the activation and regulation of innate and adaptive immunity. Nat. Rev. Immunol. *11*, 519–531.

Mathias, J.R., Perrin, B.J., Liu, T.X., Kanki, J., Look, A.T., and Huttenlocher, A. (2006). Resolution of inflammation by retrograde chemotaxis of neutrophils in transgenic zebrafish. J. Leukoc. Biol. *80*, 1281–1288.

Matthay, M.A., and Zemans, R.L. (2011). The acute respiratory distress syndrome: pathogenesis and treatment. Annu. Rev. Pathol. *6*, 147–163.

Mayadas, T.N., Cullere, X., and Lowell, C.A. (2014). The multifaceted functions of neutrophils. Annu. Rev. Pathol. *9*, 181–218.

Mócsai, A. (2013). Diverse novel functions of neutrophils in immunity, inflammation, and beyond. J. Exp. Med. *210*, 1283–1299.

Nathan, C. (2006). Neutrophils and immunity: challenges and opportunities. Nat. Rev. Immunol. *6*, 173–182.

Nourshargh, S., and Alon, R. (2014). Leukocyte migration into inflamed tissues. Immunity *41*, 694–707.

Nourshargh, S., Hordijk, P.L., and Sixt, M. (2010). Breaching multiple barriers: leukocyte motility through venular walls and the interstitium. Nat. Rev. Mol. Cell Biol. *11*, 366–378.

Owen, C.A., and Campbell, E.J. (1999). The cell biology of leukocyte-mediated proteolysis. J. Leukoc. Biol. *65*, 137–150.

Pham, C.T. (2006). Neutrophil serine proteases: specific regulators of inflammation. Nat. Rev. Immunol. *6*, 541–550.

Phillipson, M., and Kubes, P. (2011). The neutrophil in vascular inflammation. Nat. Med. *17*, 1381–1390.

Proebstl, D., Voisin, M.B., Woodfin, A., Whiteford, J., D'Acquisto, F., Jones, G.E., Rowe, D., and Nourshargh, S. (2012). Pericytes support neutrophil subendothelial cell crawling and breaching of venular walls in vivo. J. Exp. Med. *209*, 1219–1234.

Rabquer, B.J., Amin, M.A., Teegala, N., Shaheen, M.K., Tsou, P.S., Ruth, J.H., Lesch, C.A., Imhof, B.A., and Koch, A.E. (2010). Junctional adhesion molecule-C is a soluble mediator of angiogenesis. J. Immunol. *185*, 1777–1785.

Rainger, G.E., Rowley, A.F., and Nash, G.B. (1998). Adhesion-dependent release of elastase from human neutrophils in a novel, flow-based model: specificity of different chemotactic agents. Blood *92*, 4819–4827.

Rubenfeld, G.D., Caldwell, E., Peabody, E., Weaver, J., Martin, D.P., Neff, M., Stern, E.J., and Hudson, L.D. (2005). Incidence and outcomes of acute lung injury. N. Engl. J. Med. *353*, 1685–1693.

Santoso, S., Sachs, U.J., Kroll, H., Linder, M., Ruf, A., Preissner, K.T., and Chavakis, T. (2002). The junctional adhesion molecule 3 (JAM-3) on human platelets is a counterreceptor for the leukocyte integrin Mac-1. J. Exp. Med. *196*, 679–691.

Scheiermann, C., Colom, B., Meda, P., Patel, N.S., Voisin, M.B., Marrelli, A., Woodfin, A., Pitzalis, C., Thiemermann, C., Aurrand-Lions, M., et al. (2009). Junctional adhesion molecule-C mediates leukocyte infiltration in response to ischemia reperfusion injury. Arterioscler. Thromb. Vasc. Biol. *29*, 1509–1515.

Schulte, D., Küppers, V., Dartsch, N., Broermann, A., Li, H., Zarbock, A., Kamenyeva, O., Kiefer, F., Khandoga, A., Massberg, S., and Vestweber, D. (2011). Stabilizing the VE-cadherin-catenin complex blocks leukocyte extravasation and vascular permeability. EMBO J. *30*, 4157–4170.

Smith, P.D., Puskas, F., Meng, X., Lee, J.H., Cleveland, J.C., Jr., Weyant, M.J., Fullerton, D.A., and Reece, T.B. (2012). The evolution of chemokine release supports a bimodal mechanism of spinal cord ischemia and reperfusion injury. Circulation *126* (1), S110–S117.

Souza, D.G., Coutinho, S.F., Silveira, M.R., Cara, D.C., and Teixeira, M.M. (2000). Effects of a BLT receptor antagonist on local and remote reperfusion injuries after transient ischemia of the superior mesenteric artery in rats. Eur. J. Pharmacol. *403*, 121–128.

Tak, T., Tesselaar, K., Pillay, J., Borghans, J.A., and Koenderman, L. (2013). What's your age again? Determination of human neutrophil half-lives revisited. J. Leukoc. Biol. *94*, 595–601.

Vestweber, D., Wessel, F., and Nottebaum, A.F. (2014). Similarities and differences in the regulation of leukocyte extravasation and vascular permeability. Semin. Immunopathol. *36*, 177–192.

Voisin, M.B., and Nourshargh, S. (2013). Neutrophil transmigration: emergence of an adhesive cascade within venular walls. J. Innate Immun. *5*, 336–347.

Weninger, W., Biro, M., and Jain, R. (2014). Leukocyte migration in the interstitial space of non-lymphoid organs. Nat. Rev. Immunol. *14*, 232–246.

Wheeler, A.P., and Bernard, G.R. (2007). Acute lung injury and the acute respiratory distress syndrome: a clinical review. Lancet *369*, 1553–1564.

Woodfin, A., Voisin, M.B., Beyrau, M., Colom, B., Caille, D., Diapouli, F.M., Nash, G.B., Chavakis, T., Albelda, S.M., Rainger, G.E., et al. (2011). The junctional adhesion molecule JAM-C regulates polarized transendothelial migration of neutrophils in vivo. Nat. Immunol. *12*, 761–769.

Yoo, S.K., and Huttenlocher, A. (2011). Spatiotemporal photolabeling of neutrophil trafficking during inflammation in live zebrafish. J. Leukoc. Biol. *89*, 661–667.

Young, R.E., Voisin, M.B., Wang, S., Dangerfield, J., and Nourshargh, S. (2007). Role of neutrophil elastase in LTB4-induced neutrophil transmigration in vivo assessed with a specific inhibitor and neutrophil elastase deficient mice. Br. J. Pharmacol. *151*, 628–637.

Immunity

Supplemental Information

Leukotriene B_4-Neutrophil Elastase Axis Drives Neutrophil Reverse Transendothelial Cell Migration In Vivo

Bartomeu Colom, Jennifer V. Bodkin, Martina Beyrau, Abigail Woodfin, Christiane Ody, Claire Rourke, Triantafyllos Chavakis, Karim Brohi, Beat A. Imhof, and Sussan Nourshargh

SUPPLEMENTAL INFORMATION

SUPPLEMENTAL FIGURES

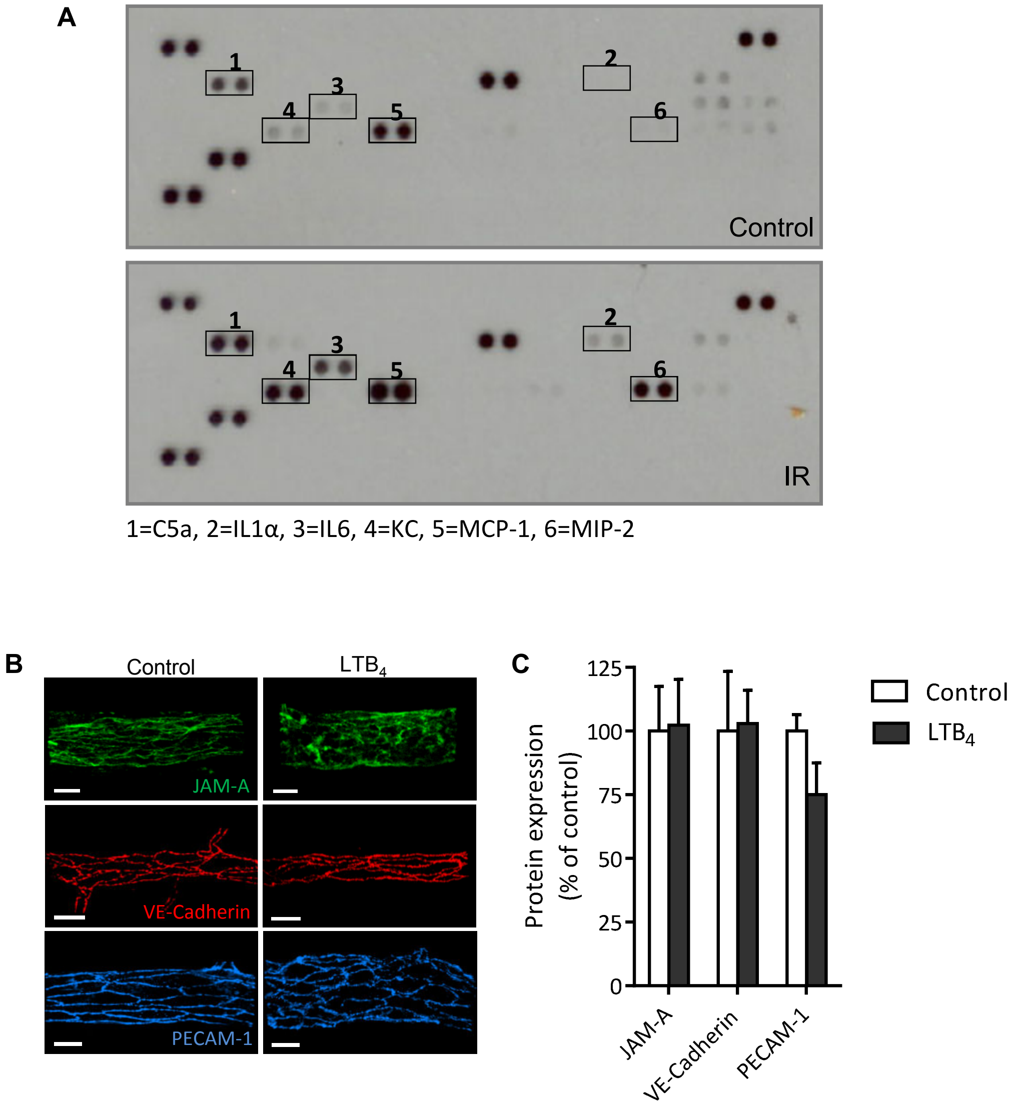

Figure S1

Figure S1. Related to Figure 1. Characterization of the inflammatory mediator generation and expression profile of EC adhesion molecules in response to cremaster muscle ischemia-reperfusion injury or intradermal injection of LTB_4, respectively. (A) The inflammatory mediator expression profile in sham (control) and I-R stimulated cremaster muscles was measured in tissue homogenates (pooled samples from 3 mice per group) using a Mouse Cytokine Array Panel A kit. Densitometry from the blots was analyzed with ImageJ software. Images are representative of 2 independent experiments. (B-C) Locally administered LTB_4 does not impact the expression profile of EC JAM-A, VE-cadherin or PECAM-1. Images (B) and quantification of protein expression levels (C) of the indicated adhesion molecules at EC junctions of mouse ear dermal post-capillary venules in control (B, left panels) and in stimulated tissues (4h i.d. LTB_4) (B, right panels), as analyzed by immunofluorescent staining and confocal microscopy (n=4 mice) from 3 independent experiments. Data are percentage change in mean fluorescent intensity (MFI) of signals acquired from stimulated samples relative to controls and presented as mean ± SEM. Scale bars, 20μm.

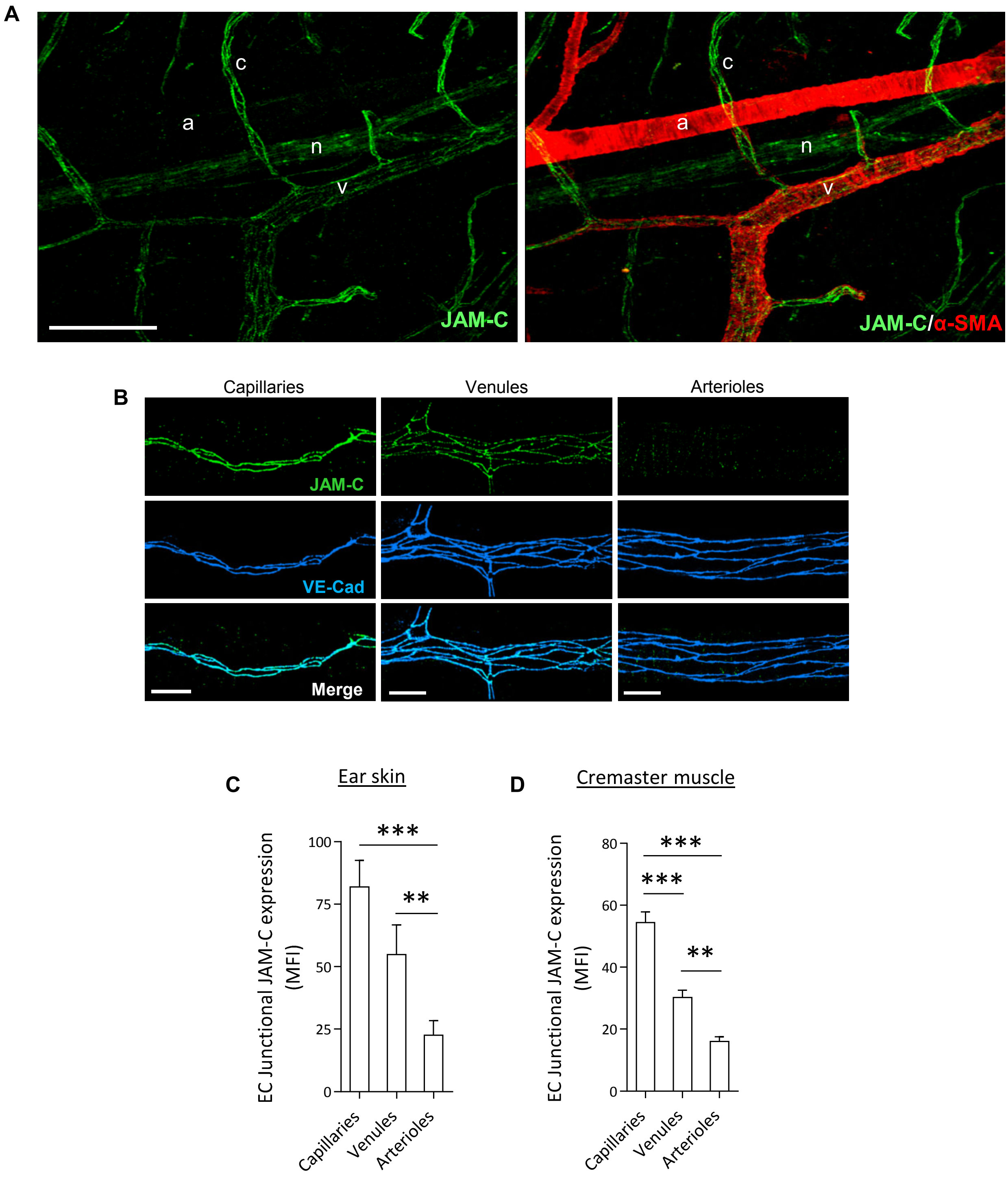
A
c
a
n
v
JAM-C
JAM-C/α-SMA
B
Capillaries
Venules
Arterioles
JAM-C
VE-Cad
Merge
C
Ear skin
EC Junctional JAM-C expression (MFI)
100
75
50
25
0
***
**
Capillaries
Venules
Arterioles
D
Cremaster muscle
EC Junctional JAM-C expression (MFI)
80
60
40
20
0
***
***
**
Capillaries
Venules
Arterioles

Figure S2

Figure S2. Related to Figure 2. JAM-C is expressed at different levels in different types of microvessels. (A) Mouse ears were immunostained for JAM-C and α-SMA. The images show JAM-C expression in all microvessels but with differing levels: JAM-C expression was greatest in capillaries (c, α-SMA negative), followed by venules (v) and was low in arterioles (a). JAM-C was also noted in nerves (n). (B) High magnification images of mouse ear dermal blood vessels illustrating localization of JAM-C to EC junctions (as shown by co-localisation with VE-cadherin) and again indicating different expression levels of JAM-C in capillaries, venules and arterioles. (C-D) Quantification of JAM-C protein levels at junctions of ECs in different blood vessel types in ear skin (C) and cremasters (D), as analyzed by confocal microscopy (n=3-7) from 5 independent experiments. Data indicate mean fluorescent intensity (MFI) ± SEM. ** $P<0.01$ and *** $P<0.001$ as indicated by lines. Scale bars, 100µm (A), 20µm (B).

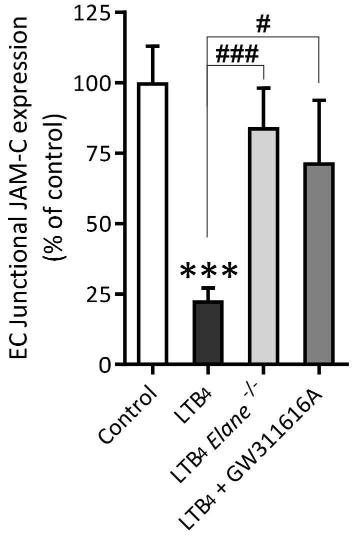

Figure S3. Related to Figure 3. Neutrophil elastase mediates LTB_4-induced cleavage of EC JAM-C. LTB_4-stimulated cremaster muscles of WT, *Elane*$^{-/-}$ or WT mice treated with the NE inhibitor GW311616A were analysed for junctional expression of EC JAM-C and compared to control unstimulated tissues (n=3-4) involving 4 independent experiments. Data indicate mean ± SEM. ***P<0.001 as compared to controls and $^{\#}$P<0.05 and $^{\#\#\#}$P<0.001 as indicated by lines.

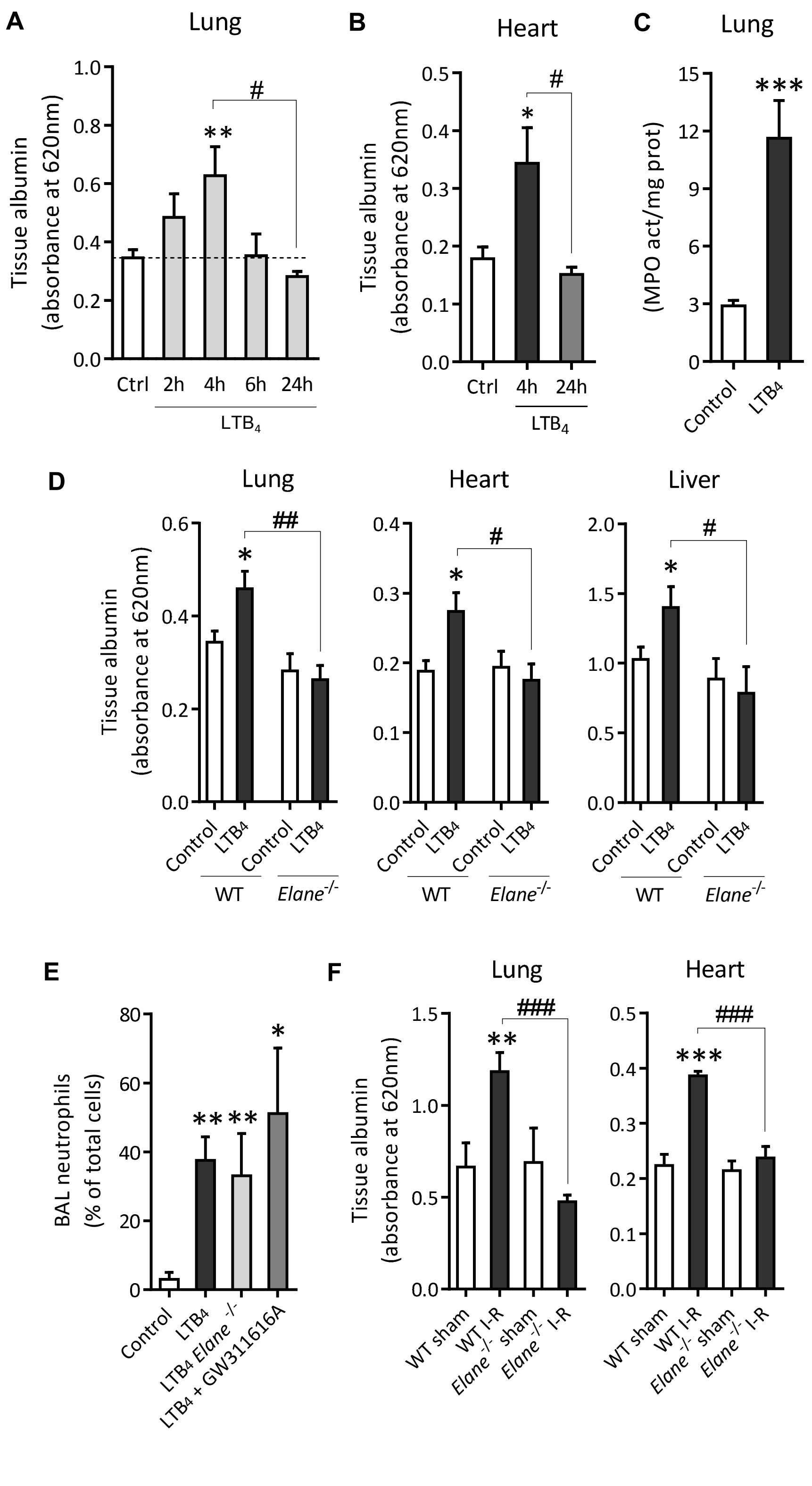
A
Lung
Tissue albumin (absorbance at 620nm)
Ctrl
2h
4h
6h
24h
LTB4
**
#
B
Heart
Tissue albumin (absorbance at 620nm)
Ctrl
4h
24h
LTB4
*
#
C
Lung
(MPO act/mg prot)
Control
LTB4
***
D
Lung
Heart
Liver
Tissue albumin (absorbance at 620nm)
Control
LTB4
WT
Elane-/-
##
#
*
E
BAL neutrophils (% of total cells)
Control
LTB4
LTB4 Elane -/-
LTB4 + GW311616A
F
Lung
Heart
Tissue albumin (absorbance at 620nm)
WT sham
WT I-R
Elane-/- sham
Elane-/- I-R
###
**
***

Figure S4

Figure S4. Related to Figure 6. The LTB_4-NE axis promotes distant organ damage. (A-B) Time course of remote organ damage following LTB_4 stimulation of cremaster muscle. Quantification of lung (A) and heart (B) albumin content (plasma extravasation), as an indicator of tissue damage, following local (cremaster, intrascrotal) injection of LTB_4 at the indicated time points as compared to control (Ctrl) unstimulated tissues (n=3-20 from 15 independent experiments). (C) Intradermal administration of LTB_4 into the mouse ear skin promotes lung inflammation. LTB_4 was injected into mouse ears intradermally and 4h later, the lungs were excised and analysed for neutrophil infiltration as quantified by measurement of tissue MPO enzymatic activity. Unstimulated ears acted as controls (n=4-6 from 2 independent experiments). (D) Intradermal injection of LTB_4 in the mouse ear promotes multi-organ distant damage in an NE-dependent manner. Quantification of remote tissue albumin content following local (ear, intradermal) injection of LTB_4 (4h) as compared to control unstimulated tissues in WT and *Elane*$^{-/-}$ mice (n=4-24 from 11 independent experiments). (E) LTB_4-induced neutrophil recruitment to lungs is NE-independent. WT, *Elane*$^{-/-}$ mice or WT mice pretreated (orally, 24h) with the NE inhibitor GW311616A were stimulated with intranasal LTB_4 and 24h later neutrophil infiltration into the airways was quantified in bronchoalveolar lavage (BAL). Control mice received LTB_4 vehicle (n=3-6 from 3 independent experiments). (F) Cremaster I-R injury induces multi-organ distant damage. Quantification of remote tissue albumin content in WT and *Elane*$^{-/-}$ mice following I-R injury of the cremaster muscle as compared to sham operated mice (n=3-7 from 5 independent experiments). Data indicate mean ± SEM. *P<0.05, **P<0.01 and ***P<0.001 as compared to controls and $^{\#}$P<0.05, $^{\#\#}$P<0.01 and $^{\#\#\#}$P<0.001 as indicated by lines.

SUPPLEMENTAL MOVIE LEGENDS

Movie S1. Related to Figure 1. Neutrophil reverse TEM as induced by I-R injury. The movie captures an inflammatory response in a cremasteric venule of a *Lyz2-EGFP-ki* mouse (exhibiting GFP myeloid cells), immunostained *in vivo* for EC junctions with Alexa Fluor-555-labeled anti-PECAM-1 mAb 390 (red) and stimulated with I-R. The clip shows high optical zoom of a neutrophil migrating through a multi-cellular junction viewed from the luminal side. The neutrophil (green) is initially on the abluminal (sub-EC) side of the endothelial junction and subsequently migrates through the junction in an abluminal to luminal or 'reverse' direction. Breaching the EC barrier results in the transient formation of an exit pore, as indicated in the movie. On the luminal side the leukocyte disengages from the junction and crawls across the luminal surface. Still images of this sequence are shown in Fig. 1D. Of note, the movie has been created using a software (IMARIS™, Bitplane) that recreates the structures being imaged from individual voxels in 3D via a blend projection algorithm whilst maintaining a transparency function. As a result, when observing neutrophil migration from the luminal side, neutrophils in the vascular lumen are seen as being fully green (closest to the viewing direction) whilst cells in the sub-EC space and/or within EC junctions are visualized as green cells with an overlay of red fluorescence (further away from the viewing direction). Similarly when viewing events from the abluminal side, the sub-EC neutrophil is seen as being fully green due to it being close to the viewing direction.

Movie S2. Related to Figure 1. Neutrophil reverse TEM as induced by topical LTB_4. The movie captures an inflammatory response in a cremasteric venule of a *Lyz2-EGFP-ki* mouse (exhibiting GFP myeloid cells, green), immunostained *in vivo* for EC junctions with Alexa Fluor-555-labeled anti-PECAM-1 mAb 390 (red), as induced by locally administered LTB_4. The clip shows high optical zoom of a neutrophil migrating through a multi-cellular junction viewed from the luminal side. The neutrophil (green) is initially on the luminal side of the endothelial cell, transmigrates into the sub-endothelial cell space and subsequently migrates through the junction back into the lumen in a 'reverse' direction. On the luminal side the leukocyte disengages from the junction and crawls across the luminal surface. The movie has been created using a software (IMARIS™, Bitplane) that recreates the structures being imaged from individual voxels in 3D via a blend projection algorithm whilst maintaining a transparency function. As a result, since the neutrophil rTEM event is being observed from the luminal side, neutrophils in the vascular lumen are seen as being fully green (closest to the

viewing direction) whilst cells in the sub-EC space and/or within EC junctions are visualized as green cells with an overlay of red fluorescence (further away from the viewing direction).

SUPPLEMENTAL EXPERIMENTAL PROCEDURES

Reagents/antibodies. Recombinant murine C5a, CXCL2 (MIP-2) and IL1β were purchased from R&D Systems (Abingdon, Oxford, UK). CXCL1 (KC) was from AbD Serotec (Oxford, UK). LTB_4 and PVDF membranes were purchased from Calbiochem (Merck Millipore, Nottingham UK). Tyrode's salt, FCS, PFA, EDTA, Triton X-100, HEPES solution, BSA, GW311616A, Ripa buffer, collagenase, Human MPO, purified Human IgG, glutaraldehyde, DNAse, anti-mouse α-SMA antibody (clone 1A4), lipopolysaccharide (LPS) and Evans blue were from Sigma-Aldrich (Poole, Dorset, UK). LY293111 was obtained from Cambridge Bioscience (Cambridge, UK). Recombinant murine JAM-C was generated as described before (Aurrand-Lions et al., 2001). JAM-B-Fc, Fc control protein, Recombinant human ICAM-1, Mouse Cytokine Array Panel A Array Kit and KC, IL1β and LTB_4 ELISA kits were from R&D systems (Abingdon, Oxford, UK). NE680FAST was obtained from Perkin Elmer (Buckinghamshire, UK). NGS was from PAA Laboratories (Somerset, UK). Purified human NE was purchased from Enzo Life Sciences (Exeter, UK). Halt Protease Phosphatase Inhibitor Cocktail, Supersignal West Pico Chemoluminescent Substrate and 16-well glass chamber slides (NUNC) were from Thermo Scientific (Cramlington, UK). Formamide and Sure Blue kit reagent were from VWR (Leicestershire, UK). Enhanced K-Blue TMB Substrate was from Neogen Corporation (Lexington, KY, USA). Anti-Ly-6G MicroBead Kit was from Miltenyi Biotec (Surrey, UK). RPMI 1640 medium was from Gibco (UK). Alexa-Fluor monoclonal antibody labelling kits, 2-mercaptoethanol, Dynabeads sheep anti-rat IgG and Alexa fluorescently-labelled secondary antibodies were from Invitrogen (Paisley UK). Anti-mouse antibodies against PECAM-1 (clone 390), VE-cadherin (clone BV14), CD11b (Mac-1, clone M1/70) and CD115 (clone AFS98), and the isotype controls IgG2b and IgG2a were purchased from eBiosciences (Hatfield, UK). Anti-JAM-A (clone H2O2-106-7-4) was a gift from Dr Michel Aurrand-Lions (INSERM, Centre de Recherche en Cancerologie de Marseille, France) and was generated as previously detailed (Malergue et al., 1998). Ultra-LEAF™ Purified anti-mouse Ly-6G (clone 1A8) was from BD Biosciences (Cowley, Oxford, UK). Anti-MRP14 (Hobbs et al., 2003) (clone 2B10) was a gift from Dr N. Hogg (Cancer Research UK, London,

UK). Rabbit polyclonal antibodies against CD11b (Mac-1) and NE were from Abcam (Cambridge, UK). Antibodies against Ly6G (clone 1A8) and CD45 (clone 30-F11) were obtained from Biolegend (London, UK). Rabbit Polyclonal anti-JAM-C was generated as previously described (Lamagna et al., 2005).

Animals. *Lyz2-EGFP-ki* mice (Faust et al., 2000) were used with the permission of Dr Thomas Graf (Center for Genomic Regulation and ICREA, Barcelona, Spain.) and were kindly provided by Dr Markus Sperandio (Ludwig-Maximilians University, Munich, Germany). In these animals the gene for *EGFP* has been knocked into the lysozyme M (*lyz2*) locus, yielding mice that exhibit fluorescent myelomonocytic cells, with mature neutrophils comprising the highest percentage of $EGFP^{hi}$ cells. Mice deficient in neutrophil elastase (*$Elane^{-/-}$*) (Belaaouaj et al., 1998) were a gift from Professor S Shapiro (Harvard Medical School, Boston, MA, USA). *$Elane^{-/-}$* mice were crossed with *Lyz2-EGFP-ki* mice to generate a new colony (*$Elane^{-/-}$;Lyz2-EGFP-ki*) exhibiting NE deletion and GFP-tagged neutrophils. Endothelial cell specific JAM-C deficient mice (*$Tekcre;JAM\text{-}3^{flox/flox}$*) were generated in house as described before (Woodfin et al., 2011) by cre-mediated recombination of *JAM-3* flanked by *loxP* sites (Langer et al., 2011) under the control of the *Tek*-promoter. Wild type C57BL/6 mice were obtained from Harlan-Olac (Bicester, UK). All animal experiments were conducted in accordance with the United Kingdom Home Office legislations.

Patients. The study was approved by the East London and City Research Ethics Committee. All adult trauma patients (>15 years) who met the local criteria for trauma team activation were eligible for enrolment into the Activation of Coagulation and Inflammation in Trauma (ACIT) 2 study. ACIT2 is a study prospectively evaluating aspects of coagulation and inflammation in trauma patients. Exclusion criteria were; arrival at hospital more than 2-hours after injury, transfer from another hospital, known severe liver disease, known bleeding diathesis, administration of >2000ml of fluid prior to enrolment or a burn injury covering more than 5% of the total body surface area. Acute respiratory distress syndrome (ARDS) was defined using the Berlin consensus definitions (Force et al., 2012). Organ failure at 48h was described using the SOFA Score (Vincent et al., 1998).

Induction of inflammatory reactions and pre-treatments. Mice were anesthetized by intramuscular (i.m.) injection of 1ml/kg of anesthetic mix (40mg ketamine and 2mg xylazine in saline) before the inflammatory stimuli, namely LTB_4 (300ng), LPS (300ng), CXCL1 (KC)

(500ng), CXCL2 (MIP-2) (500ng), C5a (1µg) or vehicle control, were injected in the ears (30µl, intradermally, 4h) or the cremaster muscles (400µl, intrascrotally, 4h). In some experiments, purified human NE (1mg/kg) with or without KC (500ng) was injected locally for 4h into cremaster muscles and the NE inhibitor GW311616A (2mg/kg) (Macdonald et al., 2001) was orally administrated 24h before induction of inflammation. Cremaster I-R injury was induced in anesthetized mice as previously detailed (Scheiermann et al., 2009). Briefly, the blood flow to the muscle was stopped by placing a clamp at the base of the exteriorized tissue for 30 min to induce ischemia, after which the clamp was removed to allow reperfusion over a 2h period. Control sham operated mice underwent surgical procedures but not tissue I-R. In some animals the LTB_4 receptor antagonist LY293111 (10mg/Kg) was administered i.v. 15 minutes prior to induction of I-R.

Whole-mount tissue immunofluorescence staining. Ears or cremaster muscles were dissected and fixed for 10min in ice-cold PFA 4%. Samples were blocked/permeabilized for 2h at room temperature in PBS containing 12.5% FCS, 12.5% NGS and 0.5% Triton X-100, followed by overnight incubation at 4°C with primary antibodies in PBS containing 5% NGS and 5% FCS. For double or triple staining, some antibodies were directly conjugated with Alexa dyes using commercial Alexa-Fluor monoclonal antibody labelling kits. Otherwise, tissues were incubated with appropriate Alexa fluorescently-labelled secondary antibodies for 3h at 4°C in PBS containing 5% NGS and 5% FCS. After washings, tissues were mounted on slides and analyzed by confocal microscopy.

Confocal microscopy. Immunofluorescently stained whole-mount tissues were imaged using a Zeiss LSM 5 PASCAL confocal laser-scanning microscope (Carl Zeiss) equipped with Argon (excitation wavelength: 488nm) and HeNe (excitation wavelengths: 543 and 633nm) lasers, or a Leica SP5 (Leica) equipped with argon and helium-neon lasers. Multiple Z-stack images at a resolution of 1024 x 1024 were acquired with an oil immersion Plan-Apochromat 63x (1.4 NA) objective or a 20× water-dipping objective (1.0 NA). The protein expression of junctional EC adhesion molecules was quantified in 3D reconstructed images using IMARIS software (Bitplane) as described before (Colom et al., 2012; Woodfin et al., 2011). Briefly, an isosurface was created using the VE-Cadherin or PECAM-1 labelled channels and the intensity of immunoreactive proteins of interest within these channels was quantified. Total expression of JAM-C was analyzed using Image J software. The samples were also analyzed for

quantification of number of transmigrated neutrophils per field of view using the 3D images and IMARIS software.

Quantification of plasma soluble JAM-C content. Mouse blood was obtained by cardiac puncture and collected in heparin. Samples were centrifuged at 6000xg for 3 min and plasma was collected, frozen in liquid N_2 and kept at -80C°. Human plasma was prepared post double centrifugation of blood, collected in buffered sodium citrate, at 1760xg for 10 min and supernatants were then frozen and stored at -80C°. sJAM-C content in plasma was measured by ELISA as follows. ELISA plates were coated with mouse or human JAM-B-Fc or Fc control protein in bicarbonate buffer (100mM pH 9.6). Wells were blocked with a solution of PBS containing 0.05% Tween-20 (PBS-T), 3% BSA, 0.2% gelatin (for human only) and 10µg/ml of purified IgG. After washing in PBS-T, plasma samples diluted 1:1 in PBS-T were added to the wells and incubated overnight at 4°C. Wells were then washed twice in PBS-T, once in PB and then incubated with 10µg/ml monoclonal anti-mouse JAM-C (H36) or 5µg/ml affinity purified rabbit polyclonal anti-human JAM-C (714) (Lamagna et al., 2005; Ody et al., 2007). Finally, samples were incubated with a goat anti-rat or anti-rabbit antibody conjugated to HRP and the peroxidase activity was measured with Sure Blue kit reagent using a LEDETECT96 plate reader. Calculations were performed on the linear part of the calibration curve. Soluble mouse JAM-C of non-stimulated animals was at the limit of the detection level.

Cytokine/chemokine expression profile. Mouse cremasters were homogenized in 500µl PBS containing 1% Triton and 1% Halt Protease and Phosphatase Inhibitor Cocktail using the Precellys24 beat-beading system (Bertin Technologies, France). Samples were quickly frozen in liquid N_2, thawed, centrifuged 5 min at 10000xg and the supernatant collected for subsequent analysis. The cytokine/chemokine expression profile of the samples (pooled samples from 3 mice/group) was analyzed using a Mouse Cytokine Array Panel A Array Kit as per manufacturer instructions. Densitometry from the blots was analyzed with ImageJ software. The expression of selected inflammatory mediators (KC, IL1β and LTB_4) was analyzed by ELISA using commercial kits.

***In vitro* digestion of JAM-C.** Purified recombinant murine JAM-C (Aurrand-Lions et al., 2001) (5µg) was incubated for 1h at 37°C in digestion buffer consisting of 0.2M Tris, 0.15M NaCl and 0.02M $CaCl_2$ at pH 7.4, in the absence or presence of 0.05, 0.2 or 1µg of purified

NE, within a total final reaction volume of 15µl. Samples were then resolved in a SDS-PAGE gel and visualized by Western blot.

Co-immunoprecipitation. Bone marrow (BM) derived neutrophils were purified using an anti-Ly-6G MicroBead Kit as per the manufacturer's instructions. Neutrophils ($3x10^5$) were left unstimulated or stimulated with LTB_4 (100nM) for 30min at room temperature in RPMI medium containing HEPES (25mM), FCS (10%), GW311616A (5µM), Halt Protease-Phosphatase Inhibitor Cocktail (1%), purified NE (5µg) and JAM-C (5µg) (Aurrand-Lions et al., 2001). Samples were then lysed in RIPA buffer and pre-cleared with Dynabeads (20µl) sheep anti-rat IgG at 4°C for 1h. Dynabeads were removed by centrifugation (1min, 2000xg) and the supernatant was incubated overnight at 4°C with an anti-Mac-1 mAb (5µg). Samples were then incubated with Dynabeads for 3h at 4°C. Following centrifugation, Dynabeads were resuspended in PBS and boiled for 10min in loading buffer containing 2% 2-mercaptoethanol. JAM-C and NE protein content was analyzed both in lysates and anti-Mac-1 immunoprecipitated samples by Western blot.

Immuno blot. Samples were boiled for 10min in loading buffer containing 2% 2-mercaptoethanol and resolved on SDS-PAGE gels. Proteins were electrotransferred onto PVDF membranes followed by 1h blocking in TBS-Tween containing 5% non-fat milk and incubation overnight at 4°C with primary antibodies in 5% BSA TBS-Tween buffer. After incubation with HRP-conjugated secondary antibodies, membranes were developed using the Supersignal West Pico Chemoluminescent Substrate.

***In vitro* neutrophil adhesion assay.** 16-well glass chamber slides were coated with recombinant human ICAM-1 (2.5µg/ml) in coating buffer (150 mM NaCl, 20 mM Tris-HCl, 2 mM MgCl2, pH 9.0) overnight at 4°C. Slides were then washed and blocked with 10% BSA in PBS for 1h at room temperature. BM neutrophils were isolated from WT mice using an anti-Ly-6G MicroBead Kit. Neutrophils ($5x10^4$/well) in RPMI 1640 media, 10% FCS and 25 mM HEPES were either left untreated or stimulated with LTB_4 (1-100nM) or KC (1-100nM) for 30 min at room temperature. In some experiments neutrophils were pre-incubated with an anti-Mac-1 mAb or an isotype control IgG (both at 40 µg/ml) for 20 min at room temperature. Slides were then washed twice in PBS and fixed in 3% PFA + 0.5% glutaraldehyde in PBS for 2 hours on ice. The number of adherent cells per field of view was quantified from images acquired by phase contrast microscopy.

Imaging of JAM-C cleavage *in vitro*. 16-well glass chamber slides were coated with recombinant ICAM-1 and JAM-C (2.5µg/ml) as above. BM neutrophils ($5x10^4$/well) were left untreated or treated with LTB_4 (100nM), LTB_4+ GW311616A (5µM) or KC (100nM) for 30min. Slides were then fixed as above and immunostained with antibodies against JAM-C and MRP-14. Slides were analyzed by confocal microscopy and the expression of JAM-C at sites of neutrophil adhesion was quantified with IMARIS software by creating an isosurface on the MRP-14 channel and measuring the intensity of JAM-C within this surface. In some experiments, *Elane*$^{-/-}$ BM neutrophils were pre-incubated with an anti-Mac-1 (40µg/ml) or isotype control mAb prior to being stimulated with LTB_4 (30 min) in the presence or absence of purified NE (0.1mg/ml).

Mouse neutrophil depletion protocol. Specific depletion of circulating neutrophils was achieved by a single i.p. injection of 150 µg of Ultra-LEAF™ Purified anti-mouse Ly-6G mAb for 24h. The protocol resulted in >99% depletion of circulating neutrophils as determined by flow cytometry, while the number of monocytes was not affected. Control non-depleted groups were injected with an isotype-matched mAb.

***In vivo* NE enzymatic activity assay.** The NE-fluorescent activatable substrate NE680FAST (Kossodo et al., 2011) was injected i.v. (4.8nmols) into anesthetized mice and left to circulate for 15 min before induction of ear inflammation as detailed above. Tissues were then collected, fixed in 4% PFA as described above and immunofluorescently stained for VE-Cadherin before being analyzed by confocal microscopy. NE activity was quantified by the fluorescence of the peptide NE680FAST, as measured using ImajeJ software.

***In vitro* NE enzymatic activity assay.** The NE-fluorescent activatable substrate NE680FAST was used to assay NE activity released from BM neutrophils *in vitro*. Briefly cells ($5x10^4$/well) on ICAM-1 and JAM-C coated slides were left untreated or stimulated for 30min with KC (100nM) or LTB_4 (100nM) in the presence of NE680FAST (1µM/well). Slides were then washed, fixed, immunostained for neutrophils using an anti-MRP-14 mAb, and the intensity of NE680FAST on the isosurface of MRP-14 channel was analyzed by confocal microscopy and measured with IMARIS software as detailed above.

Confocal intravital microscopy (IVM) of mouse cremaster muscles. Confocal intravital microscopy analysis of the mouse cremaster muscle was conducted as previously detailed (Woodfin et al., 2011). Briefly, *Lyz2-EGFP-ki* mice (exhibiting predominantly $EGFP^{hi}$ neutrophils) were injected (i.s.) with an Alexa 555-conjugated mAb against PECAM-1 (4µg) for 2h to stain EC junctions of the cremaster microvasculature. After cremaster exteriorization, postcapillary venules (20-40µm diameter) of stimulated tissues were selected for *in vivo* analysis of leukocyte-vessel wall interactions using a Leica SP5 confocal microscope incorporating a 20× water-dipping objective (NA 1.0). Acquisition of 3D confocal images over time yielded high-resolution four-dimensional videos of dynamic events that were analysed with IMARIS 4D modelling software (Bitplane; for more details see below). Neutrophils exhibiting reverse TEM (rTEM) were defined as cells that moved in an abluminal-to-luminal direction within endothelial cell junctions (stained with an anti-PECAM-1 mAb). This included cells that fully or partially breached the endothelium from the vascular lumen before exhibiting reverse motility through EC junctions and re-entering the blood flow. Within this overall definition, neutrophils were observed to fully breach EC junctions and completely enter the sub-endothelial cell space (where on occasion sub-EC motility was observed), before reverse migrating back through the endothelium into the vascular lumen. In some instances, neutrophils exhibited partial migration into EC junctions (~70-80% of the cell body), before reverse migrating towards the vascular lumen and re-entering the blood circulation. In all cases of neutrophil rTEM, the cells ultimately showed reverse motility within EC junctions (abluminal-to-luminal), ending up in the vascular lumen after disengagement from EC junctions. In contrast, normal neutrophil TEM was classified as a response in which the cells migrated through EC junctions only in a luminal-to-abluminal direction and with no pause (Woodfin et al., 2011).

All the movies and images are representative 4D and 3D images, respectively, acquired using a complex algorithmic software (IMARIS™, Bitplane). This software recreates the structures being imaged from individual voxels in 3D by using a blend projection algorithm that enables the mixing of voxel values along the viewing direction whilst maintaining a transparency function. In the movies, rTEM responses are largely being viewed from the luminal side of the vessel, which results in a visual overlap of voxels from the Alexa Fluor 555 (PECAM-1; closer to the viewing point) with that of GFP-neutrophils (further away from the viewing point). Hence when the neutrophil is in the sub-EC space or at EC junctions, the leukocyte is seen to be green with an overlay of red fluorescence. In contrast, when the neutrophil is in the vascular

lumen, the voxels nearest to the viewing direction stem from GFP, resulting in the neutrophils being totally green.

Analysis of lung neutrophil infiltration. Following stimulation of cremaster muscles, the left carotid artery of anesthetized mice was cannulated, a bolus injection of heparin (50U) administered and the mice fully exsanguinated. After ligation of the carotid artery, the cannula was removed and mice were killed by cervical dislocation. The chest cavity was immediately opened and both the thoracic vena cava and aorta just above the diaphragm were clamped. The pulmonary vasculature was then perfused with 10 ml of warm PBS (containing heparin and EDTA) by direct injection into the right ventricle, and collection via a cannula inserted into the left ventricle. Lungs were then excised and digested for 30min at 37°C in 5 ml PBS containing collagenase and DNAse (500 U each). The digested lungs were passed through 40 μm cell strainers and the collected cell suspension centrifuged at 400xg for 10min at 4°C. Finally, cells were stained and the number of neutrophils analyzed by flow cytometry as detailed below.

Flow cytometry. The efficiency and specificity of the leukocyte depletion protocol as well as lung neutrophil counts were assessed by flow cytometry. Samples were collected and incubated with anti-mouse CD16-CD32 to block Fc receptor-mediated antibody binding (5μg/ml), before staining with fluorescently conjugated antibodies against the pan leukocyte marker CD45, the monocyte marker CD115 and the neutrophil marker Ly6G. Red blood cells were then lysed with ACK lysis buffer (150mM NH_3Cl, 1mM $KHCO_3$ and 1mM EDTA) and immunoreactive molecules of interest were measured on a LSR Fortessa flow cytometer (BD) and analyzed using Flowjo software (TreeStar).

MPO enzymatic activity assay. Mouse ears were stimulated i.d. for 4h with LTB_4 or vehicle control. Animals were killed and lungs were excised and homogenized in 1ml of homogenizing buffer (600mM NaCl, 0.5% HTAB, 600mM KH_2PO_4 and 66mM Na_2HPO_4) using the Precellys24 beat-beading system (Bertin Technologies, France). Samples were then subjected to two cycles of liquid N_2 freezing-thawing and homogenized again followed by centrifugation at 13000xg (10min at 4°C). MPO activity of lung supernatants was measured through the use of Enhanced K-Blue TMB Substrate (Oxford Byosystems) with the increase in absorbance at 650nm being measured every 30s for 15min at 37°C using a spectrophotometer (Spectra MR, Dynex Technologies). The enzyme activity was calculated using a standard curve generated with human MPO and expressed as Units/mg of protein.

Analysis of neutrophils in the bronchoalveolar lavage (BAL). Mice were intranasally challenged with LTB_4 (2µg) or vehicle control. After 24h, animals were anesthetized, the chest cavity was opened and the trachea exposed. A catheter was then inserted into the trachea and used to flush the lungs with 2ml PBS containing 0.5mM EDTA. BAL fluid was collected and the % of neutrophils was analyzed by flow cytometry as described above.

Measurement of tissue plasma extravasation. Following local stimulation of cremaster muscles or ears (intradermal) with CXCL1 (KC) (500ng), LTB_4 (300ng) or I-R injury (cremaster) in mice with or without pre-treatment with GW311616A (2mg/kg, 24h, orally) (Macdonald et al., 2001), Evans blue solution (5µl of a 5% solution per gram of mouse weight,) was injected i.v. into the mice and allowed to circulate for 10 min before the animals were killed. Following vascular wash-out (with PBS containing 5mM EDTA), tissues were collected and the accumulated Evans blue (tissue albumin) was eluted in 1ml of formamide for 24 h at 55°C. Optical density (OD) readings at 620 nm were normalized to formamide alone and used as a measure of plasma extravasation.

SUPPLEMENTAL REFERENCES

Aurrand-Lions, M., Duncan, L., Ballestrem, C., and Imhof, B.A. (2001). JAM-2, a novel immunoglobulin superfamily molecule, expressed by endothelial and lymphatic cells. J Biol Chem *276*, 2733-2741.

Colom, B., Poitelon, Y., Huang, W., Woodfin, A., Averill, S., Del Carro, U., Zambroni, D., Brain, S.D., Perretti, M., Ahluwalia, A., *et al.* (2012). Schwann cell-specific JAM-C-deficient mice reveal novel expression and functions for JAM-C in peripheral nerves. FASEB J *26*, 1064-1076.

Faust, N., Varas, F., Kelly, L.M., Heck, S., and Graf, T. (2000). Insertion of enhanced green fluorescent protein into the lysozyme gene creates mice with green fluorescent granulocytes and macrophages. Blood *96*, 719-726.

Force, A.D.T., Ranieri, V.M., Rubenfeld, G.D., Thompson, B.T., Ferguson, N.D., Caldwell, E., Fan, E., Camporota, L., and Slutsky, A.S. (2012). Acute respiratory distress syndrome: the Berlin Definition. JAMA *307*, 2526-2533.

Hobbs, J.A., May, R., Tanousis, K., McNeill, E., Mathies, M., Gebhardt, C., Henderson, R., Robinson, M.J., and Hogg, N. (2003). Myeloid cell function in MRP-14 (S100A9) null mice. Mol Cell Biol *23*, 2564-2576.

Kossodo, S., Zhang, J., Groves, K., Cuneo, G.J., Handy, E., Morin, J., Delaney, J., Yared, W., Rajopadhye, M., and Peterson, J.D. (2011). Noninvasive in vivo quantification of neutrophil elastase activity in acute experimental mouse lung injury. Int J Mol Imaging *2011*, 581406.

Lamagna, C., Hodivala-Dilke, K.M., Imhof, B.A., and Aurrand-Lions, M. (2005). Antibody against junctional adhesion molecule-C inhibits angiogenesis and tumor growth. Cancer Res *65*, 5703-5710.

Langer, H.F., Orlova, V.V., Xie, C., Kaul, S., Schneider, D., Lonsdorf, A.S., Fahrleitner, M., Choi, E.Y., Dutoit, V., Pellegrini, M.*, et al.* (2011). A Novel Function of Junctional Adhesion Molecule-C in Mediating Melanoma Cell Metastasis. Cancer Res *71*, 4096-4105.

Macdonald, S.J., Dowle, M.D., Harrison, L.A., Shah, P., Johnson, M.R., Inglis, G.G., Clarke, G.D., Smith, R.A., Humphreys, D., Molloy, C.R.*, et al.* (2001). The discovery of a potent, intracellular, orally bioavailable, long duration inhibitor of human neutrophil elastase--GW311616A a development candidate. Bioorg Med Chem Lett *11*, 895-898.

Malergue, F., Galland, F., Martin, F., Mansuelle, P., Aurrand-Lions, M., and Naquet, P. (1998). A novel immunoglobulin superfamily junctional molecule expressed by antigen presenting cells, endothelial cells and platelets. Mol Immunol *35*, 1111-1119.

Ody, C., Jungblut-Ruault, S., Cossali, D., Barnet, M., Aurrand-Lions, M., Imhof, B.A., and Matthes, T. (2007). Junctional adhesion molecule C (JAM-C) distinguishes CD27+ germinal center B lymphocytes from non-germinal center cells and constitutes a new diagnostic tool for B-cell malignancies. Leukemia *21*, 1285-1293.

Vincent, J.L., de Mendonca, A., Cantraine, F., Moreno, R., Takala, J., Suter, P.M., Sprung, C.L., Colardyn, F., and Blecher, S. (1998). Use of the SOFA score to assess the incidence of organ dysfunction/failure in intensive care units: results of a multicenter, prospective study. Working group on "sepsis-related problems" of the European Society of Intensive Care Medicine. Crit Care Med *26*, 1793-1800.
